# Selective Modification of Ribosomally Synthesized and Post‐Translationally Modified Peptides (RiPPs) through Diels–Alder Cycloadditions on Dehydroalanine Residues

**DOI:** 10.1002/chem.201902907

**Published:** 2019-09-09

**Authors:** Reinder H. de Vries, Jakob H. Viel, Ruben Oudshoorn, Oscar P. Kuipers, Gerard Roelfes

**Affiliations:** ^1^ Stratingh Institute for Chemistry University of Groningen Nijenborgh 4 9747 AG Groningen The Netherlands; ^2^ Department of Molecular Genetics Groningen Biomolecular Sciences and Biotechnology Institute University of Groningen Nijenborgh 7 9747 AG Groningen The Netherlands

**Keywords:** bio-orthogonal chemistry, Diels–Alder, late stage chemical modification, RiPPs, thiopeptides

## Abstract

We report the late‐stage chemical modification of ribosomally synthesized and post‐translationally modified peptides (RIPPs) by Diels–Alder cycloadditions to naturally occurring dehydroalanines. The tail region of the thiopeptide thiostrepton could be modified selectively and efficiently under microwave heating and transition‐metal‐free conditions. The Diels–Alder adducts were isolated and the different site‐ and *endo*/*exo* isomers were identified by 1D/2D ^1^H NMR. Via efficient modification of the thiopeptide nosiheptide and the lanthipeptide nisin Z the generality of the method was established. Minimum inhibitory concentration (MIC) assays of the purified thiostrepton Diels–Alder products against thiostrepton‐susceptible strains displayed high activities comparable to that of native thiostrepton. These Diels–Alder products were also subjected successfully to inverse‐electron‐demand Diels–Alder reactions with a variety of functionalized tetrazines, demonstrating the utility of this method for labeling of RiPPs.

## Graphical abstract

**Late‐stage chemical editing** of RiPPs is achieved by a Diels–Alder reaction of the naturally occurring dehydroalanine residues with cyclopentadiene.

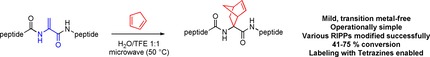

Ribosomally synthesized and post‐translationally modified peptides (RiPPs),^1^ such as thiopeptides^2^, ^3^, ^4^, ^5^ and lanthipeptides^1^, ^6^ have attracted attention as potential alternatives to small‐molecule antibiotics because of their high activity against a broad range of bacteria and low level of resistance development.^7^, ^8^ Yet chemical editing of these peptides is necessary in order to mitigate their poor pharmacological properties and to make them suitable for clinical application and to synthesize analogues and derivatives for the study of their mechanism of action. Over the years, progress has been made towards late‐stage chemical modification of antimicrobial peptides isolated from producing strains, although achieving (site) selective derivatization of these structurally diverse and complex natural products often poses a major synthetic challenge.^9^

Many thiopeptides and lanthipeptides contain one or more uniquely reactive dehydroamino acids such as dehydroalanine (Dha) and dehydrobutyrine (Dhb), which are the result of post‐translational enzymatic dehydration of Ser and Thr residues, respectively.^10^ The electrophilic nature of dehydroamino acids has made them attractive functionalities for biorthogonal reactions.^11^, ^12^, ^13^, ^14^, ^15^, ^16^, ^17^, ^18^, ^19^, ^20^ In recent years, these dehydroamino acids have emerged as interesting targets for the late‐stage modification of RiPPs, through Michael additions,^21^, ^22^, ^23^, ^24^ hydrogenations,^25^ cross‐coupling reactions,^26^, ^27^ photoredox catalysis,^28^ cyclopropanations,^29^ and 1,3‐dipolar cycloadditions.^30^ These studies have highlighted the potential of dehydroamino acid modification in RiPPs, but also illustrate the challenge of achieving selectivity due to the high structural complexity of RiPPs and the difficulties of discriminating between the various dehydroamino acids present.

Here, we now report the Diels–Alder reaction with cyclopentadiene as a mild and selective modification reaction for of dehydroalanine residues in antimicrobial RiPPs (Scheme 1). Furthermore, the unactivated, strained alkene in the formed norbornene product could be employed in Inverse Electron Demand Diels–Alder (IEDDA, “click”) reactions with tetrazines (Scheme 1), a popular labeling tool in chemical biology.^31^

**Scheme 1:**
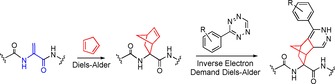
Two‐step labeling of dehydroalanines in RiPPs through a Diels–Alder and IEDDA sequence.

As a starting point, the Diels–Alder reaction between cyclopentadiene and a protected dehydroalanine substrate (**1**) was studied (Supporting Information, SI‐7). In previous studies only anhydrous conditions and also high temperatures had been reported for this reaction.^32^ The Diels–Alder reaction is known to be significantly accelerated in water.^33^ Indeed, appreciable conversion was observed in water at room temperature after 48 h, whereas no product was observed when using dichloromethane as solvent (SI‐7).

Next, different co‐solvents that are tolerated by peptides were tested in order to help solubilize the cyclopentadiene and thereby increase the conversion. It was found that 2,2,2‐trifluoroethanol (TFE) gave the best results, likely due to its mild Brønsted acidity, which can give rise to activation of the dienophile.^34^ Using 20 mol % Sc(OTf)_3_ to activate the dienophile improved the conversion further, up to 88 % after 48 h with 10 equiv. cyclopentadiene.

The *endo*/*exo* ratio was ≈40:60 in all cases, which is in agreement with previous reports about the secondary orbital interactions between this particular Dha substrate (**1**) and cyclopentadiene.^32^ 1,3‐cyclohexadiene, 1,3‐dimethylbutadiene, and furan were also evaluated as dienes, but did not give any conversion at room temperature (SI‐7).

The conditions established with the protected Dha substrate appeared suitable for modification of the thiopeptide thiostrepton (Figure 1 A), given its high solubility in TFE. During initial screening and subsequent LC‐MS analysis, it was found that addition of Sc(OTf)_3_ did not give rise to increased conversions compared to reactions performed without the scandium salt.

**Figure 1:**
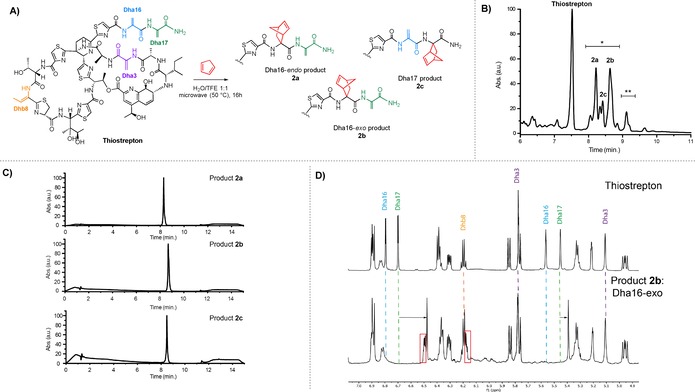
A) Scheme depicting the Diels–Alder reaction between thiostrepton and cyclopentadiene to give the corresponding products **2 a**–**c**. Conditions: 1 mm thiostrepton and 0.6 m freshly distilled cyclopentadiene in 1 mL H_2_O/TFE 1:1, microwave‐assisted heating at 50 °C for 16 h. B) Zoom in of LC‐MS chromatogram of the crude product showing products **2 a**–**c** (*=single modification, **=double modification). C) Full LC‐MS chromatograms of purified products **2 a**–**c**. D) Stacked ^1^H NMR spectra of thiostrepton (top) and product **2 b** (bottom), showing the region between 5.0 and 7.0 ppm.

On the contrary, the transition metal free conditions gave rise to the cleanest transformations, giving mainly single‐ and double modified thiostrepton (Figure 1 B). After seven days of reaction time (while adding freshly distilled cyclopentadiene daily) 64 % conversion to single‐ and double‐modified thiostrepton was obtained as based on peak integration of the starting material and the products in analytical HPLC.

Performing the reaction at 50 °C in a microwave reactor greatly improved the conversion to 72 % after only 16 h of reaction time, compared to 28 % conversion after 16 h at room temperature and 50 % conversion when heating the reaction at 50 °C in an oil bath. A mixture of single‐ and double‐modified products was obtained and the starting material and the products proved to be stable under the microwave conditions. Even hydrolytic cleavage of the Dha‐tail, which is a common side reaction in thiostrepton modification,^23^ was not observed.

The reaction was performed on a 25 mg scale, after which the three major single modified products (**2 a**–**c**) were isolated using preparative HPLC (Figure 1 C). Products **2 a**–**c**, obtained as mixtures of diastereomers that could not be separated, were analyzed by NMR. When comparing the ^1^H NMR spectra of unmodified thiostrepton and the products, with particular focus on the region between 5.00 ppm and 7.00 ppm (Figure 1 D, only showing product **2 b** for this example, see SI‐10–12, 33–37 for all spectra) it can be seen that the methylene signals of Dha3 (purple) and Dhb8 (yellow) are conserved in product **2 b**. From the two sets of signals originating from the methylenes in the tail, that is, Dha16 (blue) and Dha17 (green), one set of signals has disappeared and the other has shifted upfield, indicating that the reaction has taken place in the tail region of thiostrepton. Moreover, the appearance of two doublets of doublets (red) is characteristic for the formation of the alkene of norbornene. The NMR spectra of **2 a** and **2 c** showed similar changes in signals (SI‐11, 33–37).

Using ^1^H‐^1^H TOCSY NMR, products **2 a** and **2 b** were both identified as Dha16‐modified thiostrepton (see SI‐11 for a detailed explanation). By comparing the methylene signals of Dha17 in products **2 a** and **2 b**, thereby taking into account the shielding effect of the newly formed carbon‐carbon double bond in the norbornene, it was established that product **2 a** is Dha16‐*endo* and product **2 b** is Dha16‐*exo* (see SI‐11). In a similar manner, using ^1^H NMR and ^1^H‐^1^H TOCSY NMR techniques, product **2 c** could be identified as Dha17‐modified thiostrepton (SI‐12).

To further demonstrate the selectivity for the tail region, a truncated variant of thiostrepton (**3**) was synthesized via selective base‐mediated cleavage of Dha17 from the tail of thiostrepton using Et_2_NH, leaving only Dha16 as a reactive site (Scheme 2, SI‐6).^23^ When **3** was subjected to the optimized reaction conditions, only two major single modified products (**4 a** and **4 b**, Scheme 2) were obtained. Using analytical HPLC a 41 % total conversion was observed (SI‐13). Both products were isolated as mixtures of diastereomers and identified (SI‐16–17) as *endo*‐ (**4 a**) and *exo* (**4 b**) isomers of Dha16‐modified **3** (SI‐13) using NMR analysis analogous to the identification of products **2 a**–**c**.

**Scheme 2:**
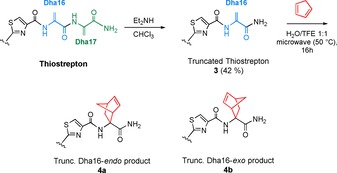
Synthesis and Diels–Alder reaction of truncated thiostrepton (**3**).

Collectively, these results show that the reaction is highly selective for the tail region of thiostrepton. Also, the LC‐MS UV signal areas of products **2 a** and **2 b** compared to product **2 c** (Figure 1 B) indicate a significant preference for modification at Dha16, which can be explained by the fact that this residue is the most electron‐poor site due to the neighboring thiazole15 and Dha17, both electron‐withdrawing moieties.

The scope of the reaction was evaluated by performing the reaction on different RiPPs. The Diels–Alder reaction of cyclopentadiene and the thiopeptide nosiheptide was performed under the optimized conditions and after microwave‐assisted heating at 50 °C for 32 h a conversion of 75 % to single modified nosiheptide was observed (Scheme 3 A, SI‐18). The commercial nosiheptide starting material contained a small amount of nosiheptide that lacks the terminal Dha, having a terminal amide instead. The product of the reaction of this impurity with cyclopentadiene was not observed in the LC‐MS analysis, confirming that the reaction is selective for the terminal Dha over the internal Dhb residue, which is consistent with the results obtained using thiostrepton and **3**.

**Scheme 3:**
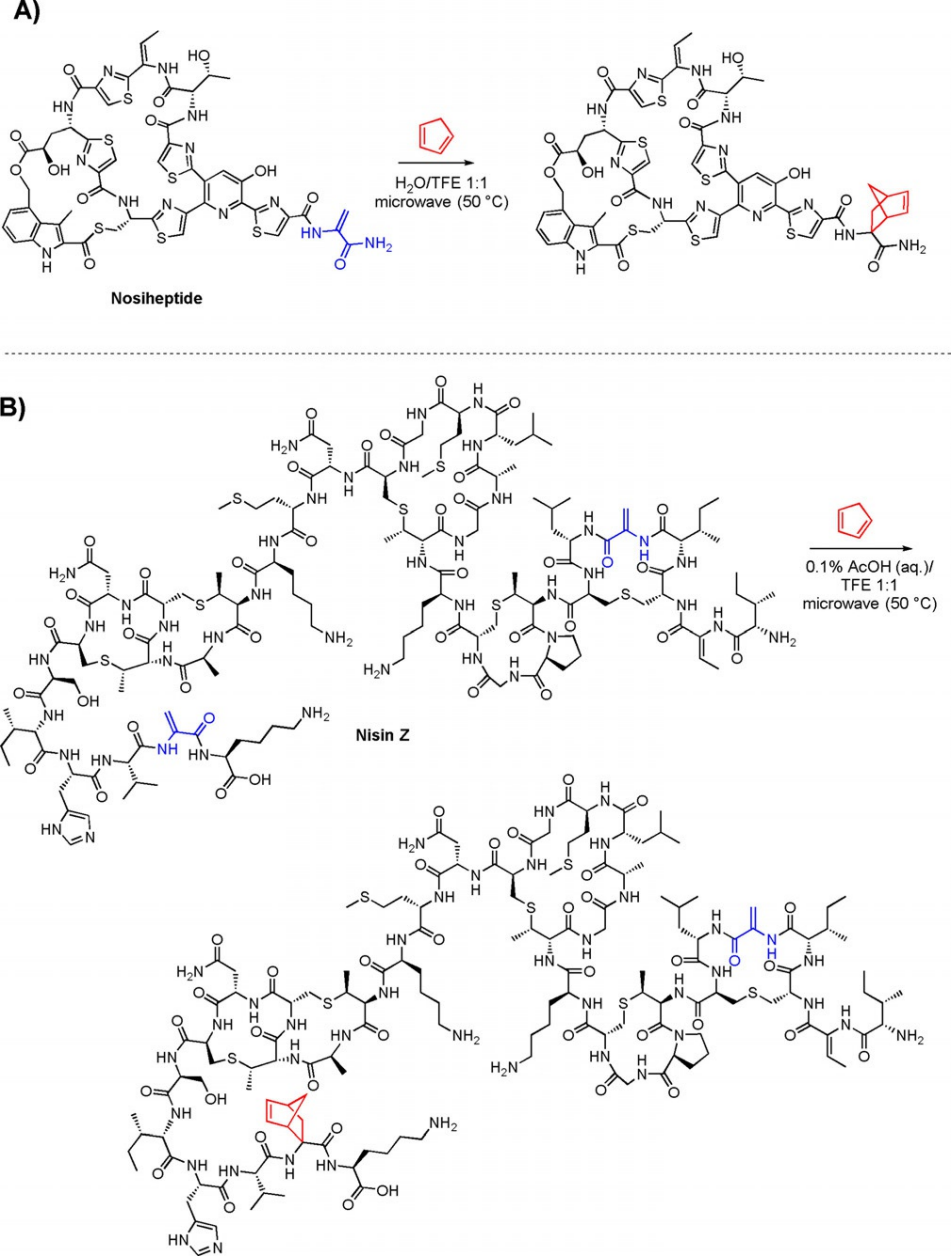
Diels–Alder reactions of cyclopentadiene with A) the thiopeptide nosiheptide and B) the lanthipeptide nisin Z. For nisin Z only one of the possible products is shown.

The reaction between cyclopentadiene and the lanthipeptide nisin Z was investigated next (Scheme 3 B). In this case, the same conditions as for the thiopeptides were used, except for the substitution of ddH_2_O for 0.1 % AcOH (aq.) due to solubility‐ and stability issues of nisin at pH>5. In addition to the inevitable, but well‐documented addition of water to Dha in nisin,^35^ a 52 % conversion to single Diels–Alder modified product was observed after 16 h of microwave irradiation at 50 °C (SI‐19–20). For nisin Z, which bears one Dhb and two Dha residues, the site selectivity could not be determined due to poor separation of isomers on LC‐MS and HPLC. However, good stabilities under microwave irradiation were observed for both nosiheptide and nisin Z, demonstrating the general applicability of our approach for the modification of Dha‐containing RiPPs.

Previous studies have shown that modification of the tail region of thiostrepton does not severely impact its activity.^23^, ^26^ To confirm that this is also true for the norbornene modifications, thiostrepton and purified derivatives **2 a**–**c**, **3**, and **4 a**,**b** were tested against *S. aureus* (ATCC29213) and *E. faecalis* (ATCC29212) strains in a MIC‐assay (SI‐21). The results (Table 1) show that all derivatives have excellent antimicrobial activity, with a MIC value that is within one order of magnitude compared to native thiostrepton for both strains. Moreover, variations in activity towards both strains and between the different site‐ and *endo*/*exo* isomers remained limited to a factor of 4. The activity of **3** also very closely resembles that of thiostrepton, showing that even removing part of the tail region has little effect on its activity.

**Table 1:** MIC‐assay results of Diels–Alder analogues of thiostrepton against *S. aureus* and *E. faecalis*.

Antibiotic	MIC [μg mL^−1^] against *S. aureus*	MIC [μg mL^−1^] against *E. faecalis*
Vancomycin	1	4
Thiostrepton	0.5	0.5
**2 a**	2	2
**2 b**	2	2
**2 c**	2	1
**3**	0.5	1
**4 a**	4	2
**4 b**	2	2

The selective incorporation of the norbornene functionality in the tail of thiostrepton while leaving the inherent activity intact enables further derivatization through IEDDA click reactions with tetrazines.^31^ Purified **2 a** was treated with di‐2‐pyridyl tetrazine (**5**) in H_2_O/ACN 1:1 at room temperature (Figure 2 A) and after overnight reaction full conversion to singly labeled dihydropyridazine (*m*/*z=*1938) and pyridazine (*m*/*z=*1936) products was observed by MALDI‐TOF MS of the crude reaction mixture (Figure 2 B). As a control, unmodified thiostrepton was subjected to the same conditions, after which only starting material (*m*/*z=*1664, Figure 2 B inset) was observed, illustrating the high chemoselectivity for the norbornene moiety over the other unsaturated motifs in thiostrepton.

**Figure 2:**
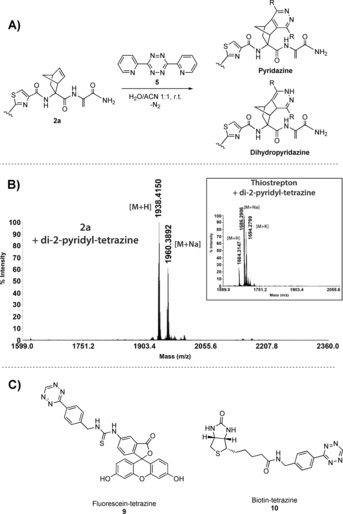
A) IEDDA reaction of norbornene‐modified thiostrepton with di‐2‐pyridyl tetrazine (**5**). B) MALDI‐TOF MS spectra of IEDDA reaction of di‐2‐pyridyl tetrazine with **2 a** and control reaction with unmodified thiostrepton (inset). C) Structures of fluorescein‐tetrazine (**9**) and biotin‐tetrazine (**10**).

Next, the IEDDA reaction with a range of different functionalized tetrazines was investigated. An amine‐functionalized tetrazine building block (**8**)^36^ was derivatized with a fluorescein‐ (**9**) or biotin (**10**) moiety (Figure 2 C). MALDI‐TOF MS showed efficient labeling of **2 b** with both tetrazines using the same conditions as described above (SI‐22).

A BODIPY‐labeled tetrazine (**12**) with fluorescence turn‐on properties was synthesized using a procedure by Carlson et al. with minor modifications (SI‐3).^37^ The fluorescence of **12** is quenched almost completely by the tetrazine motif. However, this effect is lifted upon reaction of the tetrazine in the IEDDA click reaction (Figure 3 A).^37^ Upon addition of **2 a** to a solution of **12**, fluorescence measurements indeed showed a rapid increase in fluorescence compared to an identical solution of **12** where only DMSO was added as a control (SI‐23). This fluorescence turn‐on effect could even be visualized by shining UV light (365 nm) on the undiluted samples (Figure 3 B), which shows the potential for using this two‐step labeling method in the detection of new Dha‐containing peptides.

**Figure 3:**
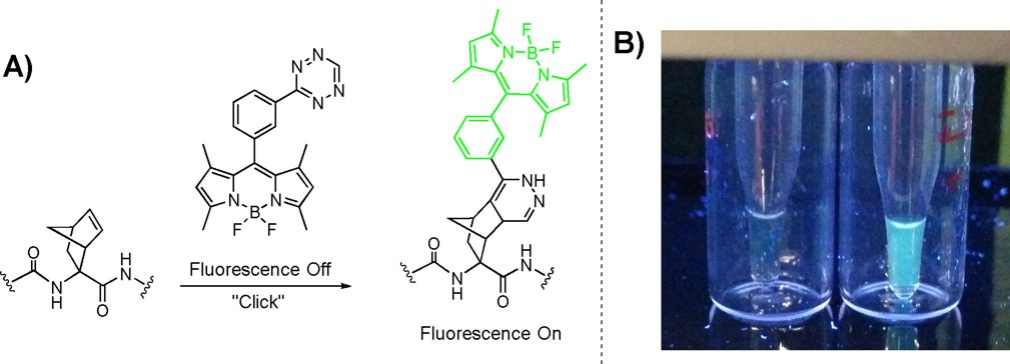
A) Scheme depicting fluorescence turn‐on of BODIPY‐tetrazine upon click reaction with the norbornene‐modified peptide. B) Image showing fluorescence under UV light (365 nm) for DMSO control (left) and click reaction with **2 a** (right).

We have established the Diels–Alder reaction as a powerful tool for efficient and selective late‐stage chemical editing of peptide antibiotics. This approach, which only requires cyclopentadiene as a reagent and microwave‐assisted heating, allows for straightforward and transition‐metal‐free installation of the norbornene functionality on these complex natural products by reacting with the naturally occurring Dha residues under mild conditions. Especially attractive is the possibility of employing the norbornene product in Inverse Electron Demand Diels–Alder reactions with tetrazines, which gives access to a variety of new semisynthetic derivatives. Additionally, the norbornene moiety could potentially be used in other labeling reactions.^38^, ^39^ These results demonstrate the potential of this methodology for the tailoring of RiPPs.

## Conflict of interest

The authors declare no conflict of interest.

## Supporting information

As a service to our authors and readers, this journal provides supporting information supplied by the authors. Such materials are peer reviewed and may be re‐organized for online delivery, but are not copy‐edited or typeset. Technical support issues arising from supporting information (other than missing files) should be addressed to the authors.

Supplementary
