# Supplementary material for: Selective Modification of Ribosomally Synthesized and Post‐Translationally Modified Peptides (RiPPs) through Diels–Alder Cycloadditions on Dehydroalanine Residues
Source: Chemistry. 2019 Sep 9;25(55):12698–702. doi: 10.1002/chem.201902907 (PMC6790694; doi:10.1002/chem.201902907)
Supplement: Supplementary file 1 — Supplementary [file CHEM-25-12698-s001.pdf]

CHEMISTRY
A European Journal

Supporting Information

Selective Modification of Ribosomally Synthesized and Post-Translationally Modified Peptides (RiPPs) through Diels–Alder Cycloadditions on Dehydroalanine Residues

Reinder H. de Vries,[a] Jakob H. Viel,[b] Ruben Oudshoorn,[a] Oscar P. Kuipers,[b] and Gerard Roelfes*[a]

chem_201902907_sm_miscellaneous_information.pdf

Table of Contents

General remarks SI-2
Tetrazine Synthesis Scheme SI-3
Synthetic Procedures SI-4
Truncation of thiostrepton....SI-6
General procedure for Diels-Alder reaction on Methyl 2-(acetamido)acrylate SI-7
Diels-Alder reaction on thiostrepton....SI-8
Diels-Alder reaction on truncated thiostrepton SI-13
Diels-Alder reaction on nosiheptide....SI-18
Diels-Alder reaction on nisin Z SI-19
Biological Activity Assays SI-21
Tetrazine Ligation of thiostrepton Diels-Alder Adducts SI-22
NMR Spectra....SI-24
References....SI-39

General remarks

Chemicals were purchased from Sigma-Aldrich, Acros Organics, TCI Europe, Fluorochem and Activate Scientific and used without further purification unless explicitly specified. Cyclopentadiene was freshly distilled and used immediately. Flash column chromatography was performed on silica gel (Silica gel 60 from Merck, 0.040-0.063 mm, 230-400 mesh). TLC was performed on silica gel (Silica-P flash silica gel from Silicycle, 0.040-0.063 mm , 230-400 mesh). Melting points were recorded on a Büchi B-545 melting point apparatus. ^{1}H-, ^{13}C- and ^{19}F-NMR spectra were recorded on an Agilent 400-MR at 298K spectrometer operating at 400, 101 and 376 MHz respectively. 1D and 2D ^{1}H NMR on thiostrepton and its derivatives was performed on a Brüker Ascend 600 operating at 600 MHz. Chemical shifts in ^{1}H and ^{13}C NMR spectra were internally referenced to solvent signals ($CDCl_3$ at δH = 7.26 ppm, δC = 77.16 ppm; DMSO-d_6 at δH = 2.50 ppm, δC = 39.51 ppm). LC-MS analysis was performed on a Waters Acquity UPLC with TQD mass detector (ESI+). All analysis was performed at 35 °C using a reversed-phase UPLC column (Waters Acquity UPLC BEH C8, 1.7 µm, 2.1 mm x 150 mm). UPLC grade 0.1 % Formic Acid (FA) in H_2O (solvent A) and 0.1 % FA in acetonitrile (solvent B) were used as eluents. Gradient used for thiostrepton and nosiheptide and derivatives: 70 % A to 30 % A over 8 minutes, then to 5 % A over 1 minute (total runtime 15 minutes). All other measurements were done using a gradient of 90 % A to 50 % A over 8 minutes, then to 5 % A over 1 minute (total runtime 15 minutes). High-resolution mass spectrometry was performed on a LTQ Orbitrap XL spectrometer (ESI^+). MALDI-TOF MS was performed on an Applied Biosystems 4800 plus TOF/TOF analyzer. Reversed phase HPLC was performed on a Shimadzu HPLC system equipped with LC-20AD solvent chromatographs, a DGU-20A3 degasser unit, a SIL-20A autosampler, a SPD-M20A PDA detector, a CTO-20A column oven, a CBM-20A system controller and a FRC-10A fraction collector. Analysis was performed on a Waters XBridge C8 column (4.6 x 250 mm, particle size 3.5 µm) using a flow of 0.5 mL/min. Preparative HPLC was performed on a Waters XBridge prep C8 column (10 x 150 mm, particle size 5 µm) using a flow of 1.5 mL/min.

Tetrazine Synthesis Scheme

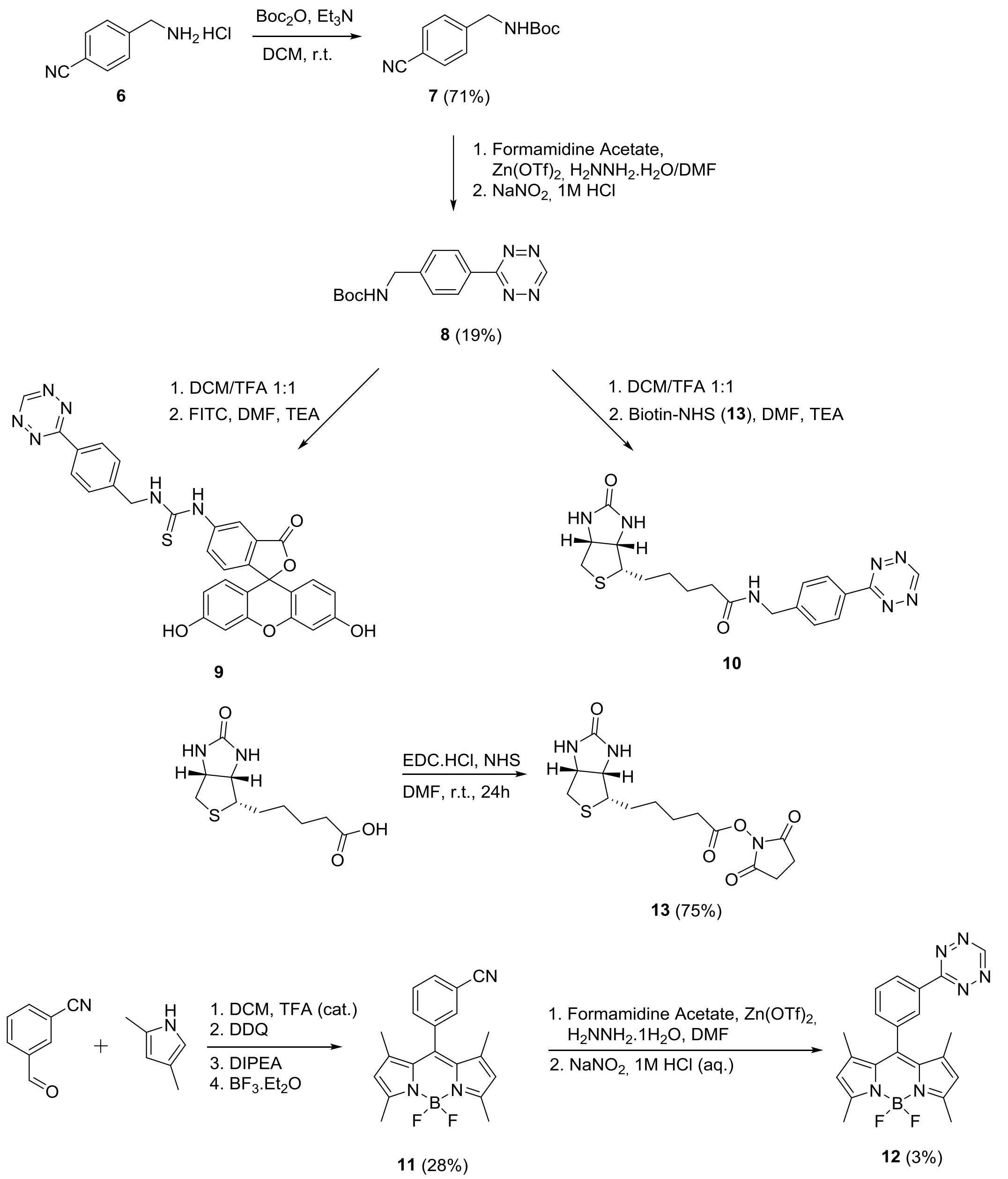

Synthetic Procedures

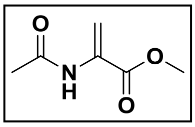

Methyl 2-(acetamido)acrylate (1):[1] 5.00 g (84.7 mmol) acetamide, 7 mL (77.5 mmol) methyl pyruvate, a catalytic amount of p-TsOH and a catalytic amount of 4-methoxyphenol were dissolved in 150 mL toluene. The flask was equipped with a Dean-Stark trap and the mixture was heated under reflux for 20 hours. The mixture was then concentrated in vacuo and the residue was taken up in 300 mL DCM. The organic phase was washed with 300 mL $NaHCO_3$ (sat. aq.) and 300 mL H_2O. The organic layer was then dried over $MgSO_4$, filtered and concentrated in vacuo to yield yellow crystals, which were further purified by column chromatography (SiO_2, heptane/EtOAc 4:1 -> 1:1). 5.13 g (35.8 mmol, 47 %) of white crystals were obtained. Melting point: 52.5-54 °C (Lit.: 48 °C). ^{1}H NMR (400 MHz, $CDCl_3$) δ 7.71 (s, 1H), 6.60 (s, 1H), 5.88 (d, J = 1.5 Hz, 1H), 3.85 (s, 3H), 2.13 (s, 3H). ^{13}C NMR (101 MHz, $CDCl_3$) δ 168.9, 164.8, 131.0, 108.8, 53.1, 24.8.

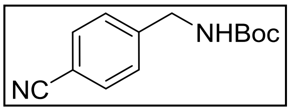

***tert*-butyl (4-cyanobenzyl)carbamate (7):**[2] To 250 mg (1.48 mmol) 4-(aminomethyl) benzonitrile hydrochloride in 5 mL DCM under N_2 atmosphere was added 0.41 mL (2.96 mmol) Et_3N, after which a clear solution was obtained. The mixture was cooled to 0 °C and 0.39 g (1.78 mmol) Boc_2O was added. After stirring for 5 minutes at 0 °C the mixture was allowed to warm to r.t. and was stirred overnight under N_2 atmosphere. The solvent was evaporated and the residue was redissolved in 10 mL Et_2O. The ethereal layer was washed with 2x5mL 0.5 M HCl (aq.), after which the combined aqueous layers were back-extracted with 10 mL Et_2O. The combined organic layers were then washed with 2x5 mL $NaHCO_3$ (sat. aq.) and 5 mL brine. After drying over $MgSO_4$ the solvent was evaporated, yielding a white solid, which was further purified by recrystallization from petroleum ether 40-65 (200 mL solvent used). 244 mg (1.05 mmol, 71 %) white crystals were obtained. Melting point: 109-110 °C (Lit.: 106-108 °C). ^{1}H NMR (400 MHz, $CDCl_3$) δ 7.61 (d, J = 8.2 Hz, 2H), 7.38 (d, J = 8.1 Hz, 2H), 5.01 (s, 1H), 4.36 (d, J = 6.2 Hz, 2H), 1.45 (s, 9H). ^{13}C NMR (151 MHz, $CDCl_3$) δ 156.0, 144.8, 132.5, 127.9, 118.9, 111.2, 80.2, 44.3, 28.5. LC-MS (ESI$^+$) *m/z*: 233.2 $[M+H]^+$, 218.1 $[M-Me+H]^+$, 133.1 $[M-Boc+H]^+$.

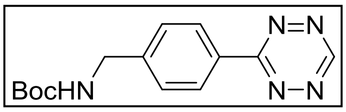

***tert*-butyl (4-(1,2,4,5-tetrazin-3-yl)benzyl)carbamate (8):**[3] 58 mg (0.25 mmol) *tert*-butyl (4-cyanobenzyl)carbamate, 46 mg (0.125 mmol) $Zn(OTf)_2$ and 0.26 g (2.5 mmol) formamidine acetate were added to a microwave vial. The vial was sealed, after which 0.2 mL DMF was added, followed by 0.61 mL (12.5 mmol) hydrazine monohydrate. The mixture was left to stir at 40 °C for 72 hours, after which the mixture was allowed to cool to room temperature. Then, 345 mg (5 mmol) $NaNO_2$ in 5 mL H_2O was added slowly to the mixture. 1M HCl (aq.) was then added dropwise until pH ≤ 3 and bubbling ceased. The aqueous layer was then extracted with 5x20 mL EtOAc, the combined organic layers were dried over $MgSO_4$ and the solvent was evaporated. The purple residue was purified by flash column chromatography (SiO_2, n-hexane/EtOAc 7:1) to give 14 mg (0.048 mmol, 19 %) of a purple solid. ^{1}H NMR (400 MHz, $CDCl_3$) δ 10.21 (s, 1H), 8.60 (d, J = 8.3 Hz, 2H), 7.52 (d, J = 8.1 Hz, 2H), 4.99 (s, 1H), 4.45 (d, J = 5.5 Hz, 2H), 1.48 (s, 9H). ^{13}C NMR (151 MHz, $CDCl_3$) δ 166.5, 157.9, 156.1, 144.8, 130.7, 128.8, 128.3, 80.1, 44.5, 28.6.

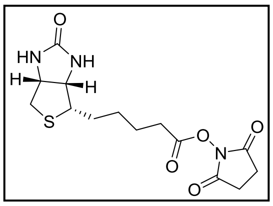

Biotin-NHS (13):[4] 250 mg (1.02 mmol) D-Biotin and 189 mg (1.64 mmol) N-hydroxy succinimide were added to dry DMF under a N_2 atmosphere. 255 mg (1.33 mmol) EDC.HCl was added and the mixture was stirred under N_2 atmosphere for 24 hours. The mixture was then poured onto crushed ice and the precipitate was collected by filtration. The resulting white solid was washed with 3x20 mL ice cold H_2O and dried in vacuo overnight. 261 mg (0.77 mmol, 75 %) of a white solid was obtained. Melting point: 200-207 °C (decomp.) (Lit.: 206-207 °C). ^{1}H NMR (400 MHz, DMSO-d6) δ 6.42 (s, 1H), 6.36 (s, 1H), 4.39 – 4.24 (m, 1H), 4.22 – 4.07 (m, 1H), 3.16 – 3.05 (m, 1H), 2.87 – 2.75 (m, 5H), 2.67 (t, J = 7.4 Hz, 2H), 2.58 (d, J = 12.5 Hz, 1H), 1.73 – 1.57 (m, 3H), 1.57 – 1.34 (m, 3H). ^{13}C NMR (101 MHz, DMSO-d6) δ 170.2, 168.9, 162.7, 61.0, 59.2, 55.2, 30.0, 27.8, 27.6, 25.4, 24.3 (1 signal missing due to overlap).

Fluorescein-H-tetrazine (9): *tert*-butyl (4-(1,2,4,5-tetrazin-3-yl)benzyl)carbamate (**8**) was deprotected by dissolving 12 mg (42 µmol) in 5 mL DCM/TFA 1:1 and stirring this solution at r.t. for 10 minutes, followed by evaporation of the solvents and flash column chromatography (SiO_2, DCM/MeOH 9:1), giving 5 mg (27 µmol, 64 %) of (4-(1,2,4,5-tetrazin-3-yl)phenyl)methanamine TFA salt, which was used without further purification. 2.3 mg (9.3 µmol) (4-(1,2,4,5-tetrazin-3-yl)phenyl)methanamine was then dissolved in 50 µL dry DMF, after which 39 µL (0.28 mmol) TEA was added, followed by 4 mg (10.2 µmol) FITC in 50 µL dry DMF. The solution was stirred at r.t. under a N_2 atmosphere overnight and then diluted with 100 µL H_2O/ACN 1:1, filtered through a microfilter and subjected to prep RP-HPLC (solvent A: 0.1 % FA in ACN, solvent B: 0.1 % FA in ddH_2O, gradient 90 % B to 10 % B over 40 minutes). After lyophilization of the appropriate fractions trace amounts of product were obtained (≤ 1 mg). HRMS calcd. $C_{30}H_{21}N_6O_5S^+$ $[M+H]^+$: *m/z* = 577.1289, found: 577.1290.

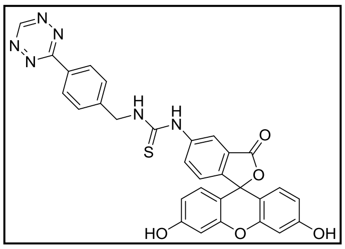

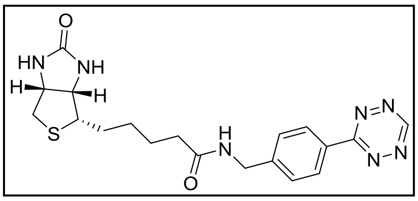

Biotin-H-tetrazine (10): The same procedure as for Fluorescein-H-tetrazine was followed, starting from 2.3 mg (9.3 µmol) (4-(1,2,4,5-tetrazin-3-yl)phenyl)methanamine and 3.5 mg (10.2 µmol) Biotin-NHS (**13**). After prep-HPLC purification and lyophilization, trace amounts (≤1 mg) of a pink solid were obtained. HRMS calcd. $C_{19}H_{24}N_7O_2S^+$ $[M+H]^+$: *m/z* = 414.1707, found: 414.1713.

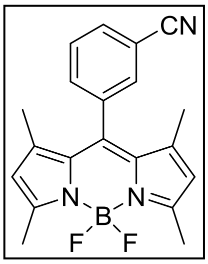

***m*-Cyanophenyl BODIPY (11):**[5] 0.50 g (3.8 mmol) 3-formylbenzonitrile was dissolved in 100 mL DCM. 0.85 mL (8.25 mmol) 2,4-dimethylpyrrole was added, followed by 3 drops of TFA and the mixture was stirred under a N_2 atmosphere for 1 hour until TLC (SiO_2, n-hexane/EtOAc 7:1) indicated full consumption of 3-formylbenzonitrile. 0.86 g (3.8 mmol) DDQ in 100 mL DCM was added, after which the solution turned dark purple immediately. 7.8 mL (44.5 mmol) DIPEA was then added, followed by 8 mL (46 %, 29.8 mmol) $BF_3·OEt_2$ and the mixture was stirred at r.t. under a N_2 atmosphere overnight. 50 mL H_2O was then added and the layers were separated. The aqueous layer was then extracted with 3x150 mL DCM and the combined organic layers were dried over $MgSO_4$ and concentrated in vacuo. The residue was then purified by flash column chromatography (SiO_2, toluene/n-hexane 3:1 -> 9:1 in 3 steps), after which 368 mg (1.06 mmol, 28 %) of a bright orange solid was obtained. Melting point: 233-234 °C. ^1H NMR (400 MHz, $CDCl_3$) δ 7.80 (dt, J = 7.8, 1.4 Hz, 1H), 7.69 – 7.61 (m, 2H), 7.58 (dt, J = 7.8, 1.5 Hz, 1H), 6.01 (s, 2H), 2.56 (s, 6H), 1.35 (s, 6H). ^{19}F NMR (376 MHz, $CDCl_3$) δ -145.98 – -146.60 (m). ^{13}C NMR (151 MHz, $CDCl_3$) δ 156.8, 142.7, 138.1, 136.7, 133.0, 132.9, 132.0, 131.2, 130.3, 122.0, 118.0, 113.7, 14.9 (1 signal missing due to overlap). LC-MS (ESI$^+$) *m/z*: 350.2 $[M+H]^+$, 330.2 $[M-F^-]^+$.

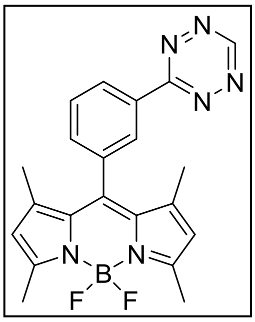

BODIPY-H-tetrazine (12): 98 mg (0.28 mmol) m-cyanophenyl BODIPY, 51 mg (0.14 mmol) $Zn(OTf)_2$ and 0.292 g (2.8 mmol) formamidine acetate were added to a microwave vial. The vial was sealed and 0.35 mL DMF and 0.68 mL (14 mmol) hydrazine monohydrate were added. The mixture was stirred at 60 °C for 24 hours. After allowing the mixture to cool down to r.t., 300 mg (4.35 mmol) $NaNO_2$ in 10 mL H_2O was added slowly. To this solution was then added 1M HCl (aq.) until pH ≤ 3, after which the aqueous solution was extracted with 3x100 mL DCM. The combined organic layers were dried over $MgSO_4$ and the solvent was evaporated. Two times flash column chromatography (SiO_2, n-hexane/EtOAc 4:1 to 1:1 in 2 steps and SiO_2, n-hexane/EtOAc 2:1) yielded 3.9 mg (8.4 µmol, 3 %) of a red brittle solid. ^1H NMR (400 MHz, $CDCl_3$) δ 10.26 (s, 1H), 8.76 (d, J = 7.8 Hz, 1H), 8.66 – 8.59 (m, 1H), 7.77 (t, J = 7.8 Hz, 1H), 7.61 (d, J = 7.6 Hz, 1H), 5.99 (s, 2H), 2.61 (s, 6H), 1.42 (s, 6H). HRMS calcd. $C_{21}H_{19}BFN_6^+$ $[M-F^-]^+$: *m/z* = 385.1748, found: 385.1747.

Truncation of thiostrepton

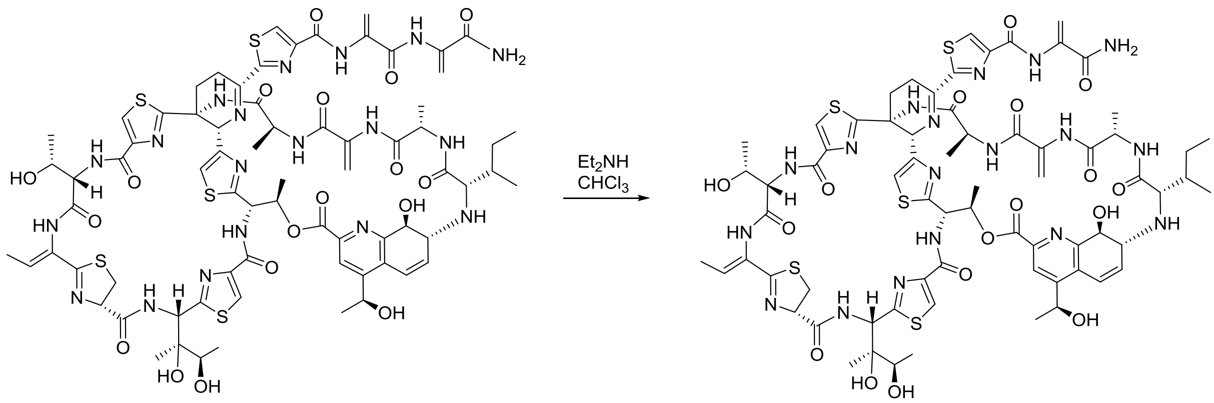

100 mg (0.06 mmol) thiostrepton was dissolved in 6 mL $CHCl_3$. The mixture was cooled to 0 °C and 0.5 mL Et_2NH was added over 5 minutes. The mixture was then allowed to warm up and was stirred at r.t. for 3 hours. TLC (SiO_2, $CHCl_3$/MeOH 9:1) indicated incomplete conversion, so another 0.3 mL Et_2NH was added at once and the mixture was stirred for another 15 minutes. When TLC showed complete conversion, the mixture was co-evaporated with 5 mL toluene. Flash column chromatography (SiO_2, $CHCl_3$/MeOH 0-5% MeOH in 5 steps) afforded 40 mg (25 µmol, 42%) of the desired product that contained a small amount (≤8 %) of thiostrepton truncated at Dha16 (-2Dha), as a colorless brittle solid. ^1H NMR (600 MHz, $CDCl_3$) δ 9.95 (s, 1H), 9.83 (s, 1H), 8.51 (s, 1H), 8.32 (d, J = 9.1 Hz, 1H), 8.27 – 8.23 (m, 2H), 8.11 (s, 1H), 7.80 (s, 1H), 7.58 – 7.55 (m, 2H), 7.54 – 7.52 (m, 1H), 7.46 (s, 1H), 7.30 (s, 1H), 6.91 – 6.86 (m, 2H), 6.85 – 6.80 (m, 1H), 6.74 (d, J = 1.9 Hz, 1H), 6.42 (d, J = 7.5 Hz, 1H), 6.40 – 6.35 (m, 1H), 6.30 (ddd, J = 9.8, 5.5, 1.5 Hz, 1H), 6.19 (q, J = 7.0 Hz, 1H), 5.84 (d, J = 9.1 Hz, 1H), 5.79 – 5.75 (m, 2H), 5.45 – 5.41 (m, 1H), 5.35 – 5.29 (m, 2H), 5.23 – 5.19 (m, 1H), 5.11 (s, 1H), 4.96 (dd, J = 13.4, 8.7 Hz, 1H), 4.80 – 4.73 (m, 1H), 4.68 (d, J = 8.0 Hz, 1H), 4.46 (dd, J = 8.0, 3.3 Hz, 1H), 4.12 – 4.05 (m, 3H), 3.88 – 3.77 (m, 2H), 3.71 (dd, J = 11.4, 8.6 Hz, 1H), 3.63 (dd, J = 5.6, 1.6 Hz, 1H), 3.53 – 3.40 (m, 1H), 3.12 (dd, J = 13.4, 11.4 Hz, 1H), 2.99 (d, J = 6.3 Hz, 1H), 2.98 – 2.85 (m, 1H), 2.26 (td, J = 12.8, 5.8 Hz, 1H), 1.74 (d, J = 6.5 Hz, 3H), 1.62 (d, J = 7.0 Hz, 3H), 1.52 – 1.43 (m, 5H), 1.37 (d, J = 6.4 Hz, 3H), 1.33 (d, J = 6.5 Hz, 3H), 1.20 – 1.16 (m, 8H), 1.10 – 1.05 (m, 1H), 1.00 (d, J = 6.0 Hz, 3H), 0.95 (t, J = 7.4 Hz, 3H), 0.89 (d, J = 6.8 Hz, 3H). HRMS calcd. $C_{69}H_{83}N_{18}O_{17}S_5^+$ $[M+H]^+$: m/z = 1595.4782, found: 1595.4761.

General procedure for Diels-Alder reaction on Methyl 2-(acetamido)acrylate

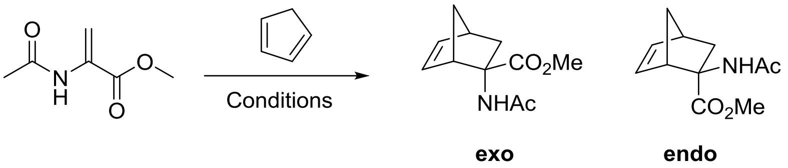

20 mg (0.14 mmol) methyl 2-(acetamido)acrylate was dissolved in 1 mL ddH_2O in a 6 mL vial equipped with a stirring bar. 1 mL of cosolvent and, where appropriate, 0.1 or 0.2 eq. Lewis acid were added. Then, 5 or 10 eq. freshly distilled cyclopentadiene was added and the reaction mixture was stirred at room temperature for 24-50 hours. The reaction mixture was extracted to 5 mL DCM. The organic layer was separated, dried over $MgSO_4$ and concentrated in vacuo. The crude mixture containing starting material and endo- and exo products was then dissolved 0.6 mL $CDCl_3$. ^1H NMR spectra obtained from the crude product were in accordance with previously reported spectra of the endo- and exo products.[5] Conversions and endo/exo ratios were based on relative integrations of known ^1H NMR signals of the starting material and products (entries 1-10) or LC-MS analysis (entries 11-12).

Entry	Diene	Solvent (1:1)	Lewis Acid	Reaction Time	Conversion (%)
1	Cyclopentadiene (5 eq.)	DCM	-	48h	-
2	Cyclopentadiene (5 eq.)	H_2O	-	51h	36 ± 7
3	Cyclopentadiene (5 eq.)	H_2O/DMF	-	51h	41 ± 6
4	Cyclopentadiene (5 eq.)	H_2O/ACN	-	51h	27 ± 2
5	Cyclopentadiene (5 eq.)	H_2O/TFE	-	51h	61 ± 1
6	Cyclopentadiene (5 eq.)	H_2O/TFE	$Sc(OTf)_3$ (10 mol%)	24h	28 ± 1
7	Cyclopentadiene (5 eq.)	H_2O/TFE	$Sc(OTf)_3$ (10 mol%)	48h	43 ± 3
8	Cyclopentadiene (10 eq.)	H_2O/TFE	$Sc(OTf)_3$ (20 mol%)	48h	88 ± 3
9	1,3-Cyclohexadiene (10 eq.)	H_2O/TFE	$Sc(OTf)_3$ (20 mol%)	48h	-
10	2,3-dimethylbutadiene (10 eq.)	H_2O/TFE	$Sc(OTf)_3$ (20 mol%)	48h	-
11	Furan (10 eq.)	H_2O/TFE	-	24h	-
12	Furan (neat)	-	-	24h	-

Diels-Alder reaction on thiostrepton

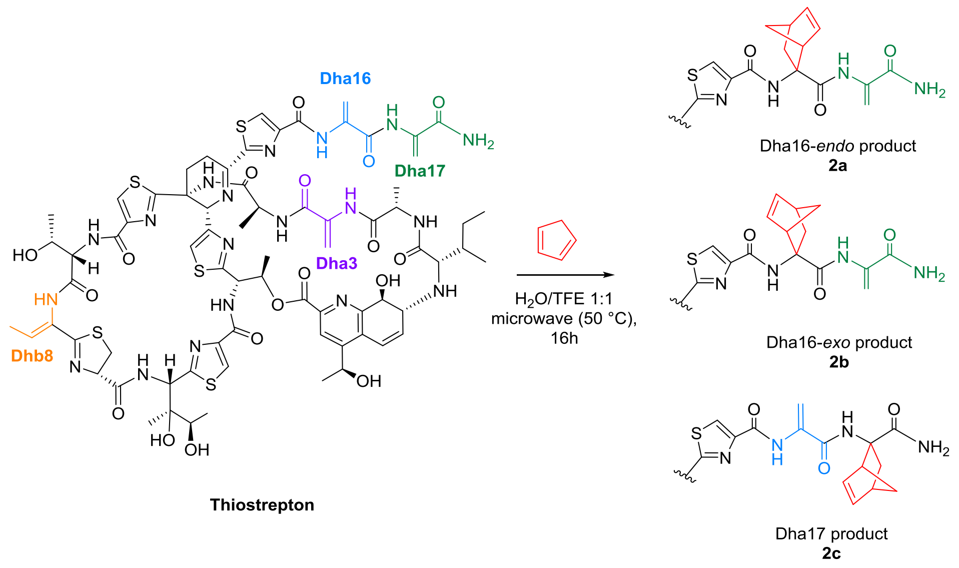

In a typical procedure, 1.7 mg (1 µmol) thiostrepton was dissolved in 0.5 mL TFE. 0.5 mL ddH_2O was added, followed by 50 µL (0.6 mmol) freshly distilled cyclopentadiene. The mixture was stirred overnight in a microwave reactor at 50 °C (50 W power). For LC-MS analysis, samples were prepared by diluting 100 µL of the reaction mixture with 200 µL ddH_2O/ACN 1:1 and filtering over a microfilter (0.2 µm).

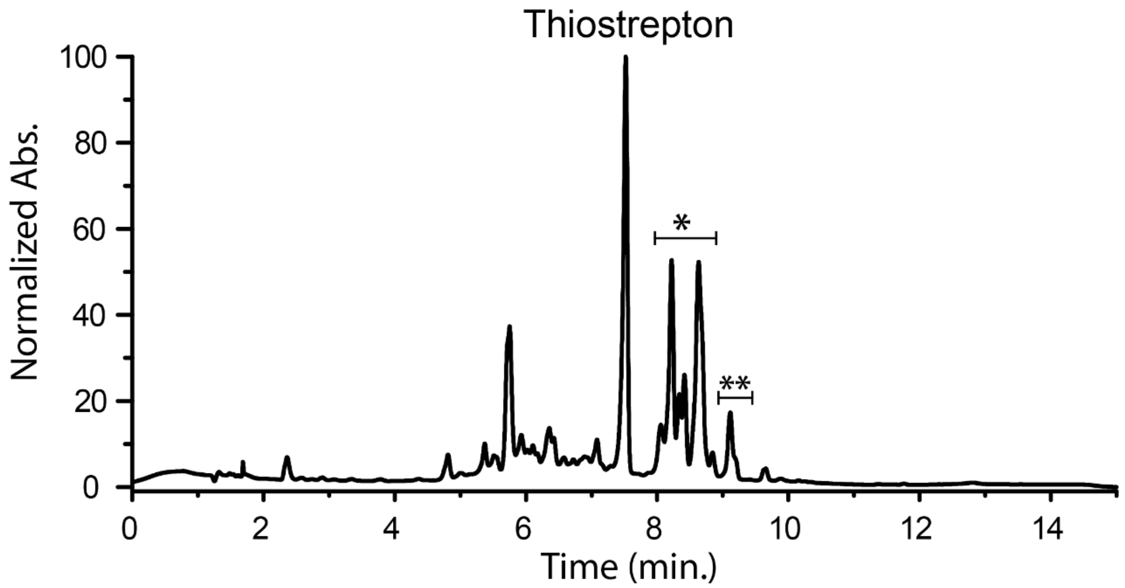

Full LC-MS UV chromatogram (280 nm) of the crude reaction mixture (*single modification, **double modification)

Overall conversion was determined using analytical RP-HPLC (solvent A: 0.1 % FA in ACN, solvent B: 0.1 % FA in ddH_2O, gradient 60 % B to 10 % B over 40 minutes) of the crude mixture, showing a 66 % conversion to single- and 7 % conversion to double modified thiostrepton:

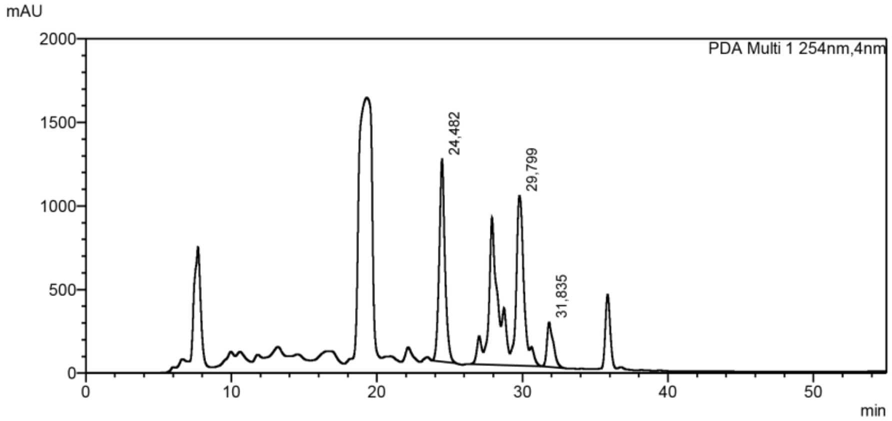

Peak #	Ret. Time (min.)	Area %
**1**	24.5	27.7
**2**	29.8	65.7
**3**	31.8	6.6

Preparative scale and purification: 25 mg (15 µmol) thiostrepton was dissolved in 5 mL TFE and 5 mL H_2O was added. 252 µL (3 mmol) freshly distilled cyclopentadiene was added daily for up to 7 days of total reaction time, during which the mixture was stirred at room temperature. Then, the mixture was transferred to a separatory funnel, and the bottom aqueous layer was separated. The aqueous layer was filtered over a plug of Celite and finally filtered over a microfilter (0.2 µm). The obtained clear filtrate was directly subjected to preparative HPLC.

The crude product was purified by preparative HPLC (solvent A: 0.1 % FA in ACN, solvent B: 0.1 % FA in ddH_2O, gradient 60 % B to 10 % B over 40 minutes). Analysis of the fractions by LC-MS, followed by lyophilization of the combined pure fractions resulted in isolation of the pure Dha16-*endo* (**2a**), Dha16-*exo* (**2b**) and Dha17(**2c**) products as white brittle solids, which were each identified using HRMS.

Prep HPLC fraction collect result:

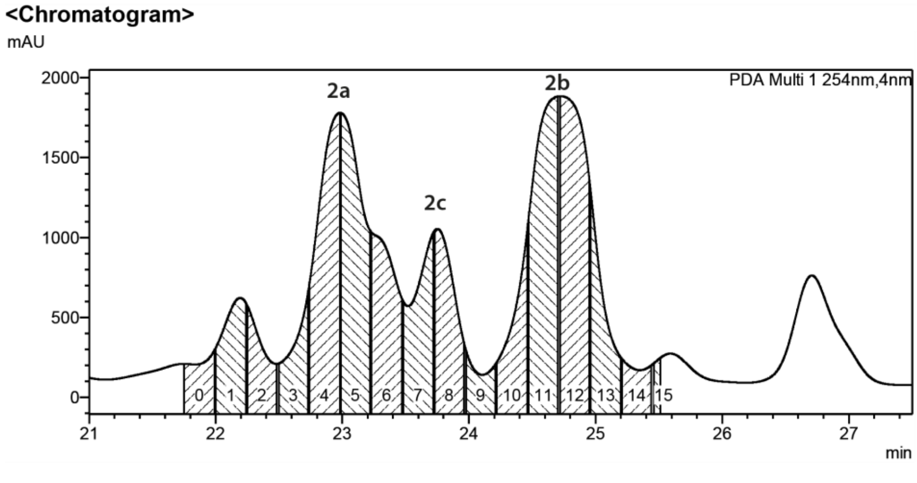

Compound	M_{calc} [M+H]+	M_{found} [M+H]+
**2a**	1730.546	1730.536
**2b**	1730.546	1730.536
**2c**	1730.546	1730.547

NMR analysis and identification of compounds 2a-c: Lyophilized samples of purified compounds **2a-c** (approx. 1 mg) were dissolved in 500 µL $CDCl_3$. ^{1}H NMR, ^{1}H TOCSY NMR and ^{1}H-^{13}C HSQC NMR were used to identify the different regio- and endo/exo isomers.

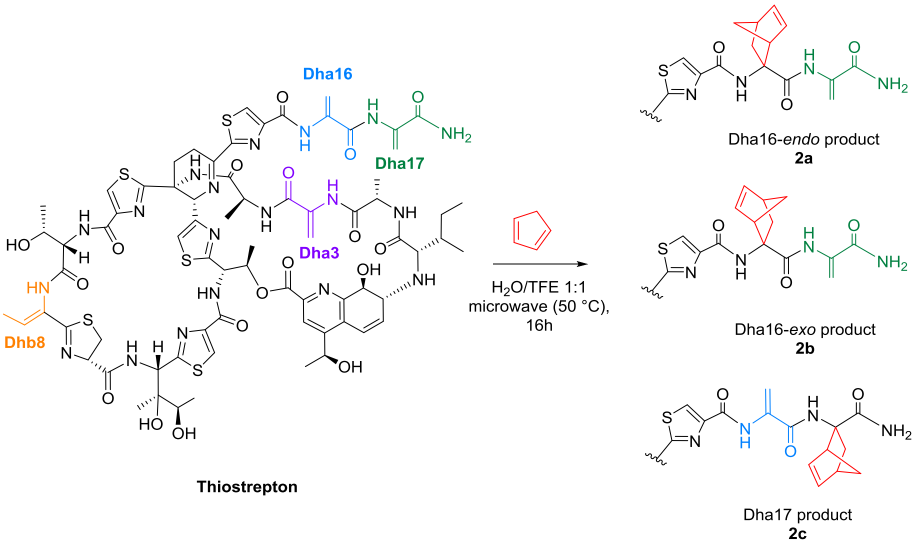

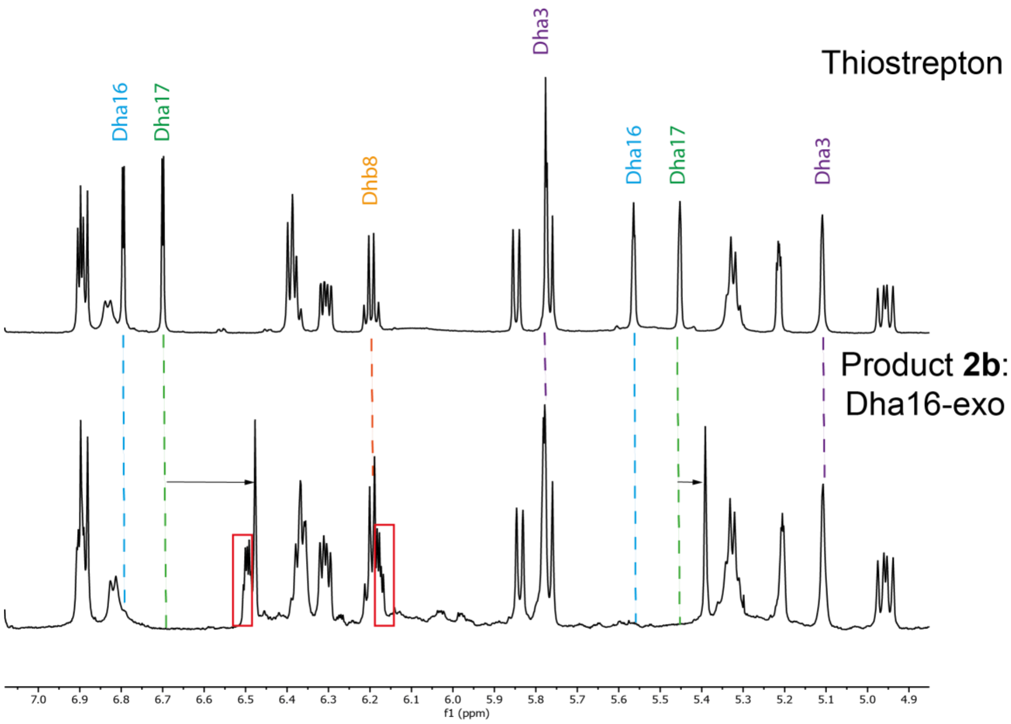

Overlay of ^{1}H NMR spectra of thiostrepton and modified product (2b (Dha16-*exo*)). When comparing the NMR spectra of unmodified thiostrepton and product **2b**, it can be seen that the methylene signals of Dha3 (purple) and Dhb8 (yellow) are conserved in **2b**. From the 2 sets of signals originating from the tail methylenes (Dha16 (blue) and Dha17 (green)) one set of signals has disappeared and the other has shifted upfield in **2b**, indicating that the reaction has taken place in the tail region of thiostrepton. Moreover, the appearance of 2 doublet of doublets (red) is characteristic for the formation of the new carbon-carbon double bond in norbornene.

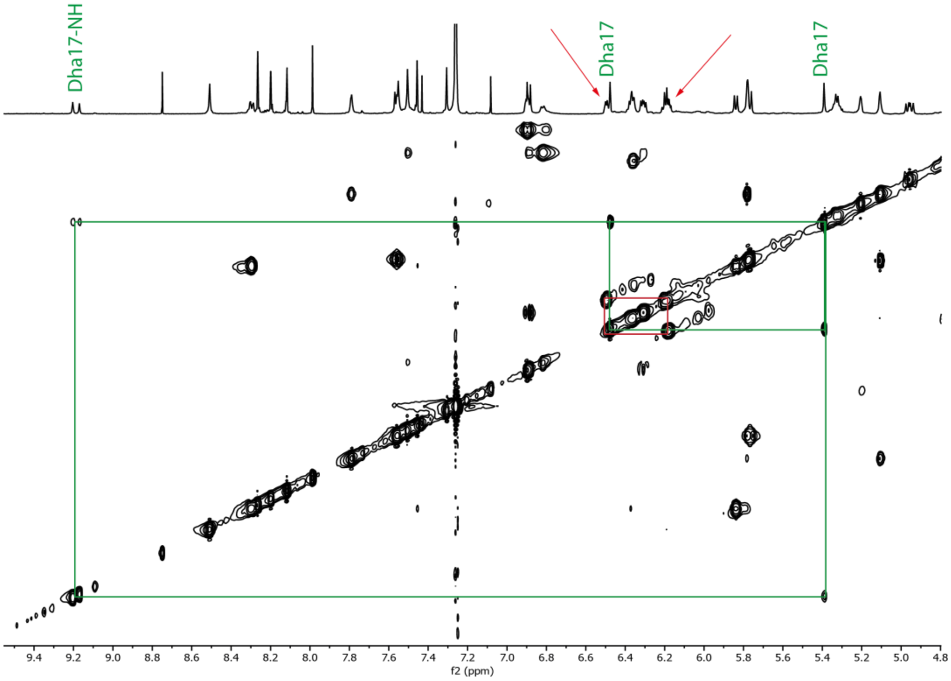

^{1}H-^{1}H TOCSY NMR of compound 2b (Dha16-exo). In the TOCSY NMR of product **2b** the strong correlation between the two shifted methylene signals (6.48 and 5.39 ppm) confirms that they originate from the same Dha residue. Also, a correlation is observed between these methylene signals and the amide N-H (9.19 ppm) that is characteristic for Dha17 (green). This is evidence for Dha17 still being intact but the signal has shifted and, conversely, the reaction has taken place at Dha16. Finally, the strong correlation between the doublet doublets of the norbornene (red) can be observed, which is also consistent with Diels-Alder modification.

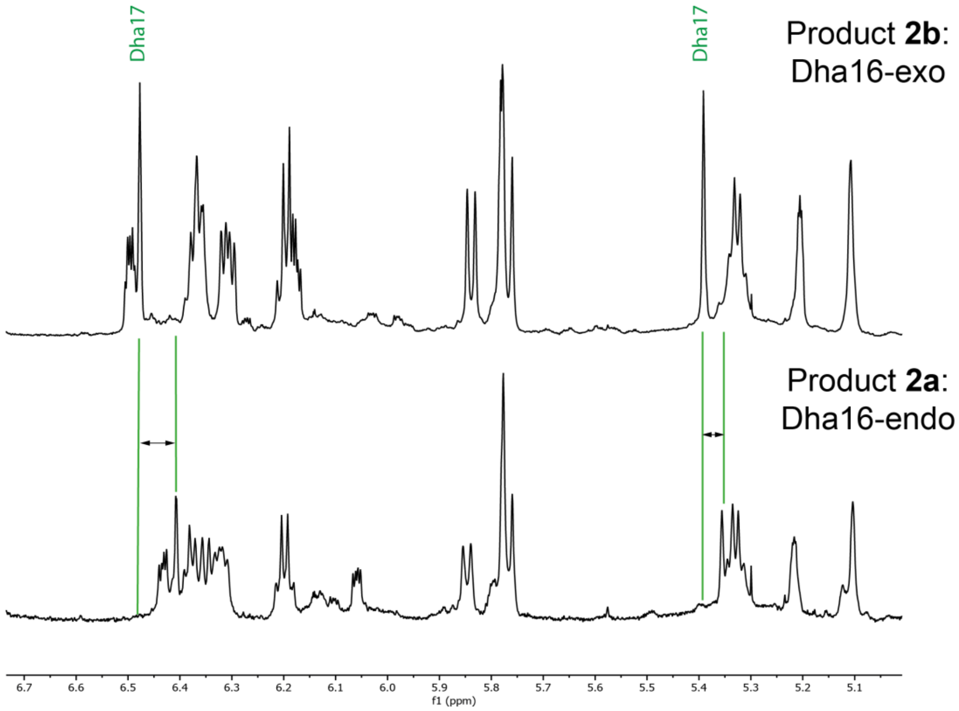

Overlay of ^{1}H NMR spectra of products 2b (Dha16-exo) and 2a (Dha16-endo). Product **2a** was also identified as Diels-Alder modified Dha16 in the same manner as was done using NMR with **2b**. However, the methylene signals of Dha17 were shifted significantly more upfield in product **2a**, indicating they are more shielded by the new double bond in the norbornene and thus residing in the *endo*-position in **2a** and in the *exo*-position in **2b**.

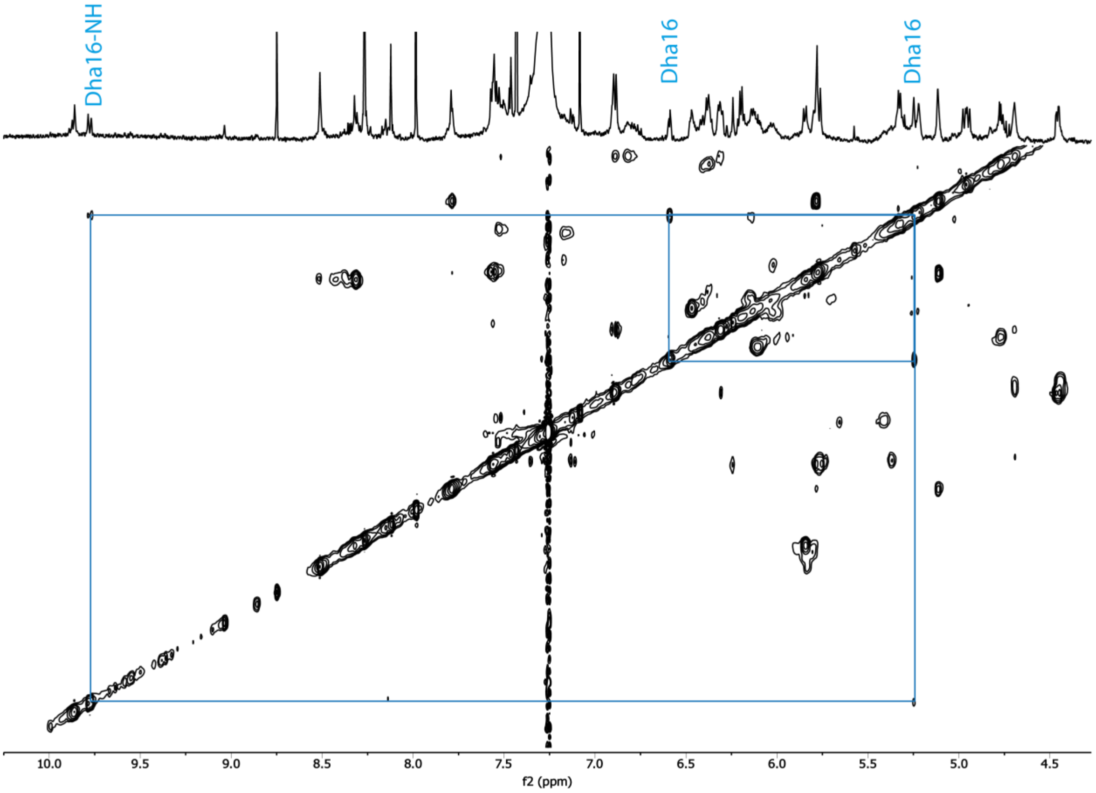

^{1}H-^{1}H TOCSY NMR of product 2c (Dha17). As opposed to the Dha16 products, product **2c** shows a correlation between the shifted methylene signals (6.59 and 5.25 ppm) and the characteristic Dha16 amide proton (9.78 ppm), while on the other hand it can be seen that the Dha17 amide signal (appearing at around 9 ppm in thiostrepton) has disappeared in this case. Taken together, this confirms that **2c** is thiostrepton modified at Dha17. Since only one Dha17 product could be isolated, the comparison between the endo- and exo isomers could not be made.

Diels-Alder reaction on Truncated thiostrepton

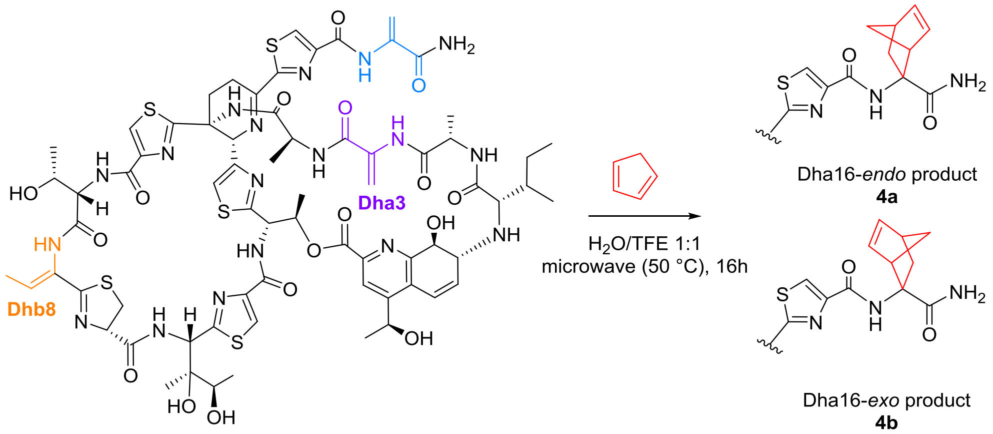

Truncated Thiostrepton (3)

In a typical procedure, 1.6 mg (1 µmol) truncated thiostrepton (**3**) was dissolved in 0.5 mL TFE. 0.5 mL ddH_2O was added, followed by 50 µL (0.6 mmol) freshly distilled cyclopentadiene. The mixture was stirred overnight in a microwave reactor at 50 °C (50 W power). For LC-MS analysis, samples were prepared by diluting 100 µL reaction mixture with 200 µL ddH_2O/ACN 1:1 and filtering over a microfilter (0.2 µm).

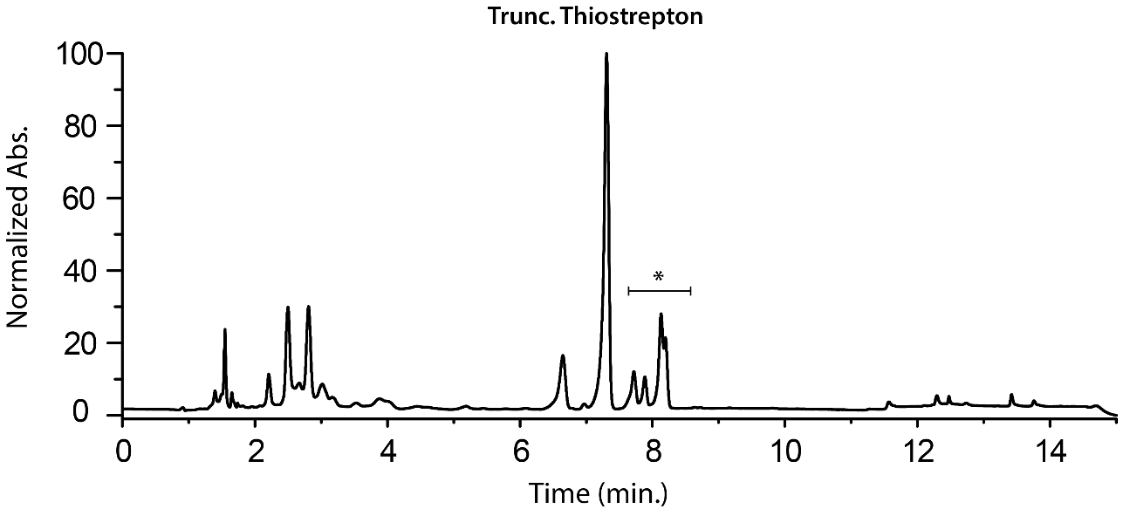

Full LC-MS UV chromatogram (280 nm) of the crude reaction mixture (*single modification)

Overall conversion was determined using analytical RP-HPLC (solvent A: 0.1 % FA in ACN, solvent B: 0.1 % FA in ddH_2O, gradient 60 % B to 10 % B over 40 minutes) of the crude mixture, showing a 41 % total conversion to single modified truncated thiostrepton:

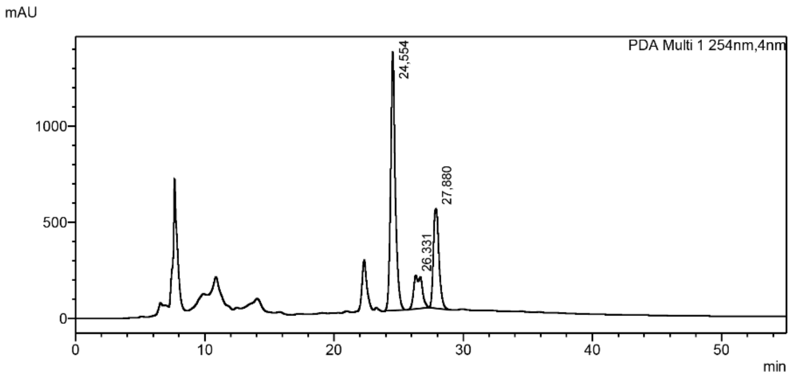

Peak #	Ret. Time (min.)	Area %
**1**	24.5	59.1
**2**	26.3	13.5
**3**	27.9	27.4

Preparative scale and purification: 25 mg (16 µmol) truncated thiostrepton (**3**) was dissolved in 5 mL TFE and 5 mL ddH_2O was added. 252 µL (3 mmol) freshly distilled cyclopentadiene was added daily for up to 7 days of total reaction time, during which the mixture was stirred at room temperature. Then, the mixture was transferred to a separatory funnel, and the bottom aqueous layer was separated. The aqueous layer was filtered over a plug of Celite and finally filtered over a microfilter (0.2 µm). The obtained clear filtrate was directly subjected to preparative HPLC.

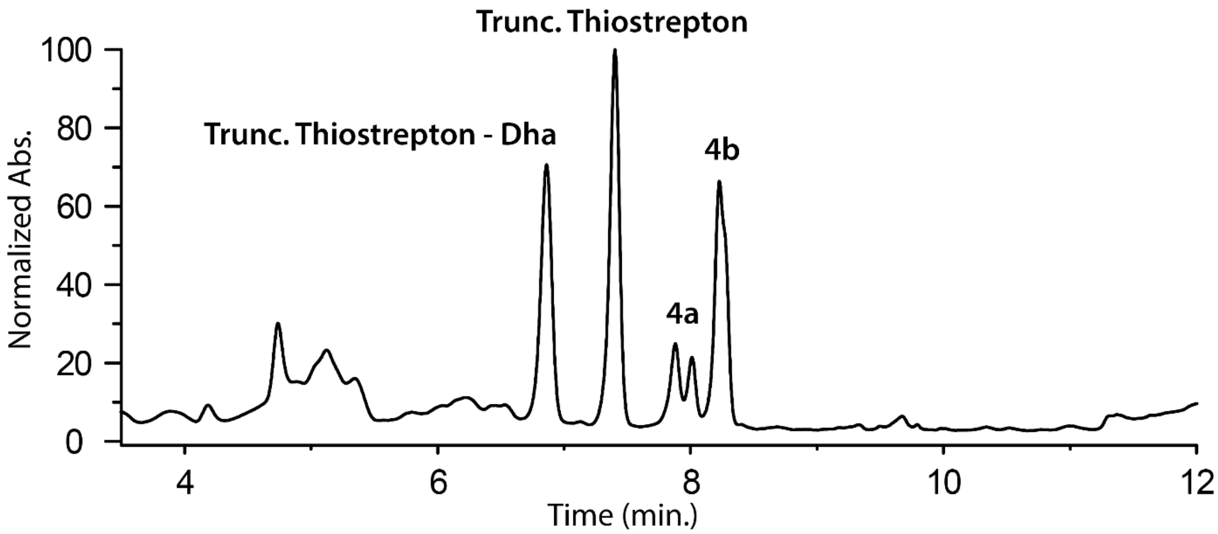

Zoom (3.5-12 min.) of LC-MS UV chromatogram (280 nm) of crude prep. scale reaction showing the 2 major singly modified products.

The crude product was purified by prep RP-HPLC (solvent A: 0.1 % FA in ACN, solvent B: 0.1 % FA in ddH_2O, gradient 60 % B to 10 % B over 40 minutes). Analysis of the fractions by LC-MS, followed by lyophilization of the combined pure fractions resulted in isolation of the pure Dha16-*endo* (**4a**) and Dha16-*exo* (**4b**) products as white brittle solids, which were each identified using HRMS.

Prep HPLC fraction collect result:

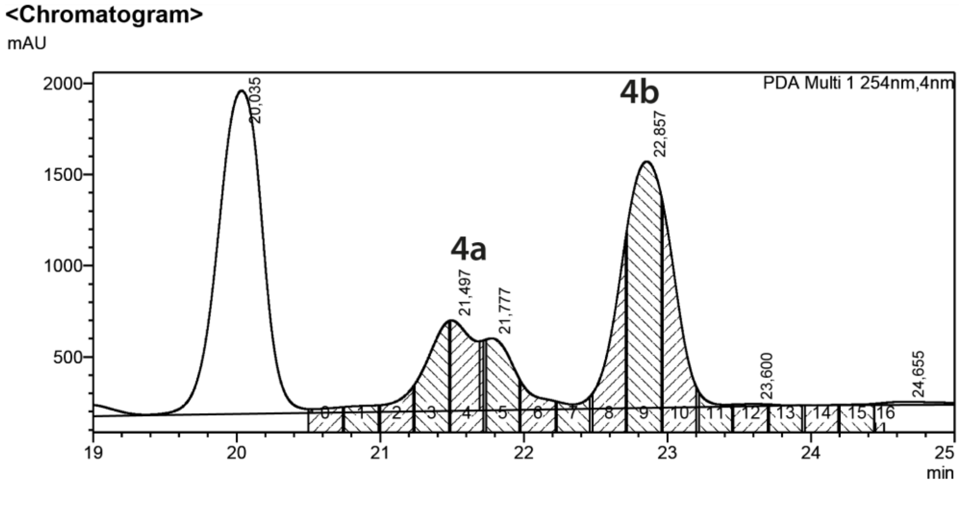

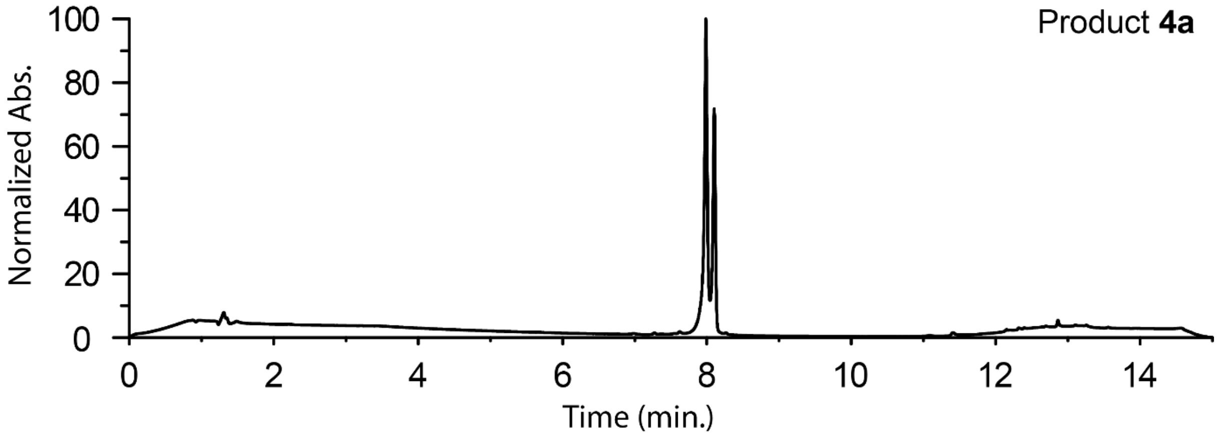

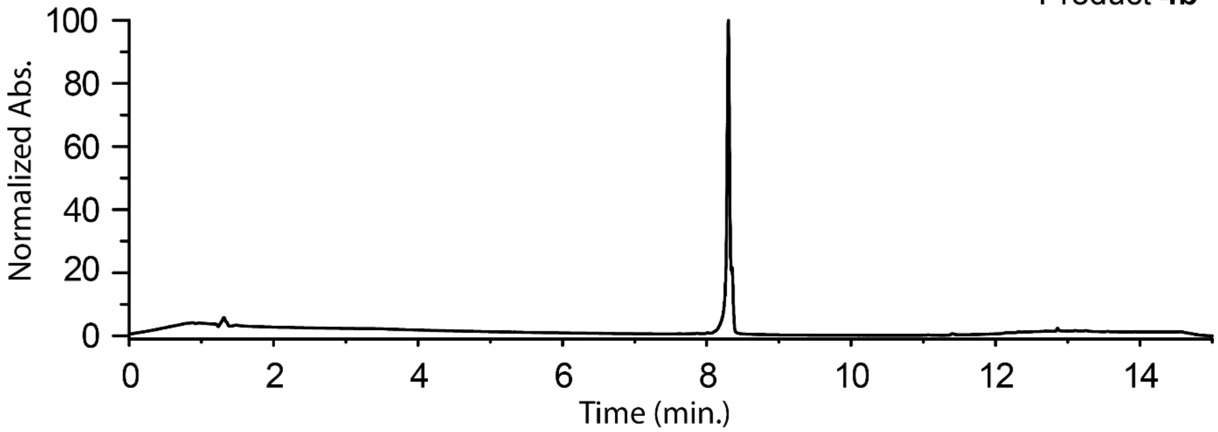

Compound	M_{calc} [M+H]$^+$	M_{found} [M+H]$^+$
**4a**	1661.526	1661.537
**4b**	1661.526	1661.535

Full LC-MS UV chromatograms (280 nm) and exact mass results of purified products 4a and 4b.

NMR analysis and identification of products 4a and 4b: Lyophilized samples of purified compounds **4a** and **4b** (approx. 1 mg) were dissolved in 500 µL $CDCl_3$. ^1H NMR, ^1H COSY NMR and ^1H-^{13}C HSQC NMR were used to identify the different site- and endo/exo isomers.

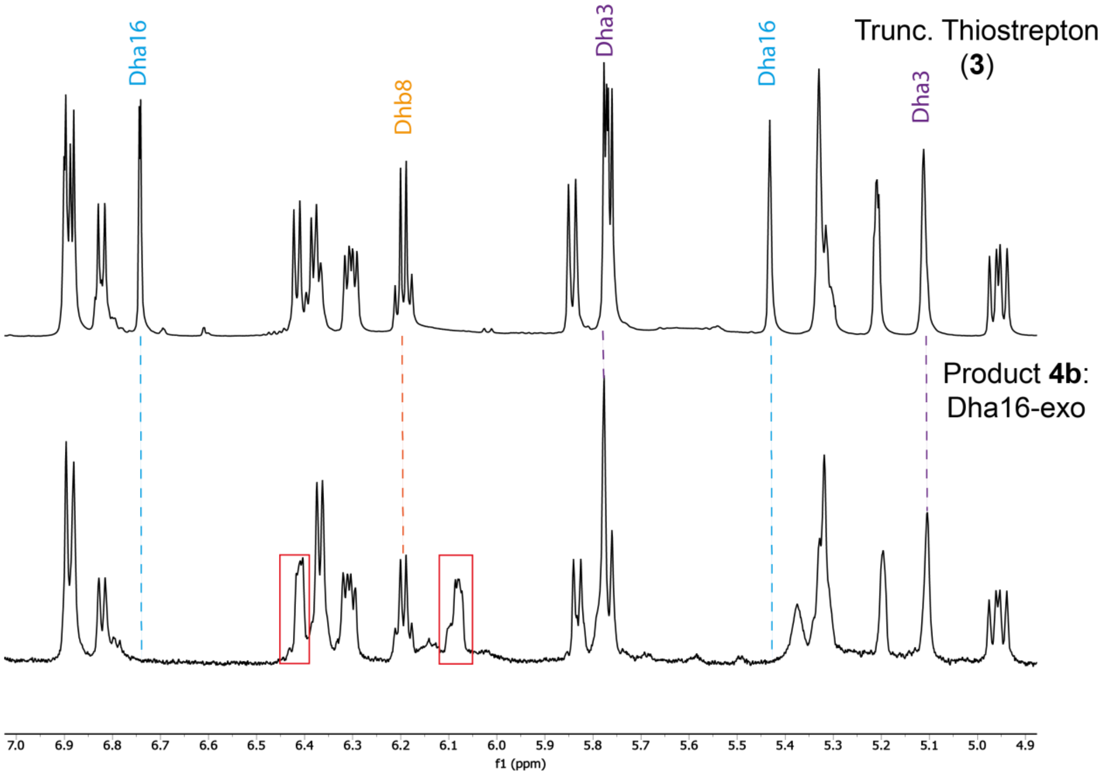

Overlay of ^{1}H NMR spectra of truncated thiostrepton (3) and compound 4b (Dha16-*exo*). When comparing the NMR spectra of truncated thiostrepton (**3**) and product **4b**, it can be seen that the methylene signals of Dha3 (purple) and Dhb8 (yellow) are conserved in compound **4b**. The set of signals originating from the tail methylene of Dha16 (blue) has disappeared, indicating that the reaction has taken place on Dha16. Moreover, the appearance of 2 doublet of doublets (red) is characteristic for the formation of the double bond in norbornene.

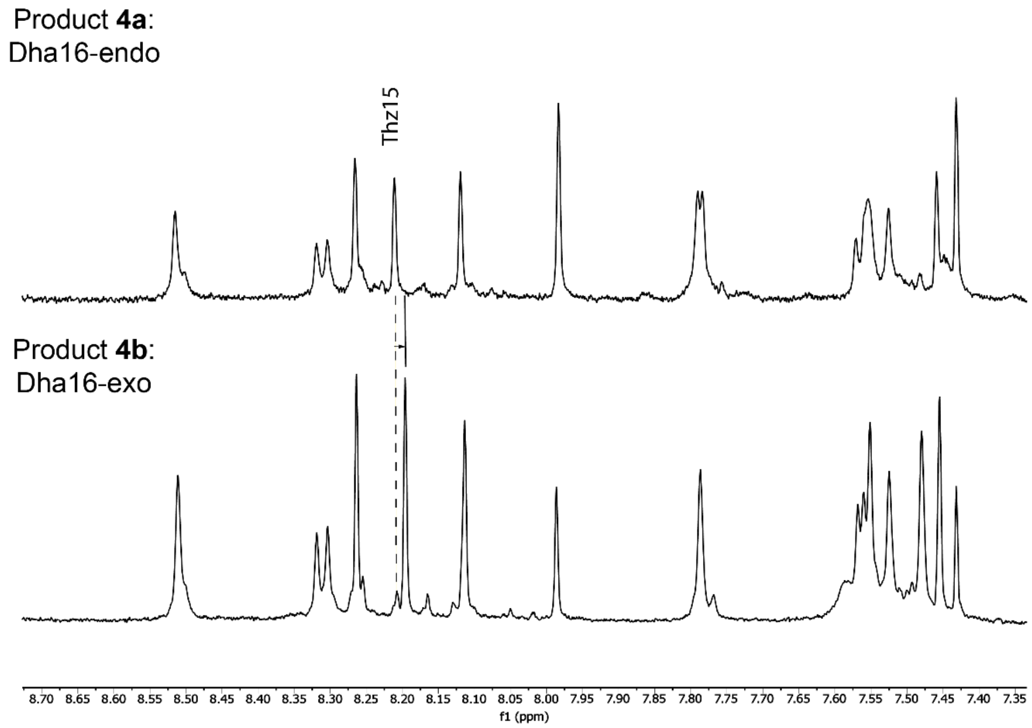

Overlay of ^{1}H NMR spectra of products 4a (Dha16-*endo*) and 4b (Dha16-*exo*). When comparing the NMR spectra of compound **4a** and compound **4b**, the same differences in characteristic signals can be observed as for products **2a** and **2b** (**SI-11**). Additionally, it can be seen that the C-H signal of thiazole 15 appears at a slightly higher

chemical shift in compound **4a** than in compound **4b**. This is due to lower shielding by the norbornene double bond in **4a**, which is pointing away from thiazole 15 in **4a**.

Diels-Alder reaction on nosiheptide

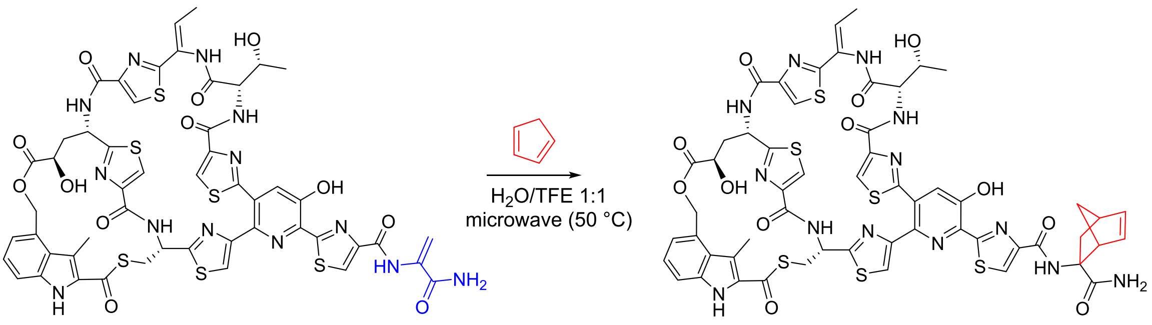

Nosiheptide

1.2 mg (1 µmol) nosiheptide was dissolved in 0.5 mL TFE in a microwave vial. Then, 0.5 mL ddH_2O and 50 µL (0.6 mmol) freshly distilled cyclopentadiene were added and the mixture was heated for 16 hours in a microwave reactor at 50 °C (50 W power). After addition of another portion (50 µL) of freshly distilled cyclopentadiene the mixture was heated again in a microwave reactor at 50 °C for 16 hours. For LC-MS and HPLC analysis, samples were prepared by diluting 100 µL reaction mixture with 200 µL ddH_2O/ACN 1:1 and filtering over a microfilter (0.2 µm).

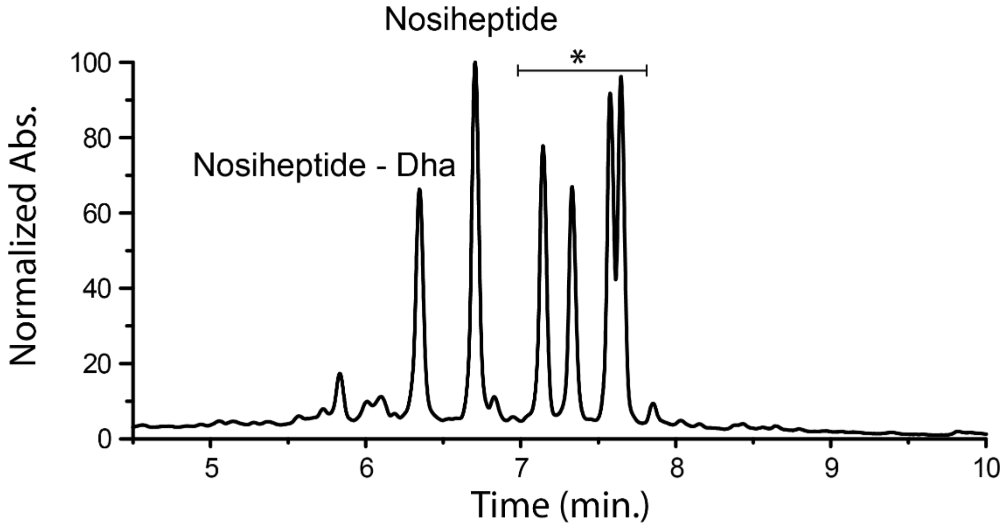

Zoom of LC-MS UV chromatogram (280 nm) of the crude reaction mixture (*single modification)

Overall conversion was determined using analytical RP-HPLC (solvent A: 0.1 % FA in ACN, solvent B: 0.1 % FA in ddH_2O, gradient 60 % B to 10 % B over 40 minutes) of the crude mixture, showing a total conversion of 75 % to single modified nosiheptide:

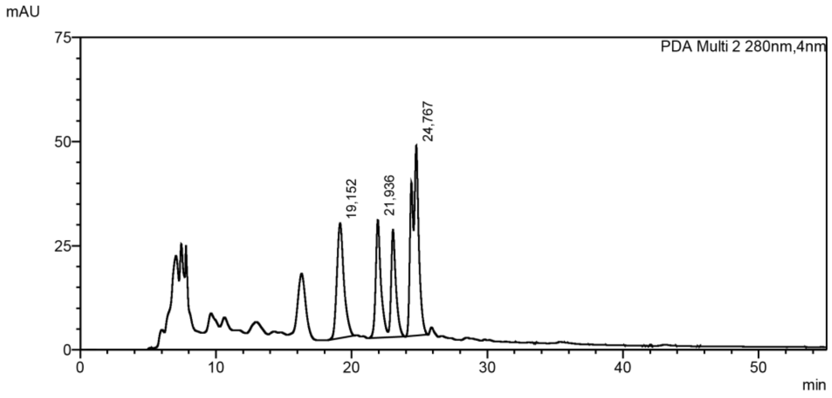

**Peak #**	**Ret. Time (min.)**	**Area %**
**1**	19.2	24.6
**2**	21.9	34.5
**3**	24.8	40.9

Diels-Alder reaction on nisin Z

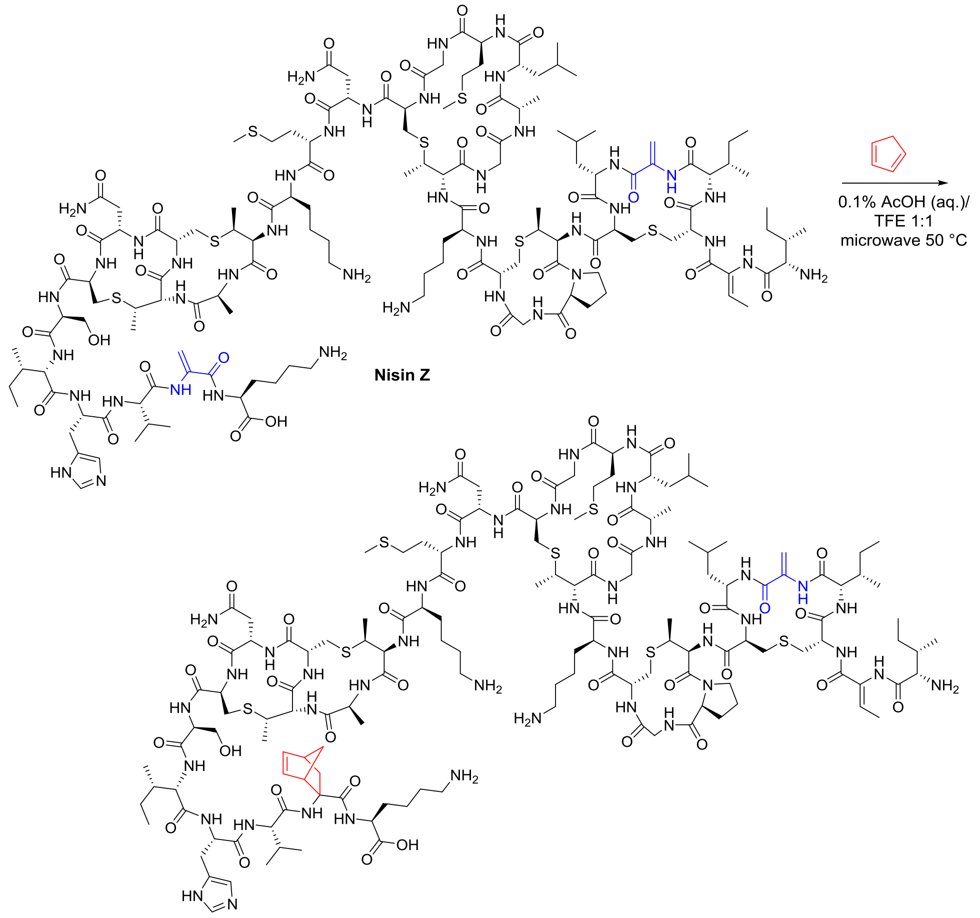

In a typical procedure, 3.3 mg (1 µmol) nisin Z was dissolved in 0.5 mL ddH_2O with 0.1 % AcOH. 0.5 mL TFE was added, followed by 100 µL (1.2 mmol) freshly distilled cyclopentadiene. The mixture was stirred overnight in a microwave reactor at 50 °C (50 W power). For LC-MS analysis, samples were prepared by diluting 100 µL reaction mixture with 200 µL ddH_2O/ACN 1:1 and filtering over a microfilter (0.2 µm).

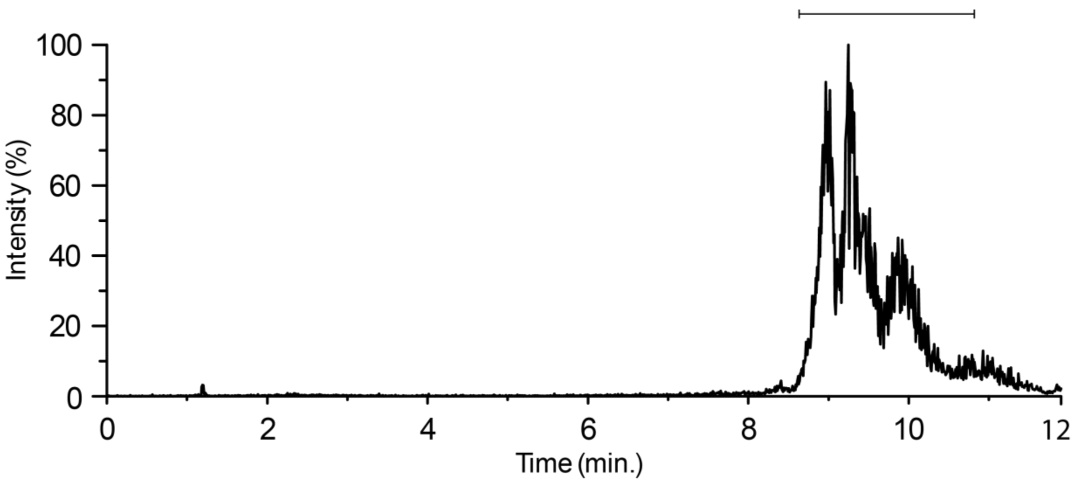

TIC chromatogram of the crude reaction mixture. The area indicated with the black line was combined and the resulting mass spectrum used for deconvolution (see next page).

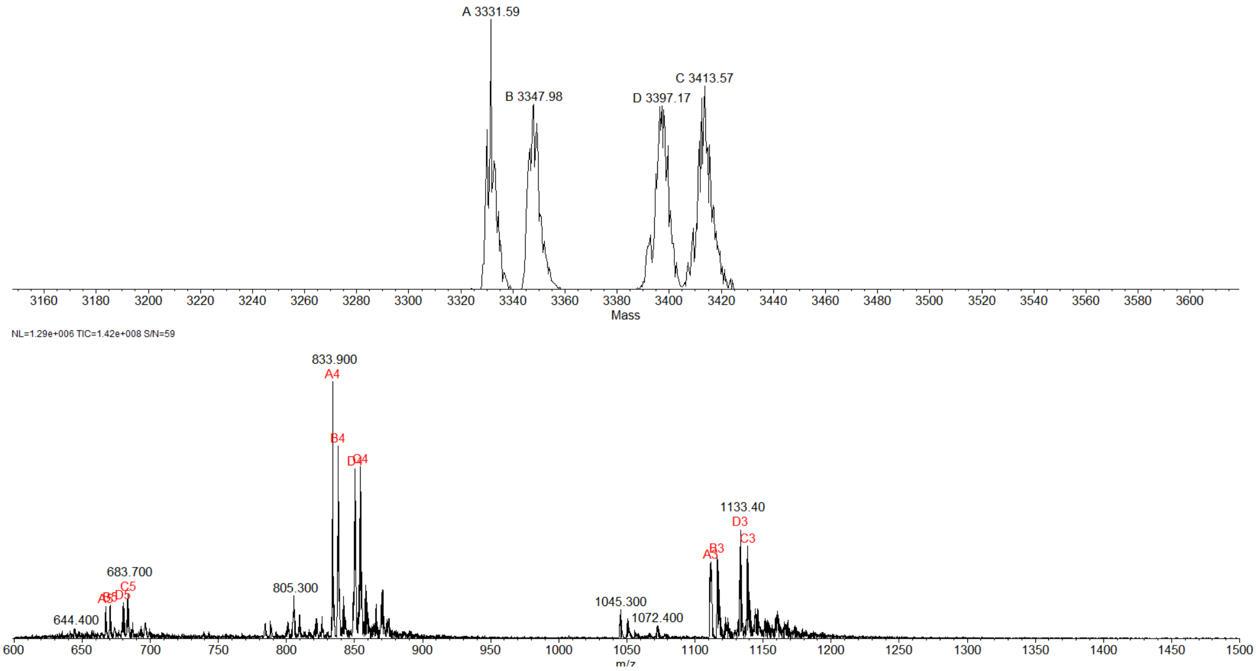

Mass spectrum of crude mixture (bottom) and deconvolution result (top).

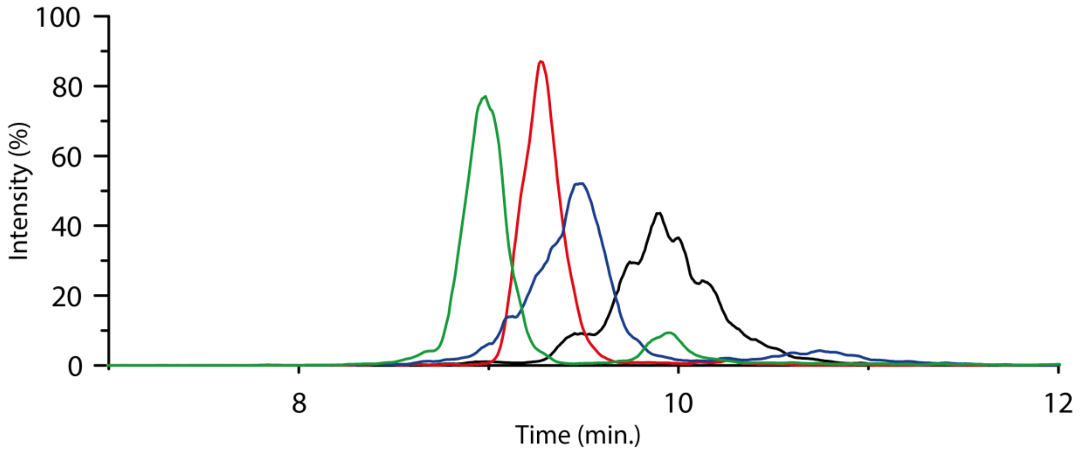

Species	$[M+4H]^{4+}_{found}$	M_{calc}	M_{decon}	Area %
Nisin Z	833.9	3331	3332	24
Nisin Z + H_2O	838.0	3349	3348	24
Nisin Z + 1 Mod.	850.5	3397	3397	27
Nisin Z + H_2O + 1 Mod.	854.4	3415	3414	25

Zoom in of extracted ion chromatograms (top) of $[M+4H]^{4+}$ species of nisin Z (red), Nisin Z + H_2O (green), nisin Z + 1 mod. + H_2O (blue) and nisin Z + 1 Mod. (black). Table showing the deconvolution results of the different species and their relative area percentages (bottom). Due to the low UV absorption coefficient of nisin Z, the conversion could not be determined using UV signal integration, but was instead estimated using ion current integrations, assuming that all nisin derivatives are ionized to a similar extent. The extracted ion chromatogram signals above were integrated individually and compared to obtain the relative composition of the mixture in order to give an estimate of the total conversion to Diels-Alder modified nisin Z. From the composition percentages a total conversion of 52 % to Diels-Alder modified nisin Z can be estimated (including modified nisin Z that also underwent water addition).

Biological Activity Assays

Preparation of antimicrobial agents

Thiostrepton and its variants were dissolved and diluted in DMSO to a concentration of 640 µg/ml, and stored at -20 °C. Before use, they were diluted 20 fold in Mueller Hinton Broth 2 (CAMHB; cation-adjusted, Sigma-Aldrich). Dilution of the thiostrepton compounds in CAMHB was done in 50 % volume steps to prevent precipitation. Vancomycin for the quality controls was dissolved in MQ to a concentration of 256 mg/ml and stored at -20 °C. It was diluted in CAMHB to a final concentration of 256 µg/ml before use.

Strains and growth conditions

The MIC values of thiostrepton and its variants were determined for *Staphylococcus aureus* LMG 10147 (ATCC29213) and *Enterococcus faecalis* LMG 08222 (ATCC29212). LMG 10147 and LMG 08222 were cultured from glycerol stocks on LB (Formedium™) and GM17 (Difco™) plates respectively. For the MIC determination tests, LMG 10147 was grown in CAMHB, while LMG 08222 was grown in CAMHB + 3 % v/v lysed horse blood (tcs biosciences). For all steps, the incubation temperature was 37 °C.

DMSO as a solvent

As the thiostrepton compounds were diluted from stocks in DMSO, residual amounts of DMSO remained in the MIC test plates (down from 2,5% in the first well). As a control for potential side effects of DMSO, both strains were grown in DMSO concentrations representative of those present in the test plates, without the addition of antimicrobial compounds. No growth inhibition was observed at any of the tested DMSO concentrations.

Broth microdilution

MIC testing and internal controls were performed employing the 96-well plate broth microdilution method described in Wiegand et al., 2008[6], which is outlined in short here.

First, 50 µl and 100 µl of sterile CAMHB is added to columns 2-11 and 12 respectively, of a 96-well plate. Then, 100 µl of freshly diluted test compound is added to the first well. A serial dilution of the compound is achieved by transferring 50 µl from the first well to the second, mixing, and then continuing these steps until well 10. Finally, 50 µl of bacterial suspension is added to wells 1 to 11, resulting in a final cfu of 5 x 105 ml^{-1} in each well. Well 11, lacking the test compound, functions as a growth control and well 12 as a sterility control. Before incubation, several dilutions from a growth control well are plated as a control for the number of cfu's. To ensure MIC data reliability, MIC values for vancomycin were determined for every series of tests performed, as described by the CLSI standard.[7]

The MIC test plates were placed in an airtight container to prevent evaporation, and incubated for 20 hours before reading. The concentration of compound in the first well of the serial dilution that shows no visible growth of the test strain, is considered the MIC value. All compounds were tested three times in triplicates.

Antibiotic	MIC (µg/mL) against *S. aureus* (MSSA)	MIC (µg/mL) against *E. faecalis* (VSE)
**Vancomycin**	1	4
**Thiostrepton**	0.5	0.5
**2a**	2	2
**2b**	2	2
**2c**	2	1
**3**	0.5	1
**4a**	4	2
**4b**	2	2

Tetrazine Ligation of thiostrepton Diels-Alder Adducts

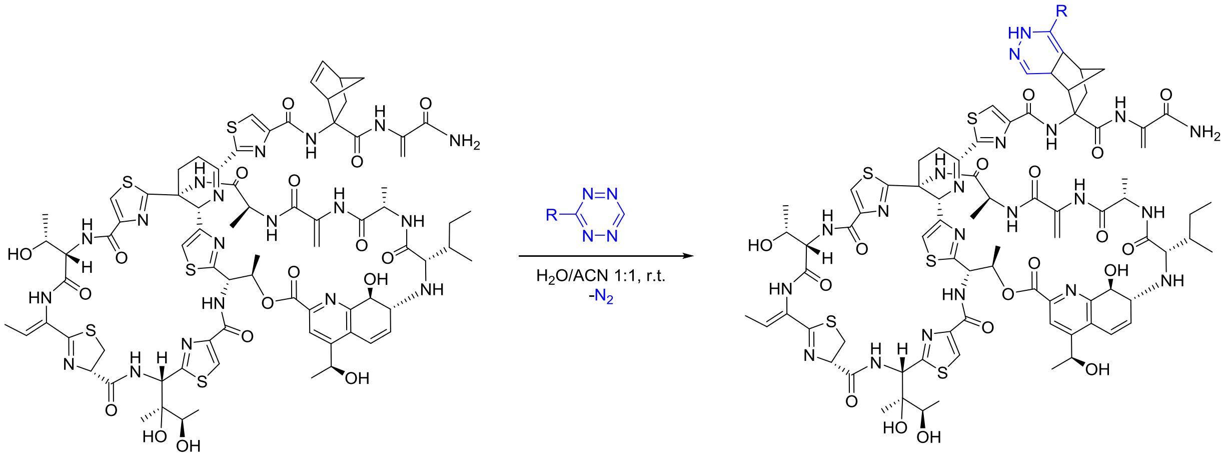

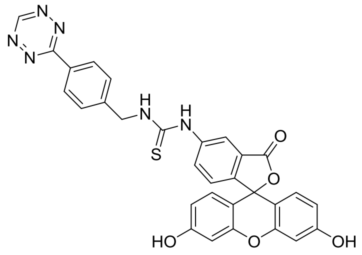

Fluorescein-tetrazine (**9**)

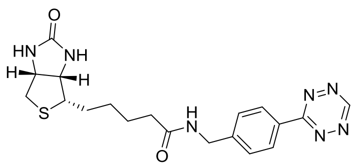

Biotin-tetrazine (**10**)

To 44 µL 20 µM Dha16-*exo* Diels-Alder modified thiostrepton in ddH_2O/ACN 1:1 was added 2 µL 46 mM tetrazine in DMSO. The mixture was stirred overnight at room temperature, after which the reaction mixture was analyzed by MALDI-TOF MS (CHCA-matrix).

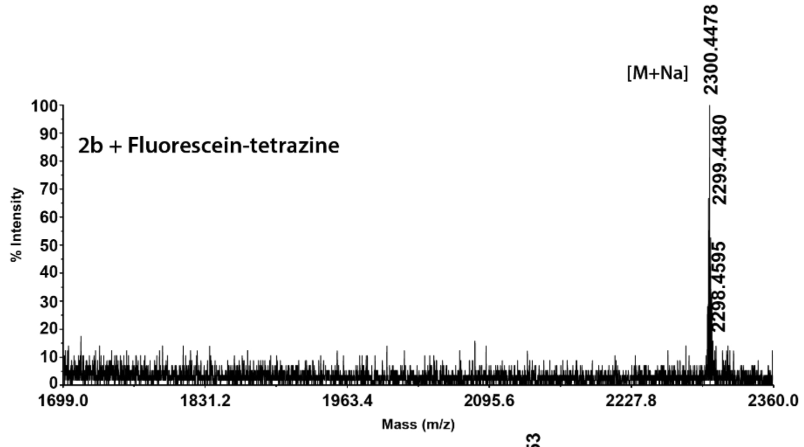

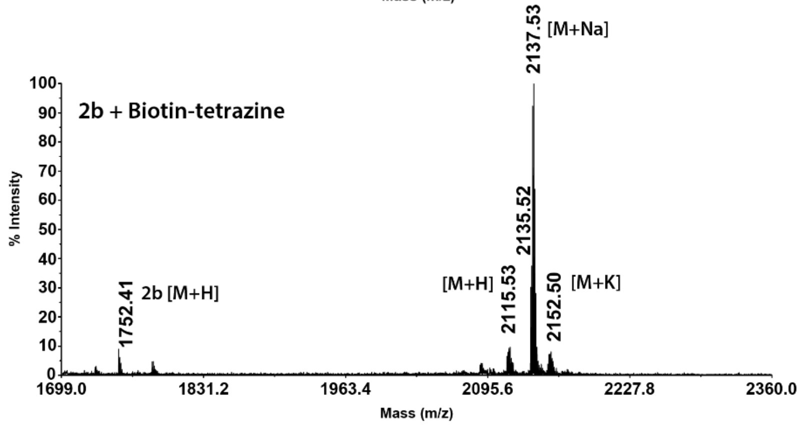

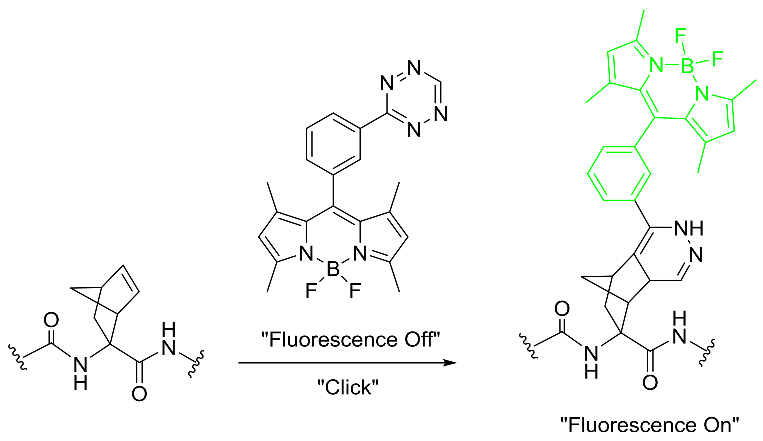

Fluorescence turn-on upon BODIPY-Tz click: 3 µL 2.16 mM Dha16-*endo* Diels-Alder modified thiostrepton in DMSO and 3 µL 51 µM BODIPY-Tz in H_2O/ACN 1:1 (used immediately after preparative HPLC purification) were added to 50 µL ACN (final concentrations: 116 µM modified thiostrepton and 2.7 µM BODIPY-Tz). Under UV light (365 nm) a clear fluorescence was observed after stirring at r.t. for 1 hour. After overnight reaction, the mixture was analyzed by MALDI-TOF MS (CHCA matrix). For fluorescence measurements, the reaction mixture and control were both diluted to 2 mL with 1944 µL ACN.

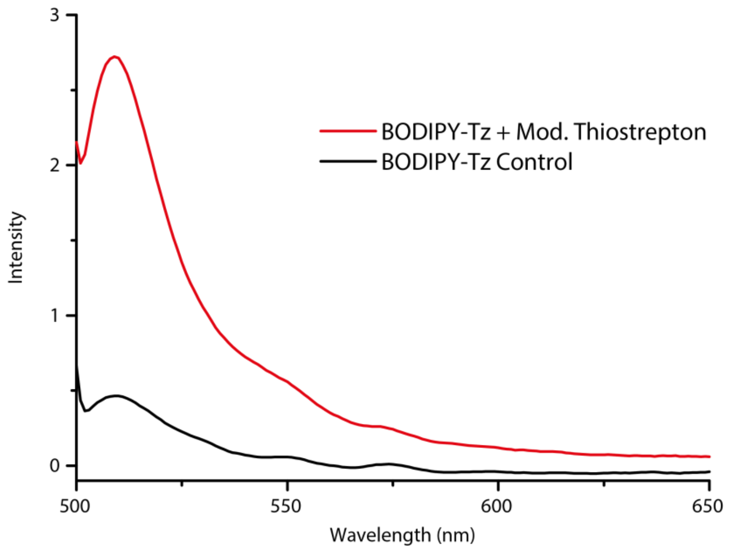

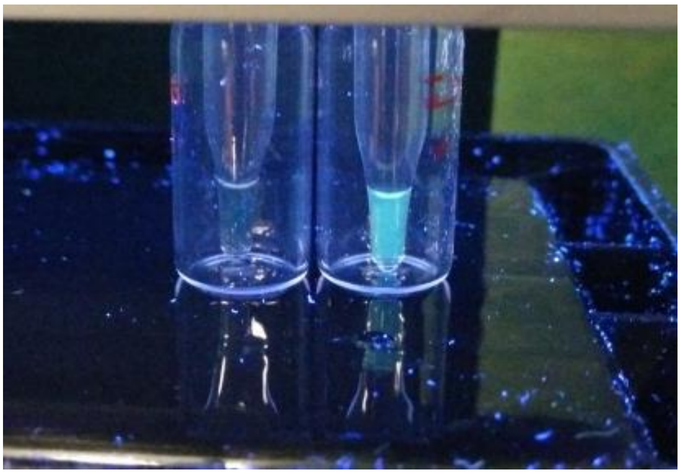

Left: fluorimetric measurements of BODIPY-Tz click (red) compared to DMSO control (black). Right: visible fluorescence turn-on in undiluted reaction (left is control, right is reaction) under UV light (365 nm).

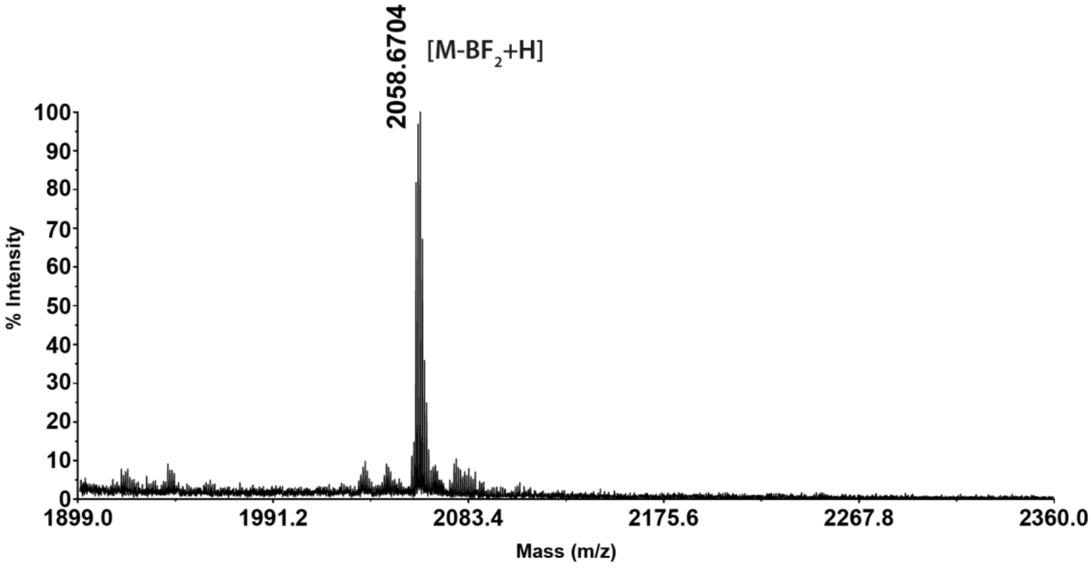

NMR Spectra

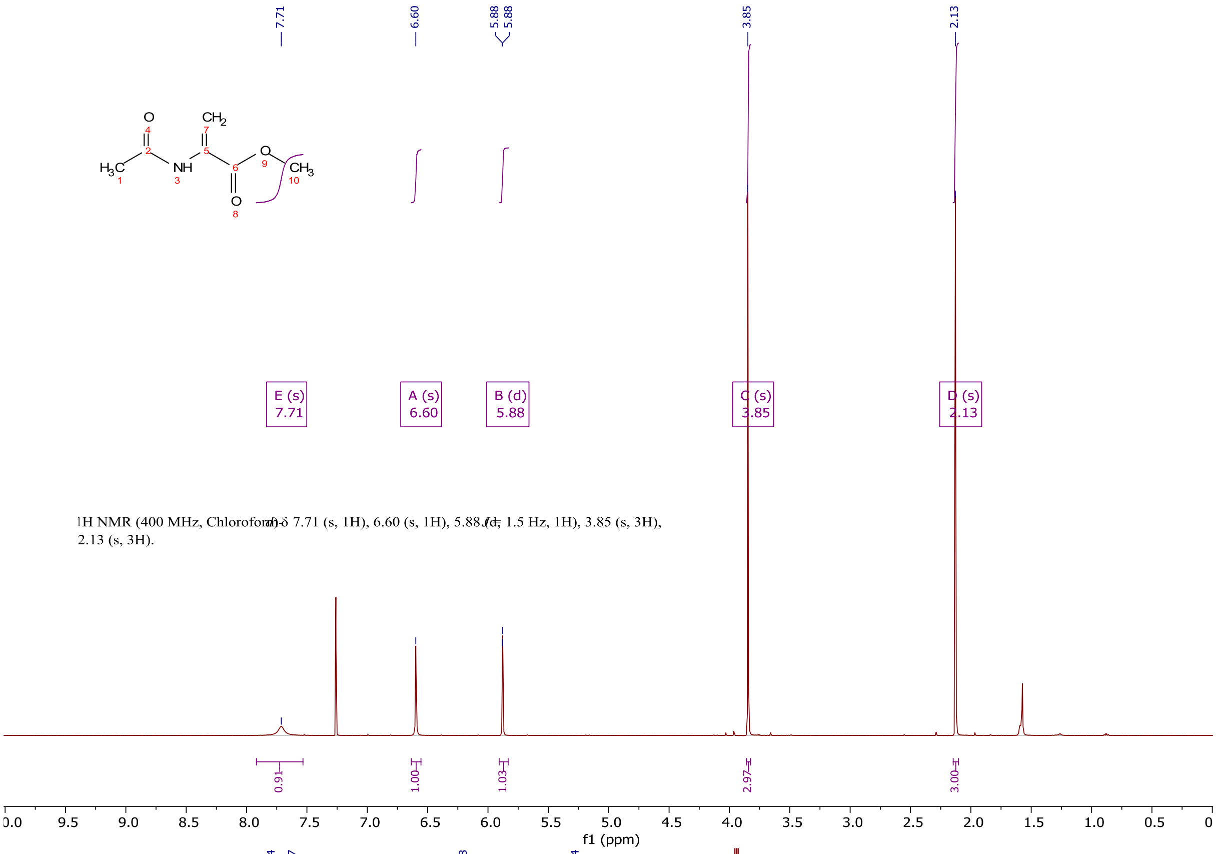
1H NMR (400 MHz, Chloroform-d) δ 7.71 (s, 1H), 6.60 (s, 1H), 5.88 (d, J = 1.5 Hz, 1H), 3.85 (s, 3H), 2.13 (s, 3H).
E (s) 7.71
A (s) 6.60
B (d) 5.88
C (s) 3.85
D (s) 2.13
f1 (ppm)

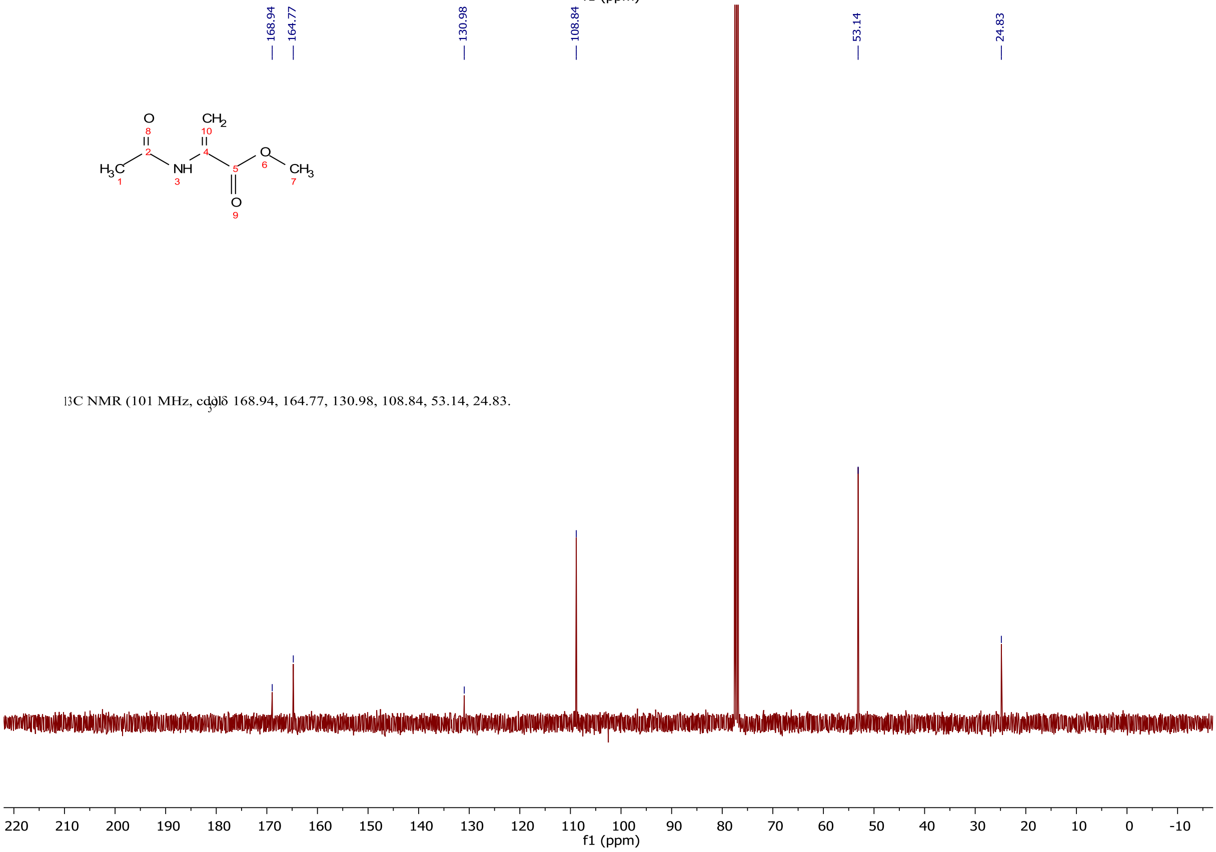
13C NMR (101 MHz, cdcl3) δ 168.94, 164.77, 130.98, 108.84, 53.14, 24.83.
f1 (ppm)

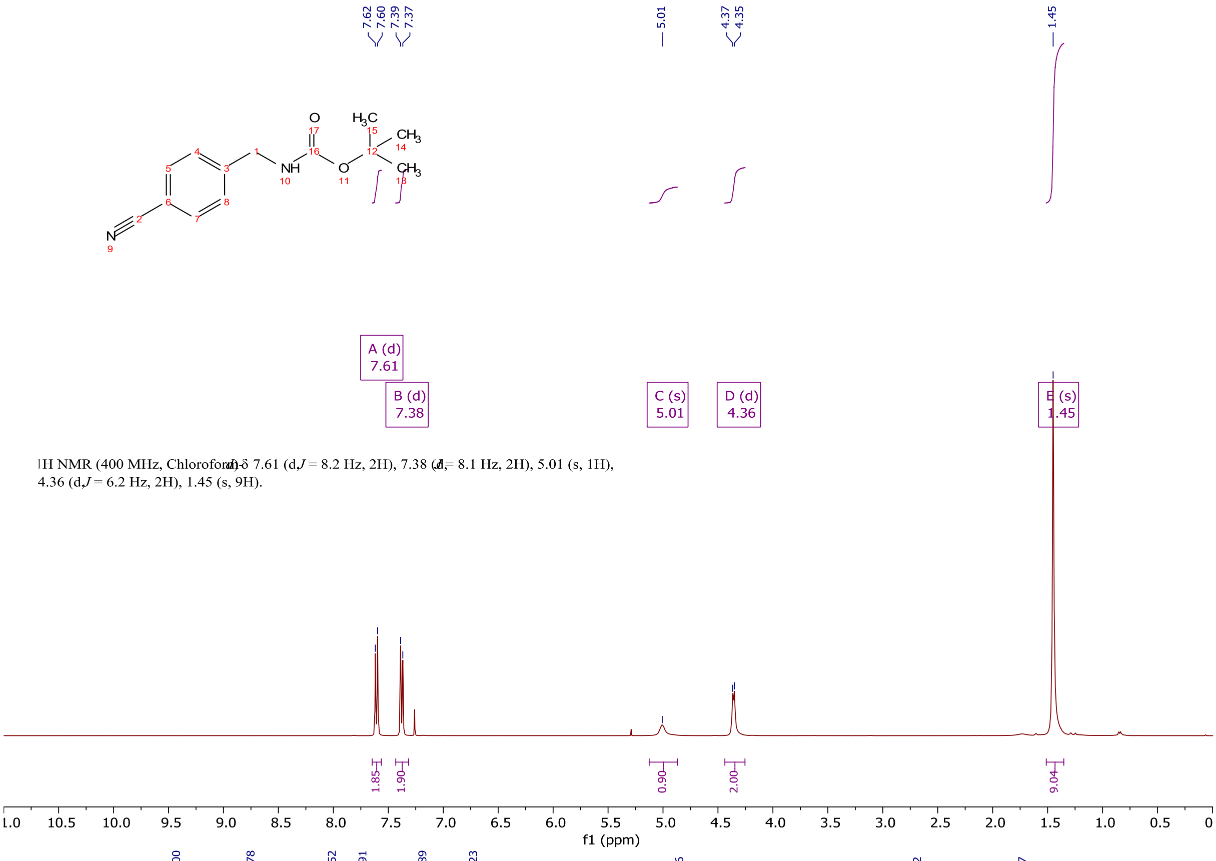

^{1}H NMR (400 MHz, Chloroform-*d*) δ 7.61 (d, *J* = 8.2 Hz, 2H), 7.38 (d, *J* = 8.1 Hz, 2H), 5.01 (s, 1H), 4.36 (d, *J* = 6.2 Hz, 2H), 1.45 (s, 9H).

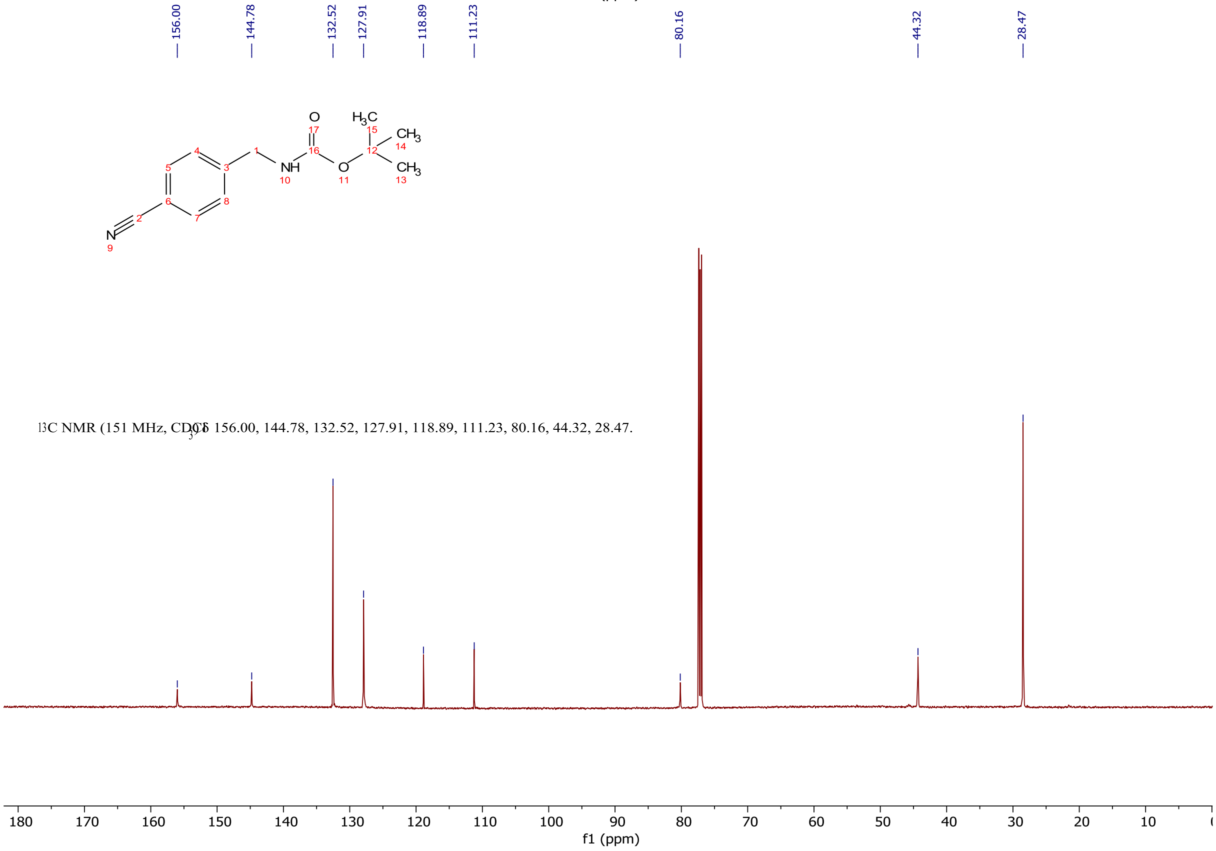

^{13}C NMR (151 MHz, $CDCl_3$) δ 156.00, 144.78, 132.52, 127.91, 118.89, 111.23, 80.16, 44.32, 28.47.

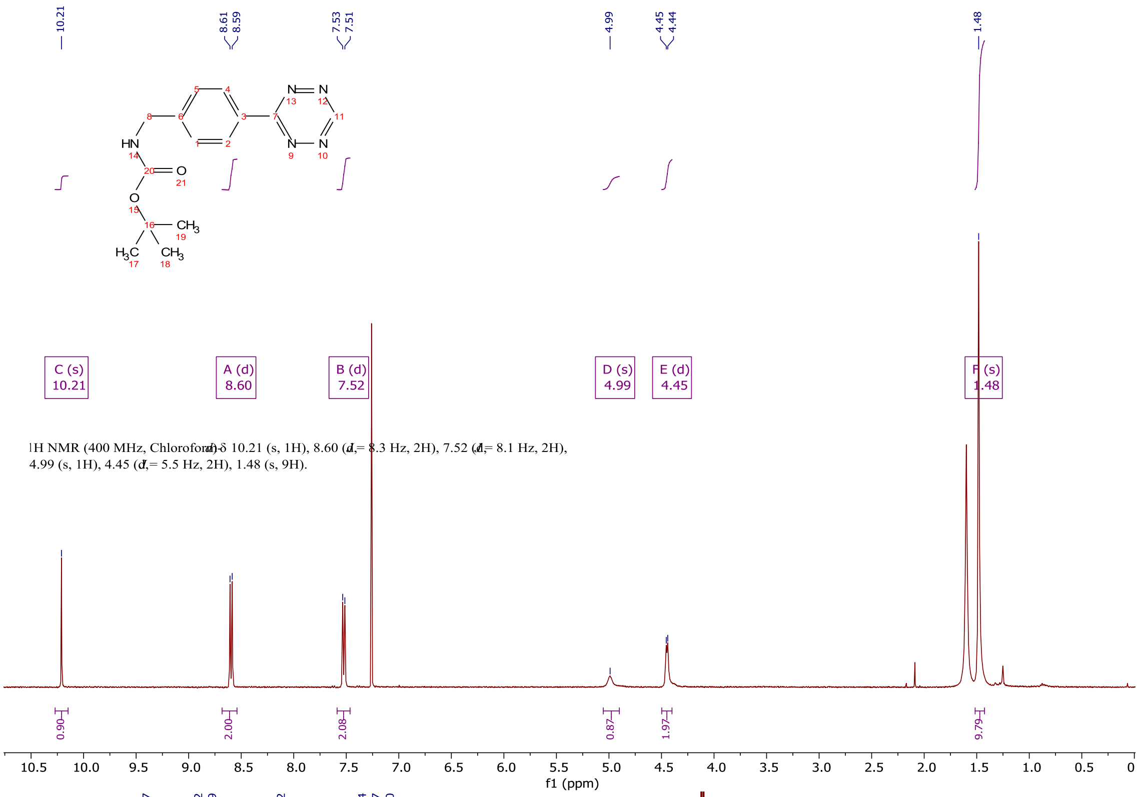

^{1}H NMR (400 MHz, Chloroform-*d*) δ 10.21 (s, 1H), 8.60 (d, *J* = 8.3 Hz, 2H), 7.52 (d, *J* = 8.1 Hz, 2H), 4.99 (s, 1H), 4.45 (d, *J* = 5.5 Hz, 2H), 1.48 (s, 9H).

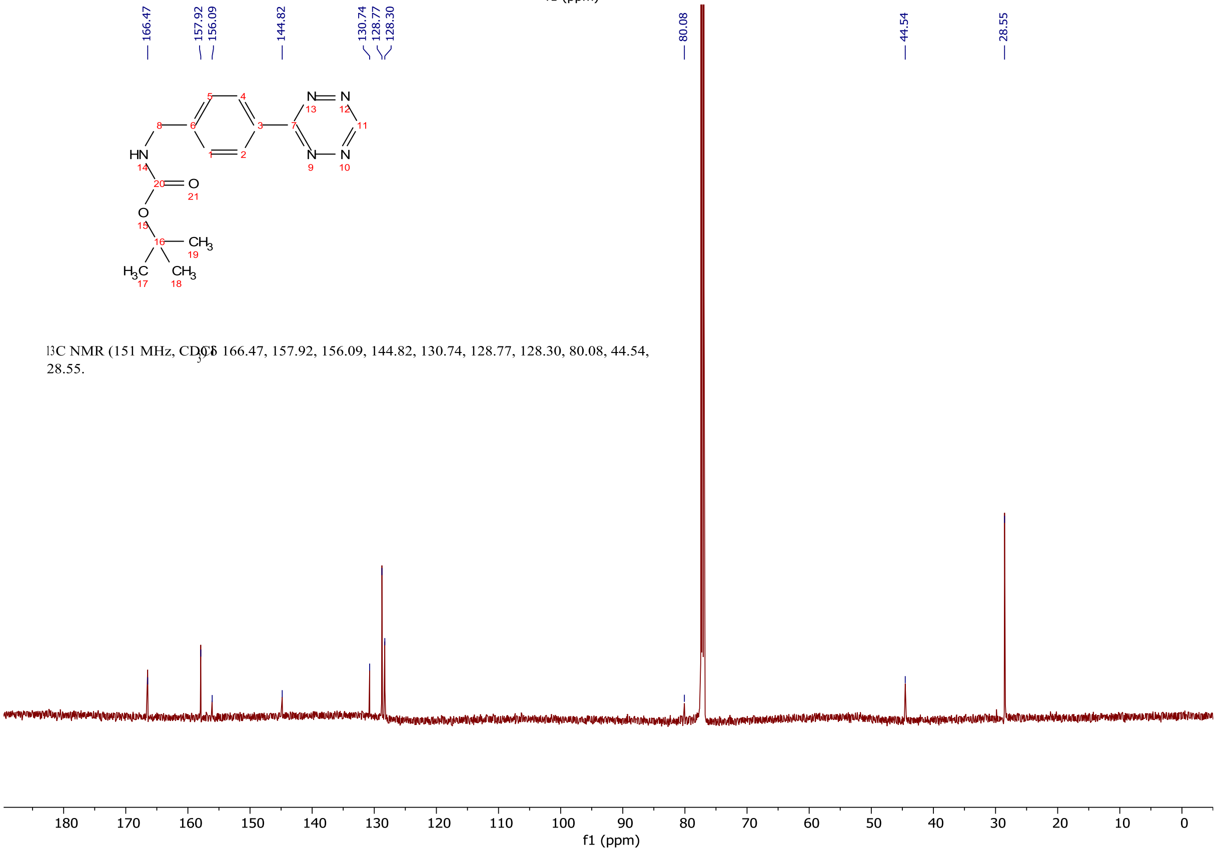

^{13}C NMR (151 MHz, $CDCl_3$) δ 166.47, 157.92, 156.09, 144.82, 130.74, 128.77, 128.30, 80.08, 44.54, 28.55.

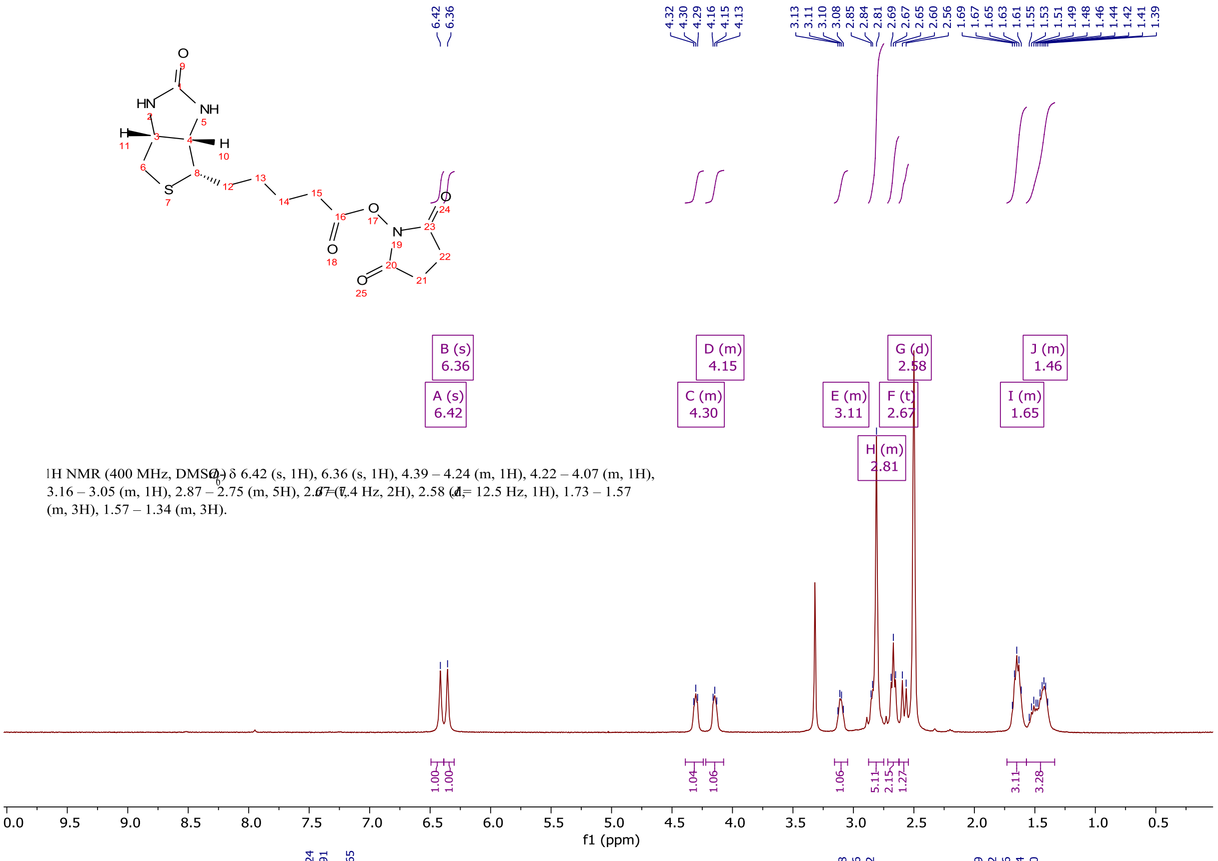

^{1}H NMR (400 MHz, DMSO-d_6) δ 6.42 (s, 1H), 6.36 (s, 1H), 4.39 – 4.24 (m, 1H), 4.22 – 4.07 (m, 1H), 3.16 – 3.05 (m, 1H), 2.87 – 2.75 (m, 5H), 2.67 (t, J = 7.4 Hz, 2H), 2.58 (d, J = 12.5 Hz, 1H), 1.73 – 1.57 (m, 3H), 1.57 – 1.34 (m, 3H).

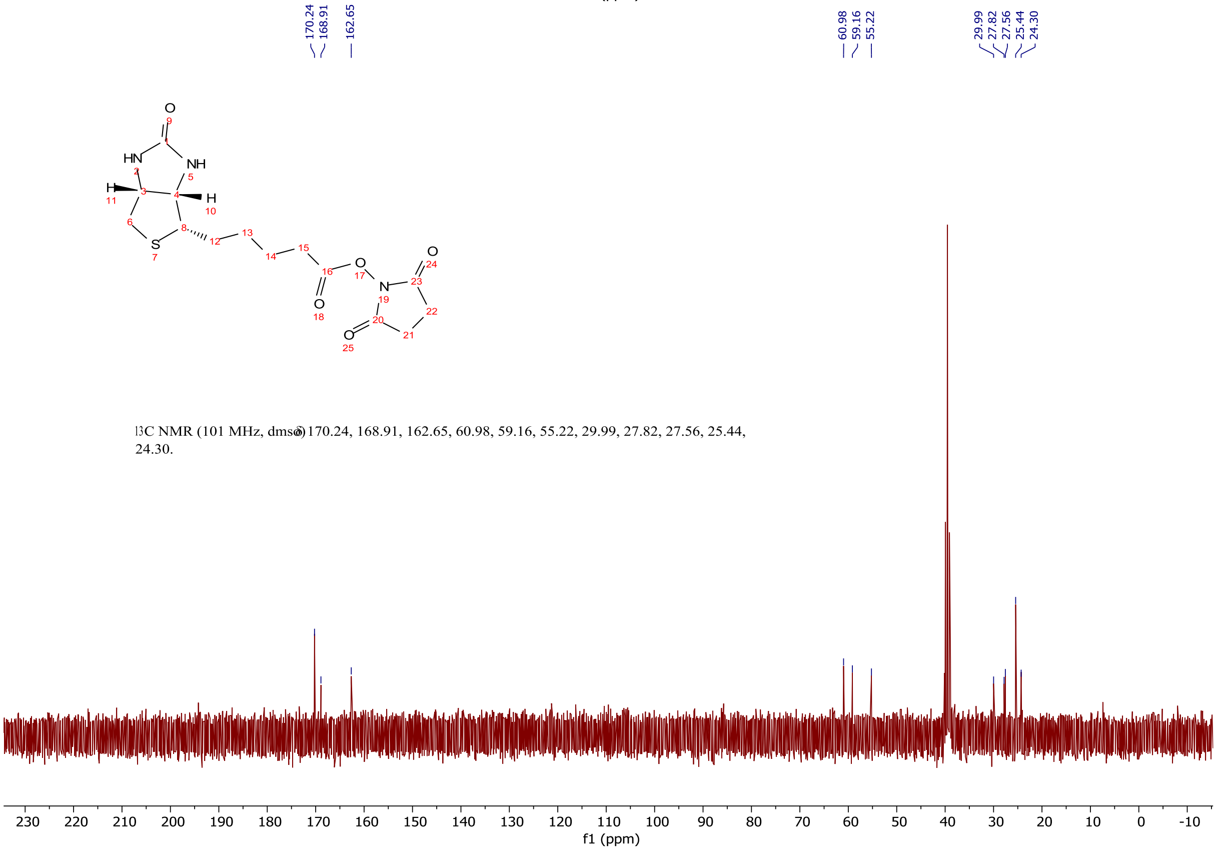

^{13}C NMR (101 MHz, dmso) δ 170.24, 168.91, 162.65, 60.98, 59.16, 55.22, 29.99, 27.82, 27.56, 25.44, 24.30.

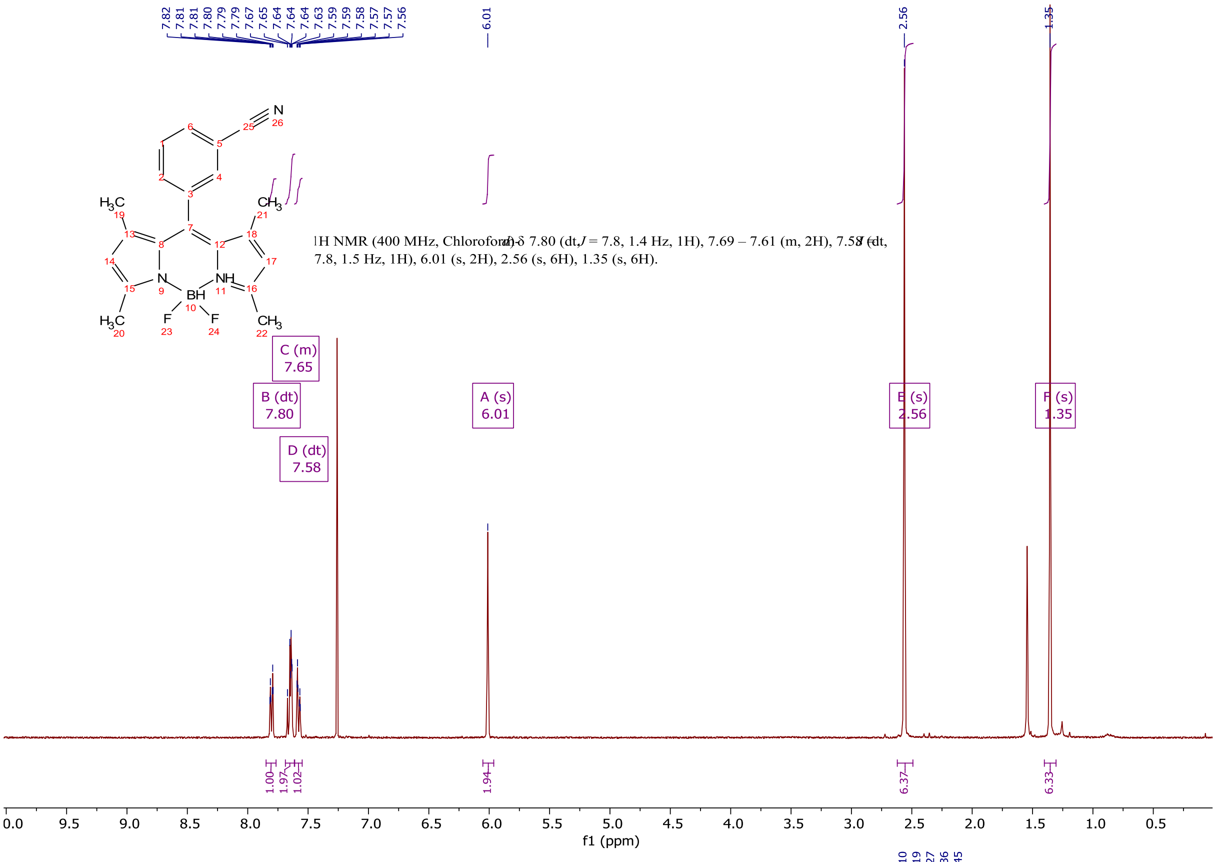
1H NMR (400 MHz, Chloroform-d) δ 7.80 (dt, J = 7.8, 1.4 Hz, 1H), 7.69 – 7.61 (m, 2H), 7.58 (dt, J = 7.8, 1.5 Hz, 1H), 6.01 (s, 2H), 2.56 (s, 6H), 1.35 (s, 6H).
C (m) 7.65
B (dt) 7.80
D (dt) 7.58
A (s) 6.01
E (s) 2.56
F (s) 1.35
f1 (ppm)

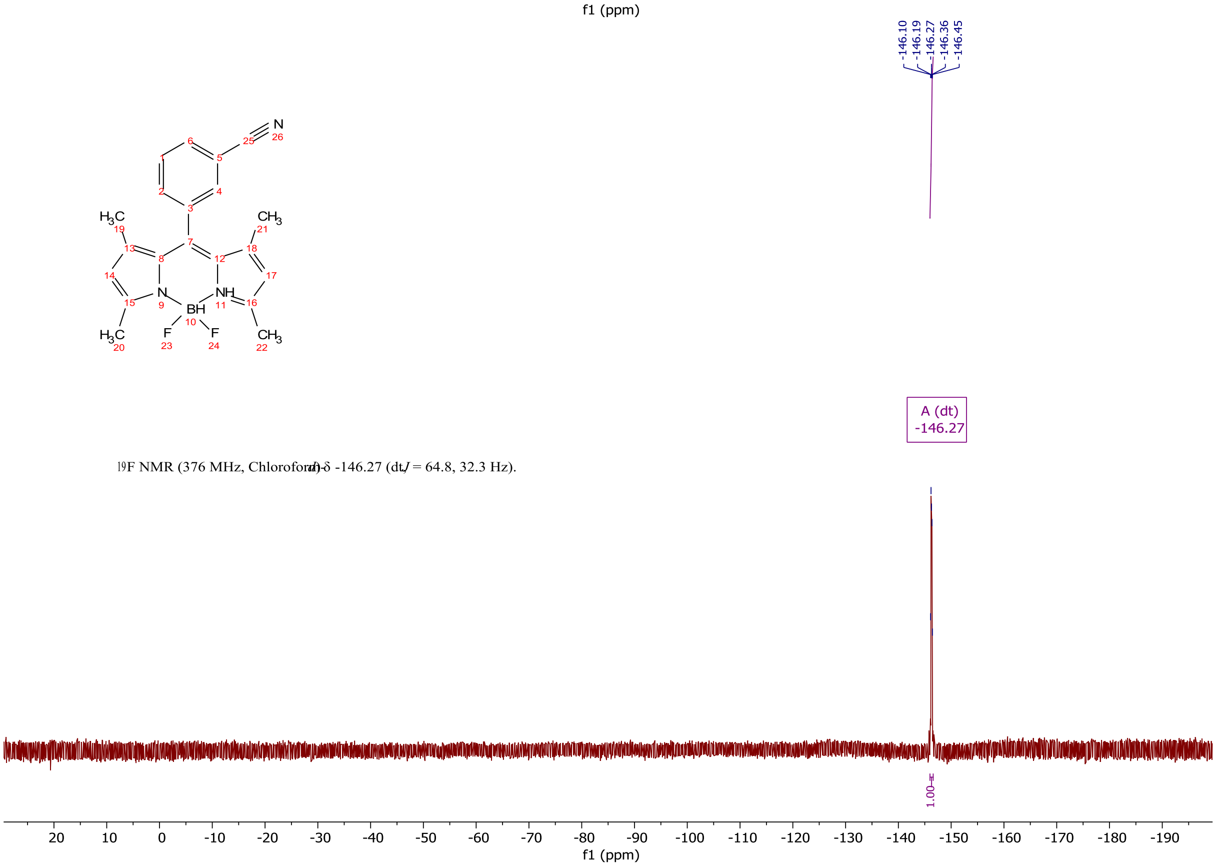
19F NMR (376 MHz, Chloroform-d) δ -146.27 (dt, J = 64.8, 32.3 Hz).
A (dt) -146.27
f1 (ppm)

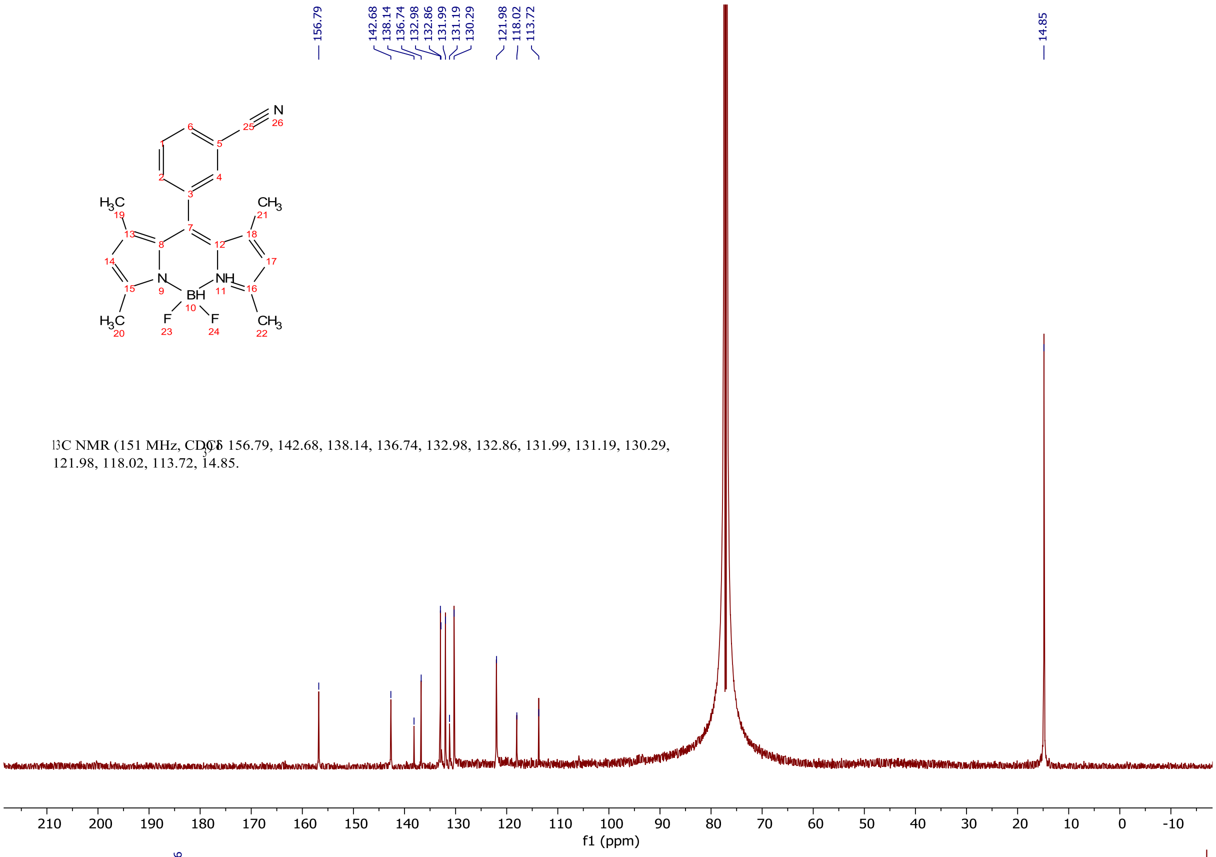
13C NMR (151 MHz, CDCl3) δ 156.79, 142.68, 138.14, 136.74, 132.98, 132.86, 131.99, 131.19, 130.29, 121.98, 118.02, 113.72, 14.85.
f1 (ppm)

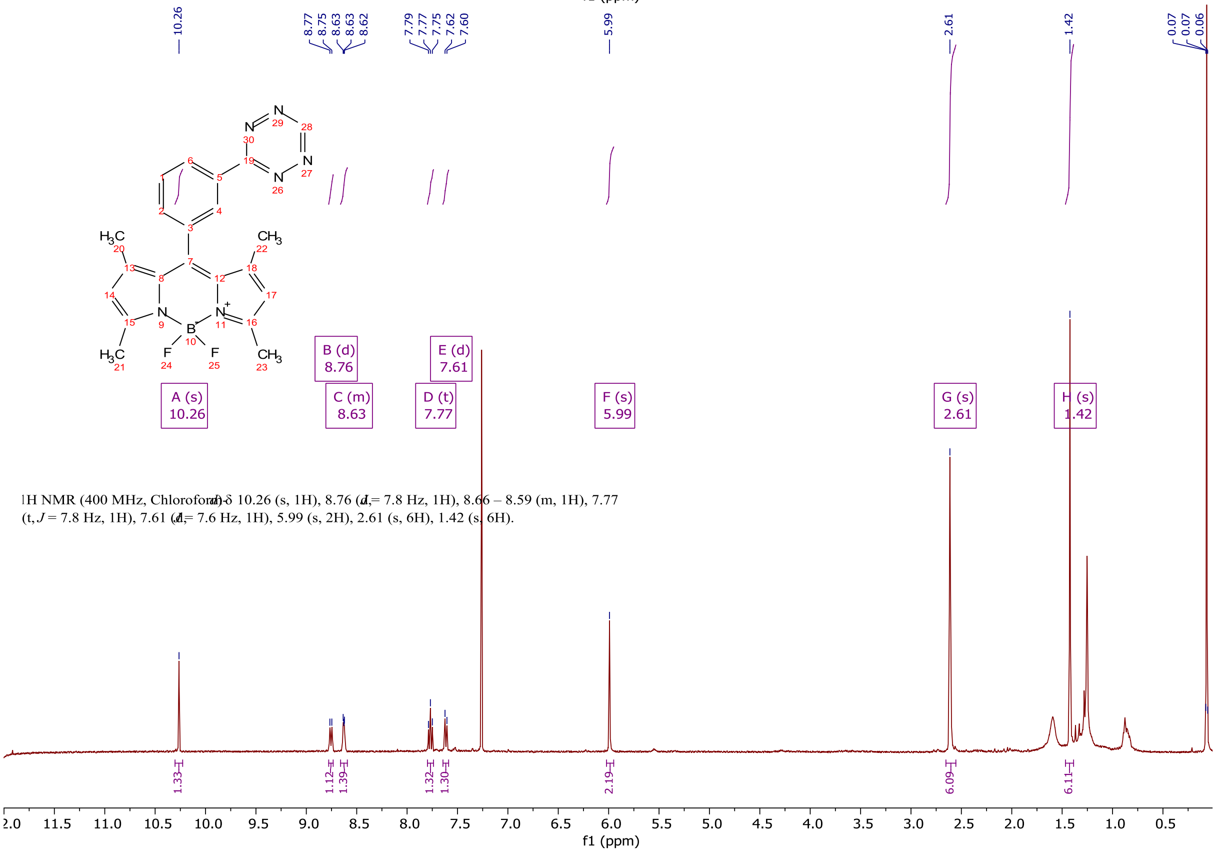
A (s) 10.26
B (d) 8.76
C (m) 8.63
D (t) 7.77
E (d) 7.61
F (s) 5.99
G (s) 2.61
H (s) 1.42
1H NMR (400 MHz, Chloroform-d) δ 10.26 (s, 1H), 8.76 (d, J = 7.8 Hz, 1H), 8.66 – 8.59 (m, 1H), 7.77 (t, J = 7.8 Hz, 1H), 7.61 (d, J = 7.6 Hz, 1H), 5.99 (s, 2H), 2.61 (s, 6H), 1.42 (s, 6H).
f1 (ppm)

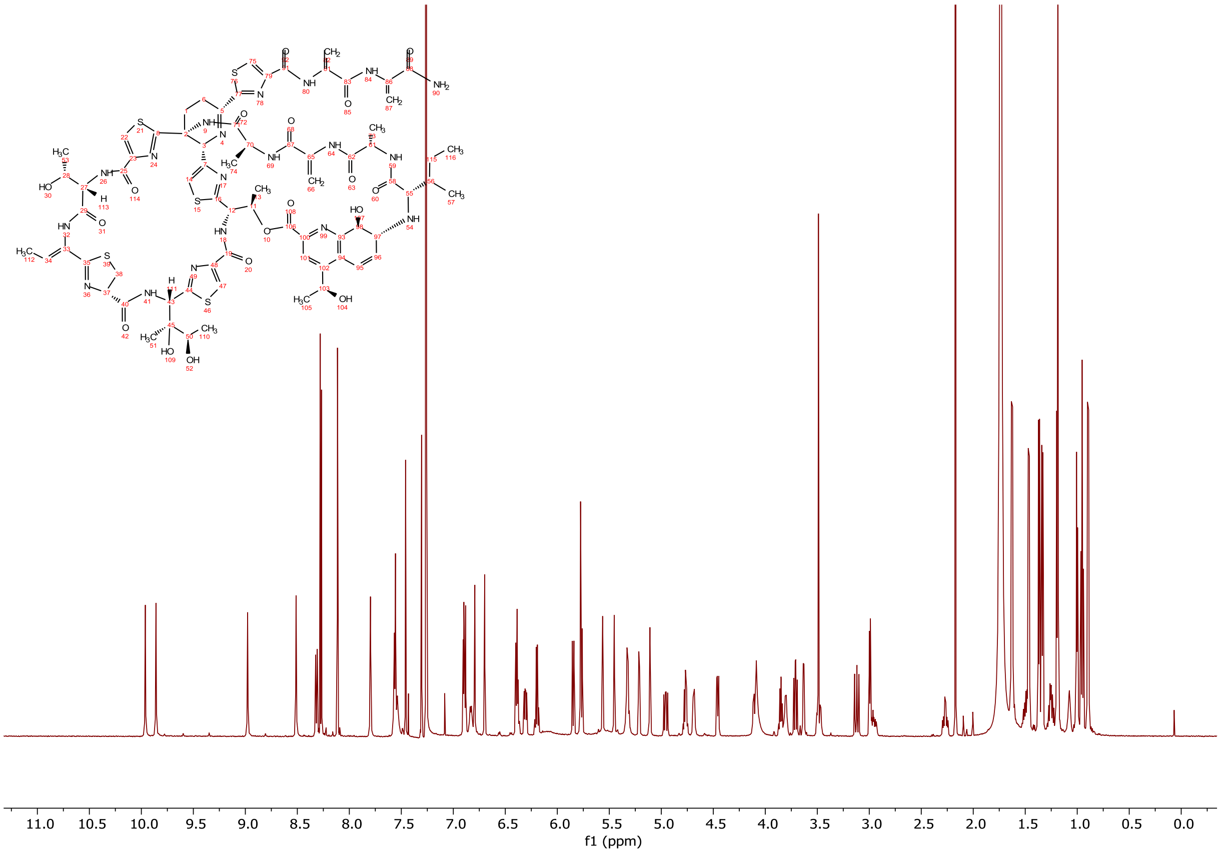

^{1}H NMR spectrum of thiostrepton.

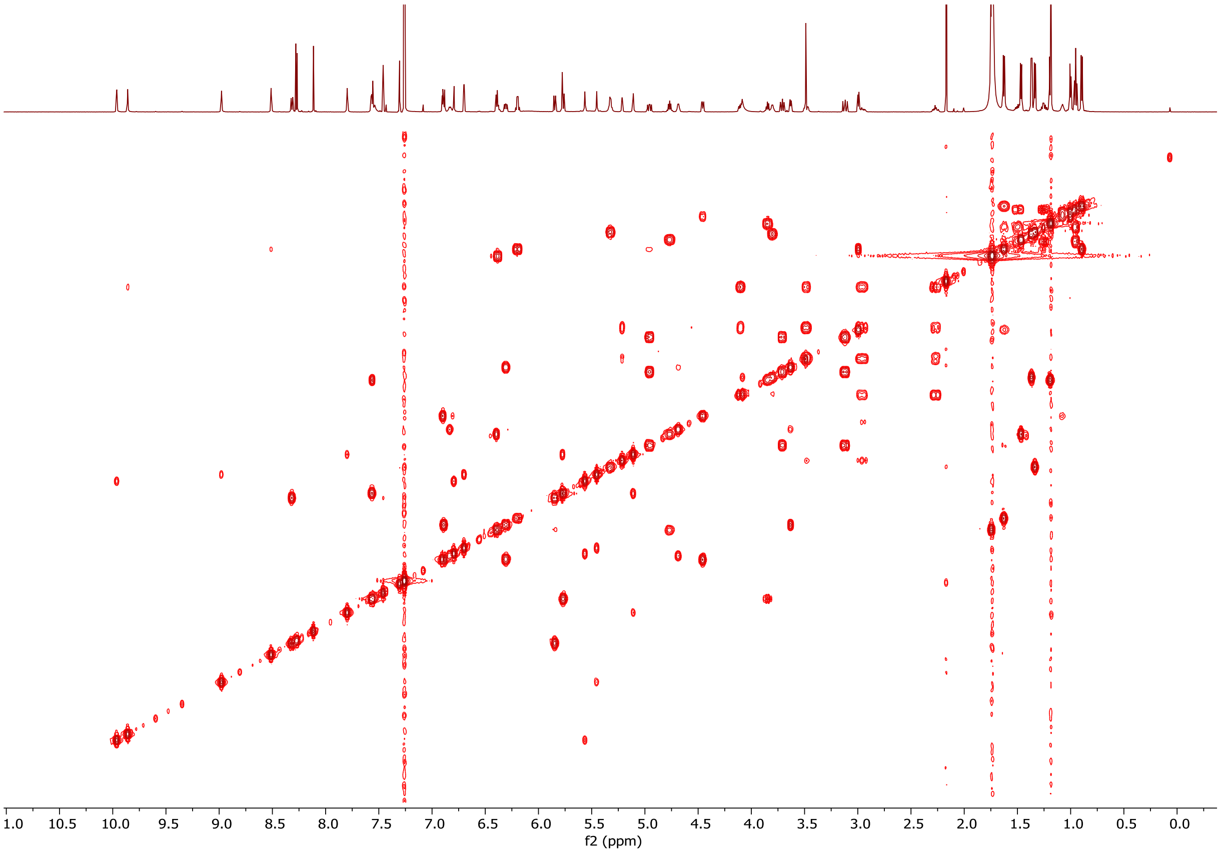

^{1}H-^{1}H COSY NMR spectrum of thiostrepton.

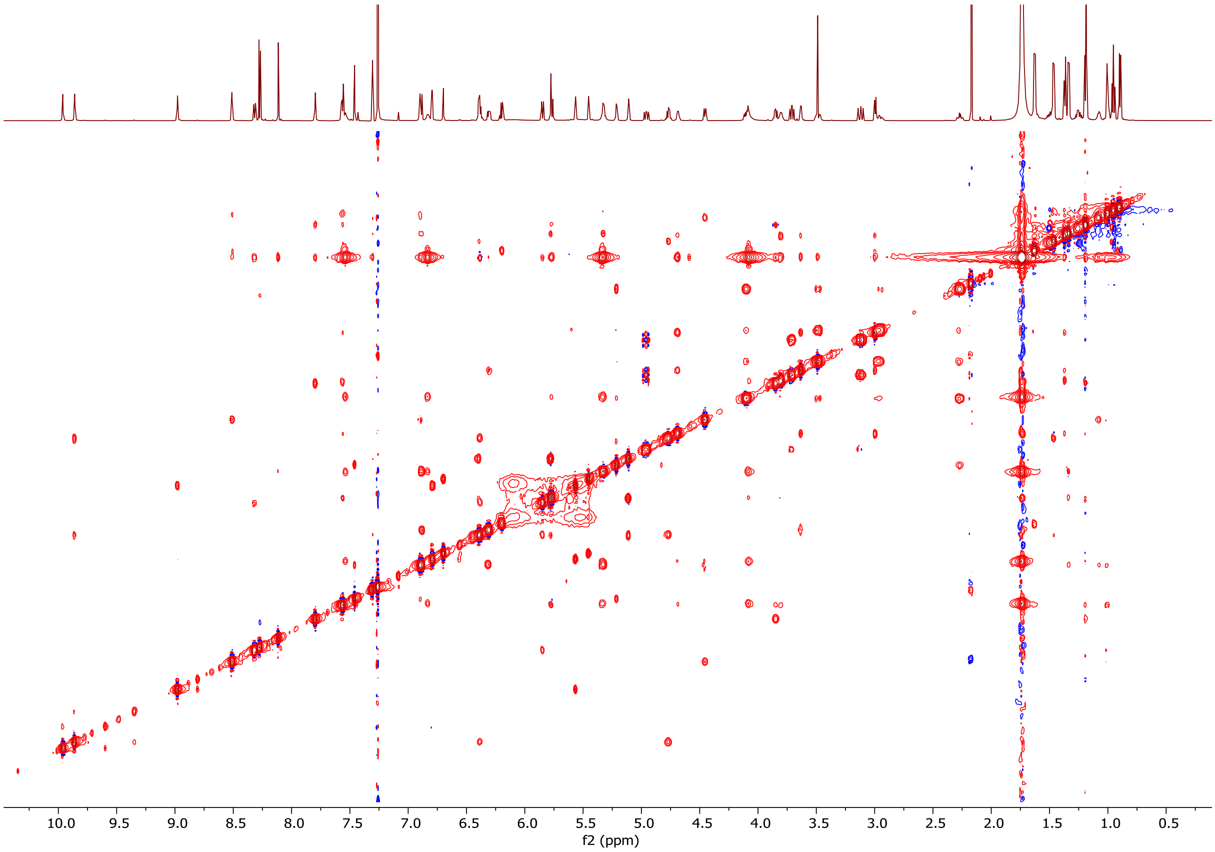

^{1}H-^{1}H NOESY NMR spectrum of thiostrepton.

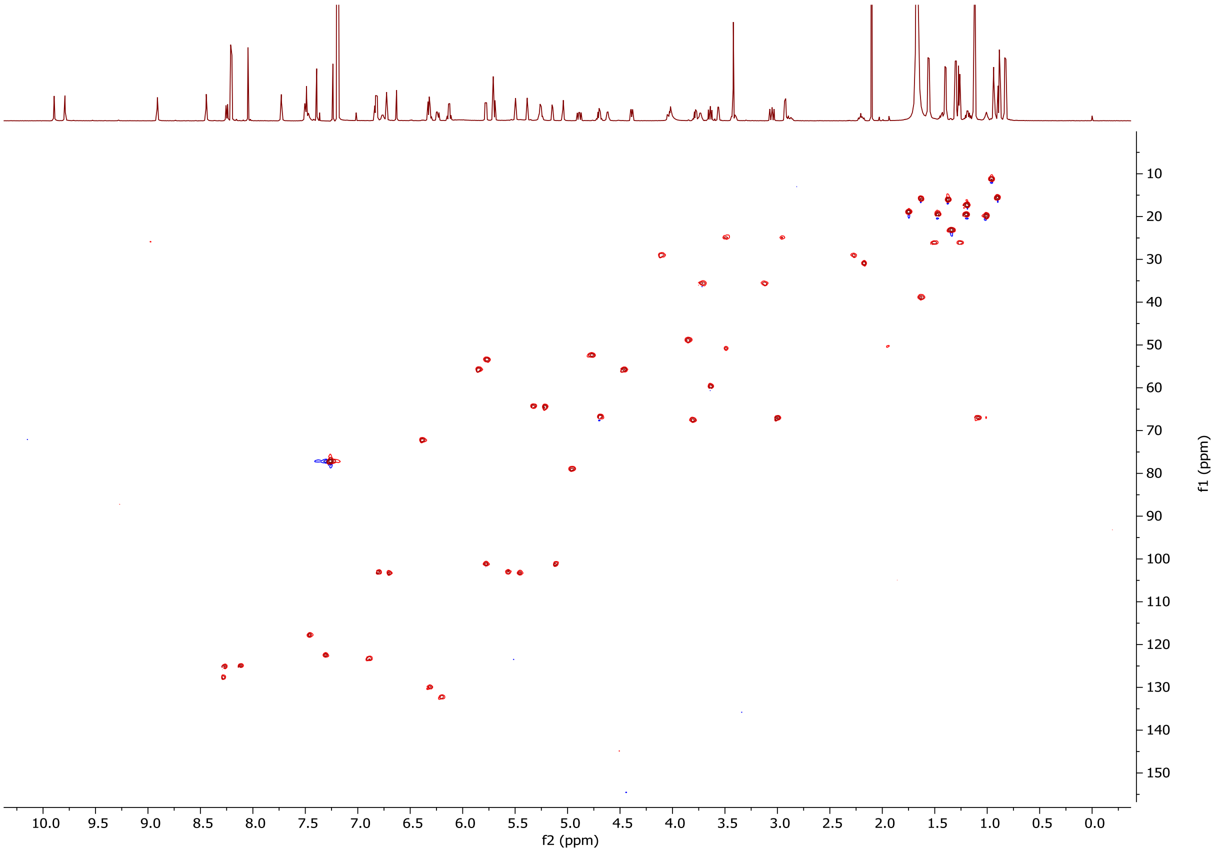

^{1}H-^{13}C HSQC NMR spectrum of thiostrepton.

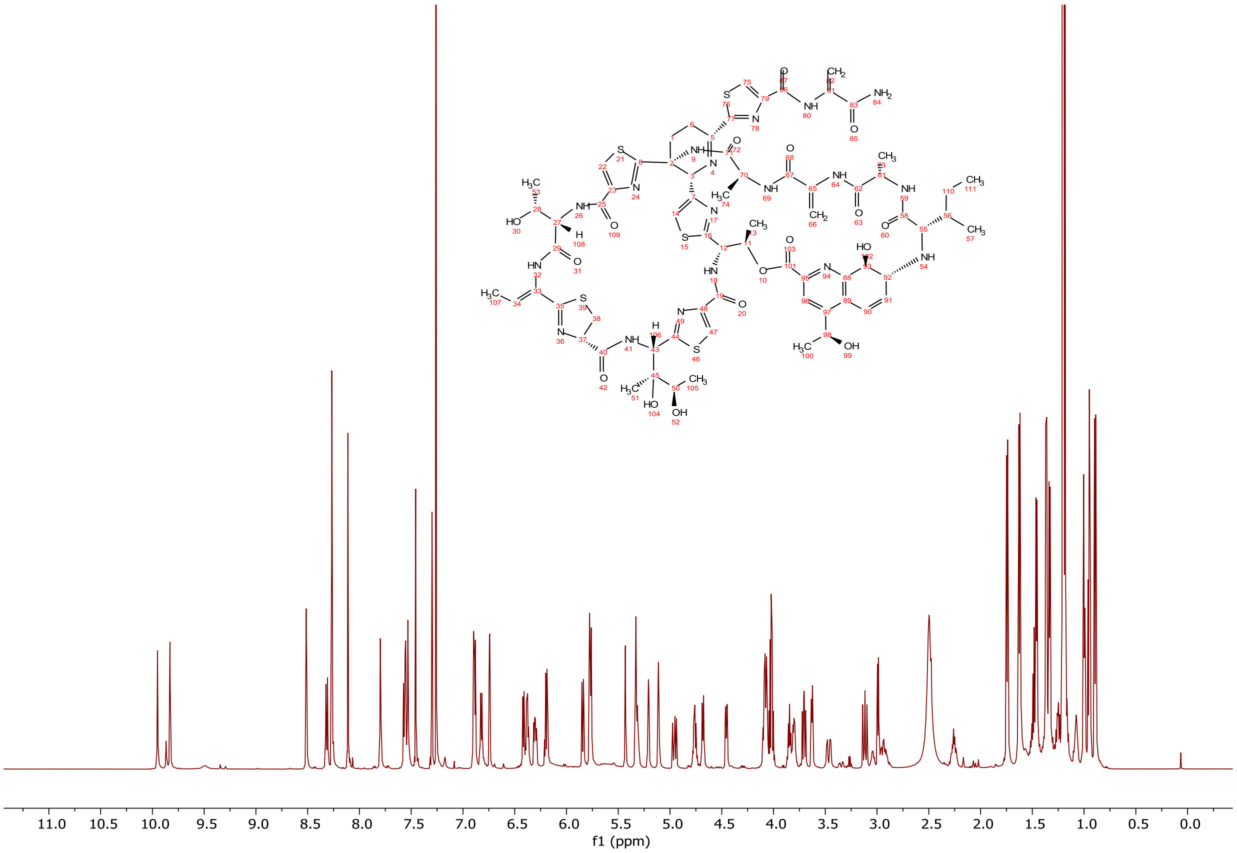

^{1}H NMR spectrum of truncated thiostrepton (3).

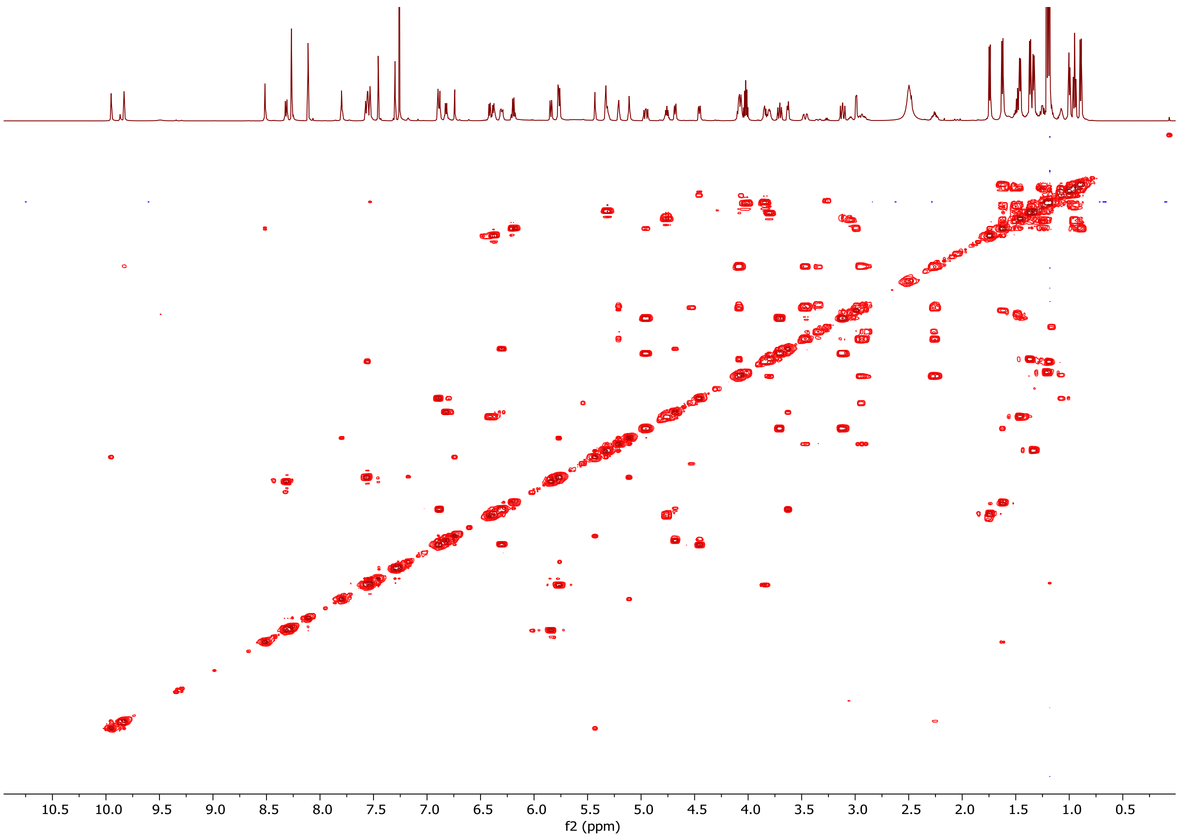

^{1}H-^{1}H COSY NMR spectrum of truncated thiostrepton (3).

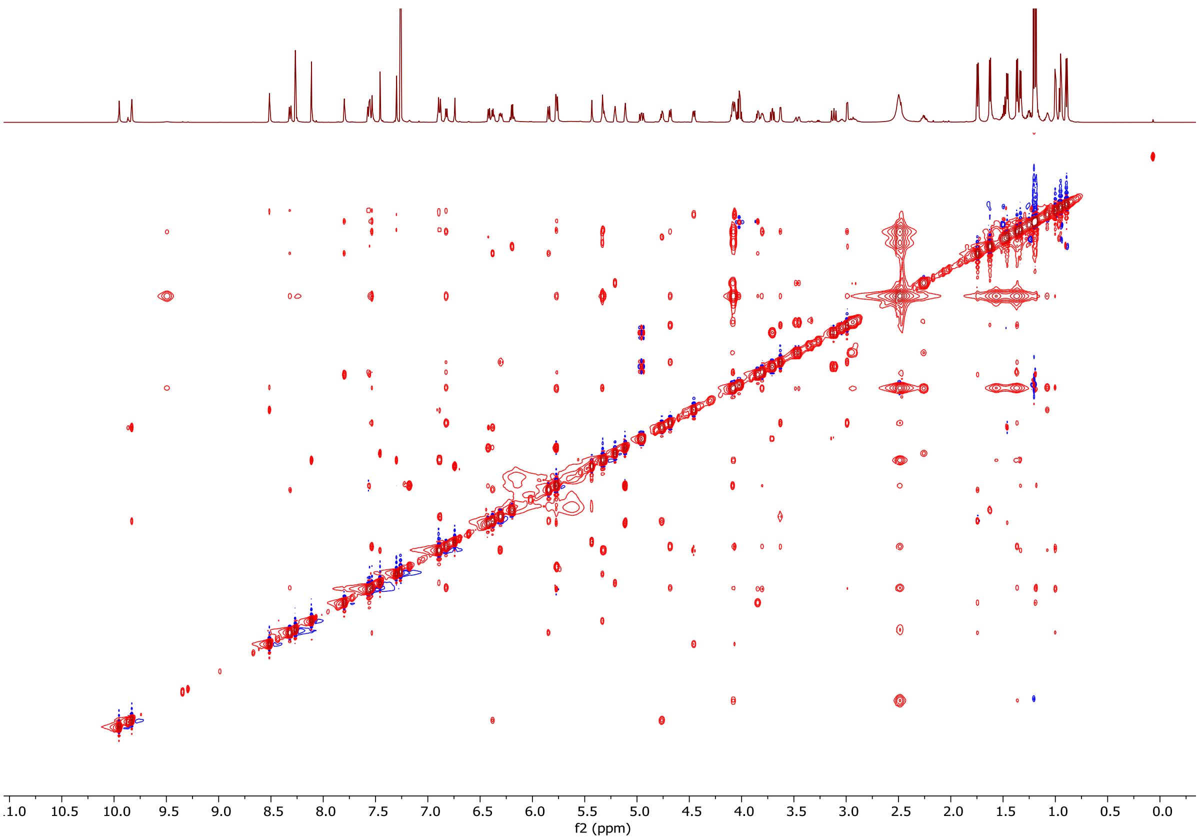

^{1}H-^{1}H NOESY NMR spectrum of truncated thiostrepton (3).

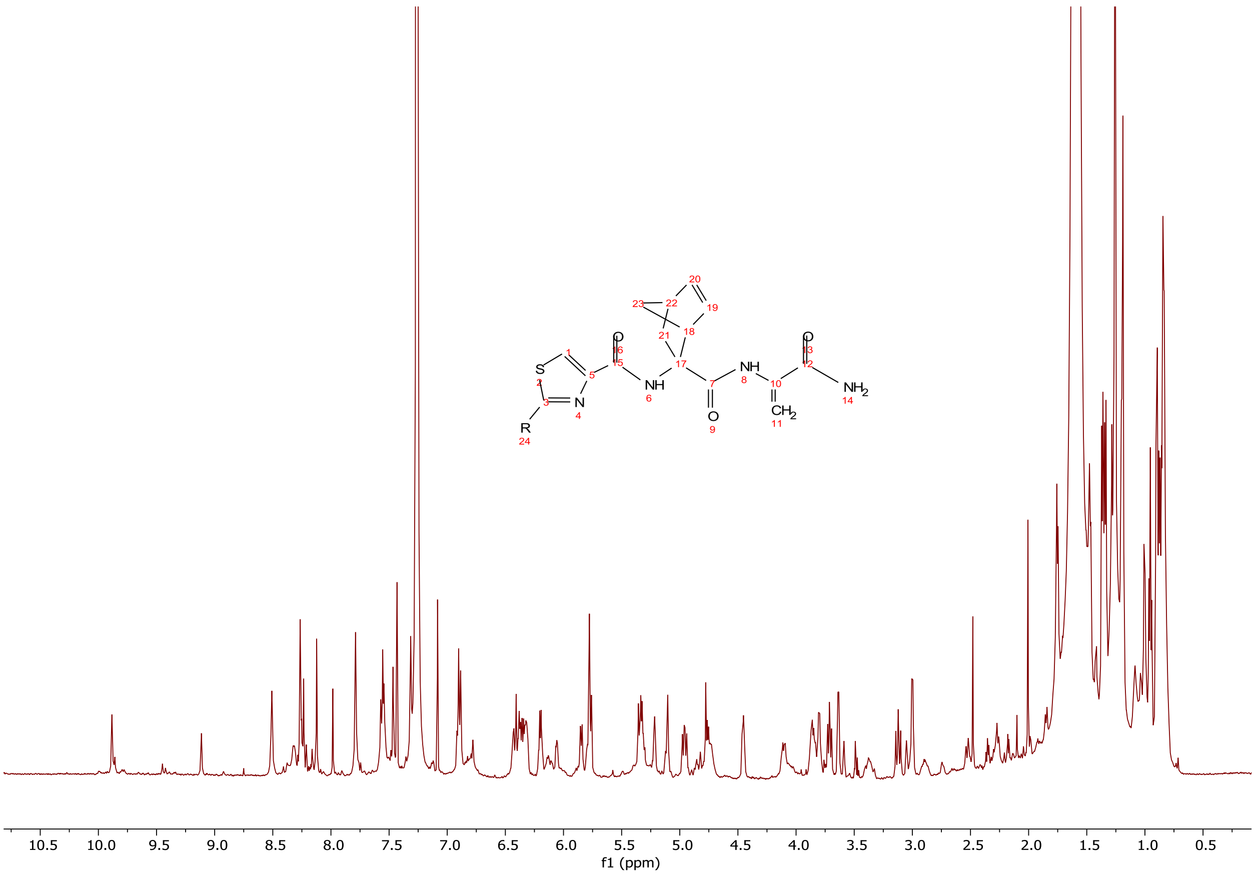

^{1}H NMR spectrum of thiostrepton-Dha16-*endo* (2a).

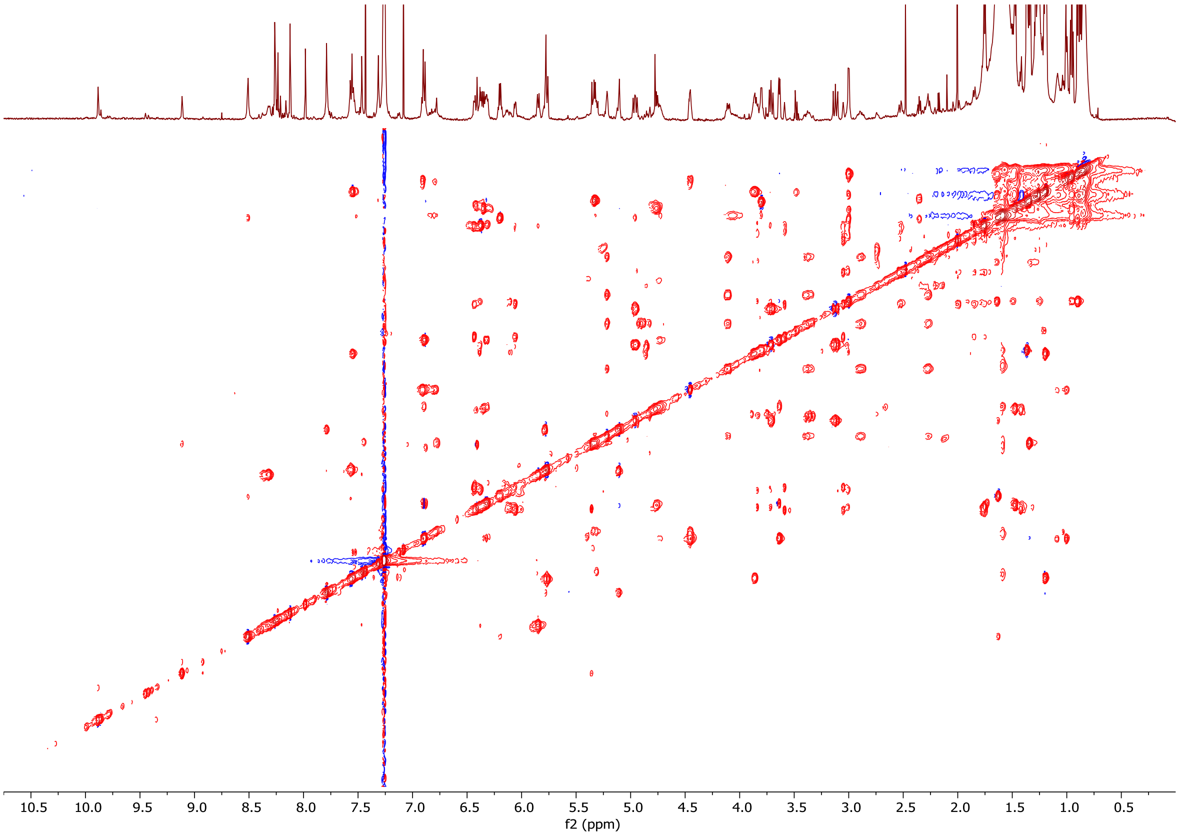

^{1}H-^{1}H TOCSY NMR spectrum of thiostrepton-Dha16-*endo* (2a).

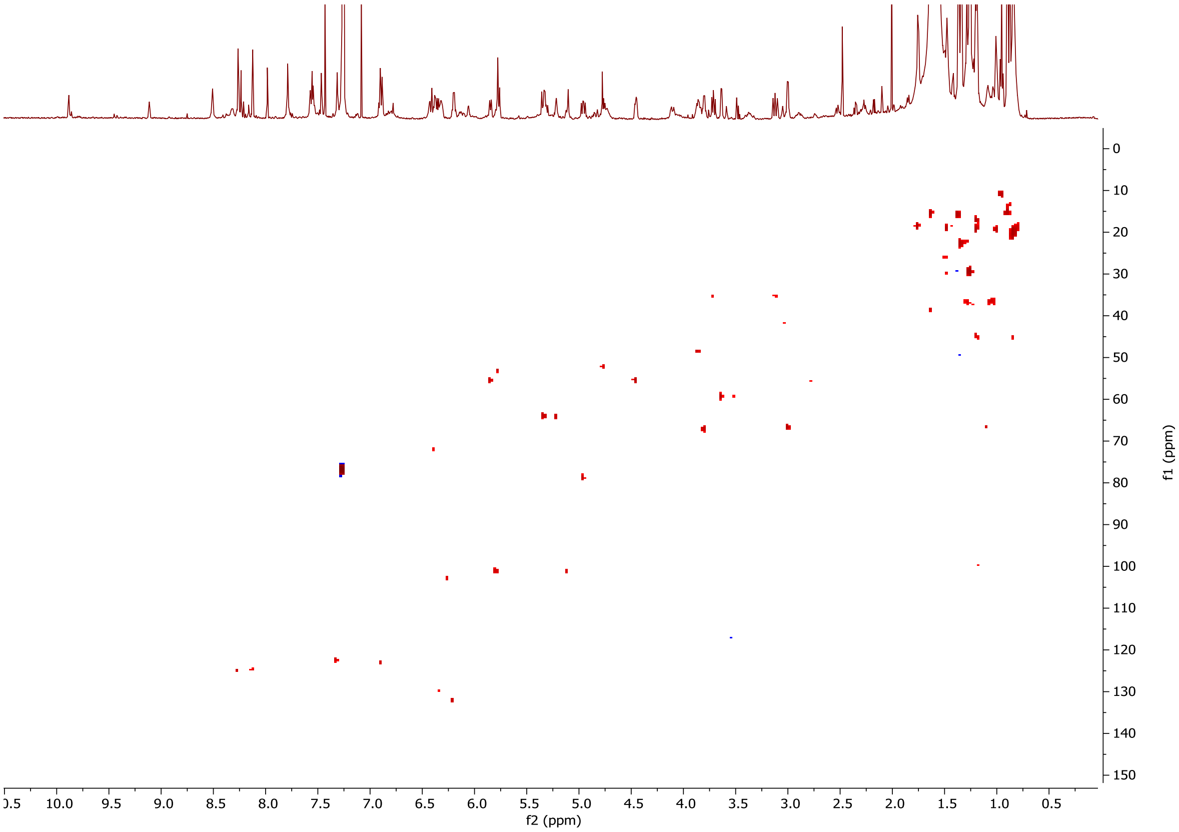

^{1}H-^{13}C HSQC NMR spectrum of thiostrepton-Dha16-*endo* (2a).

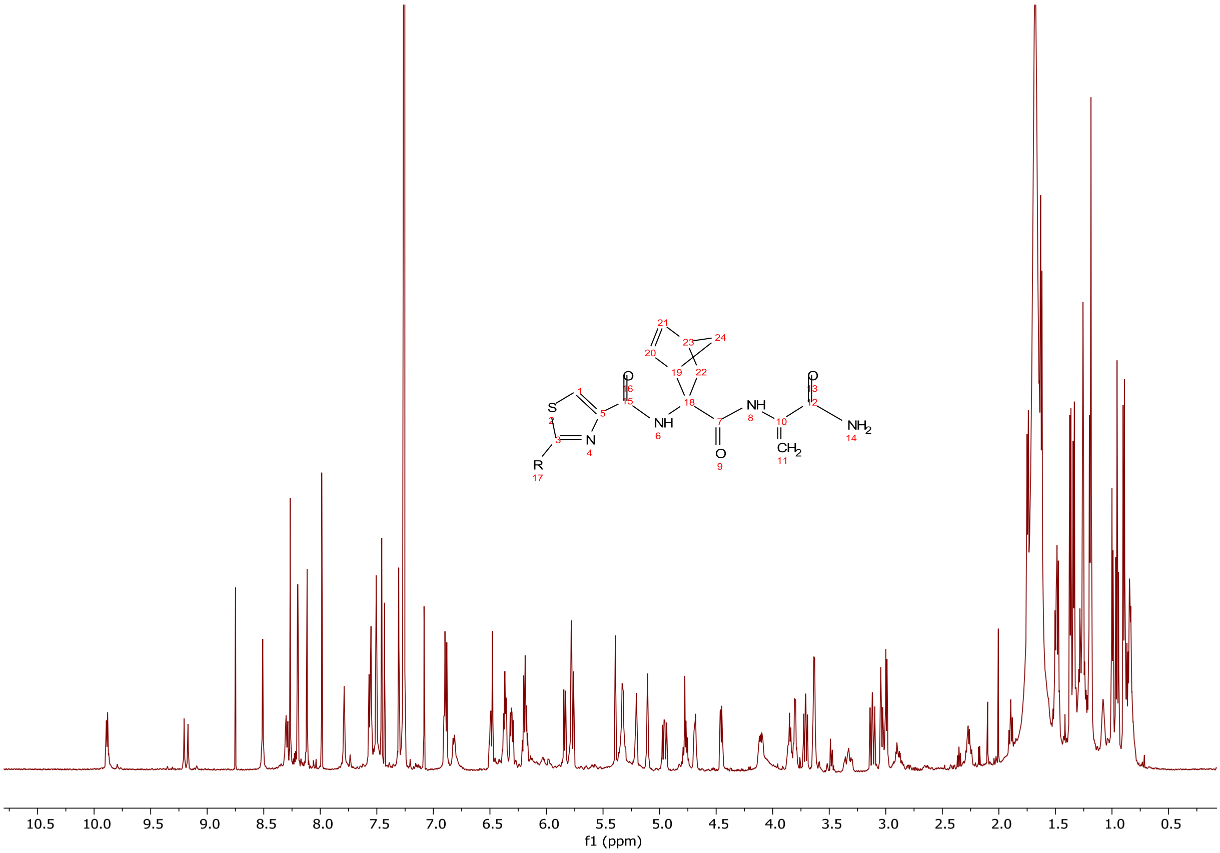

^{1}H NMR spectrum of thiostrepton-Dha16-*exo* (2b).

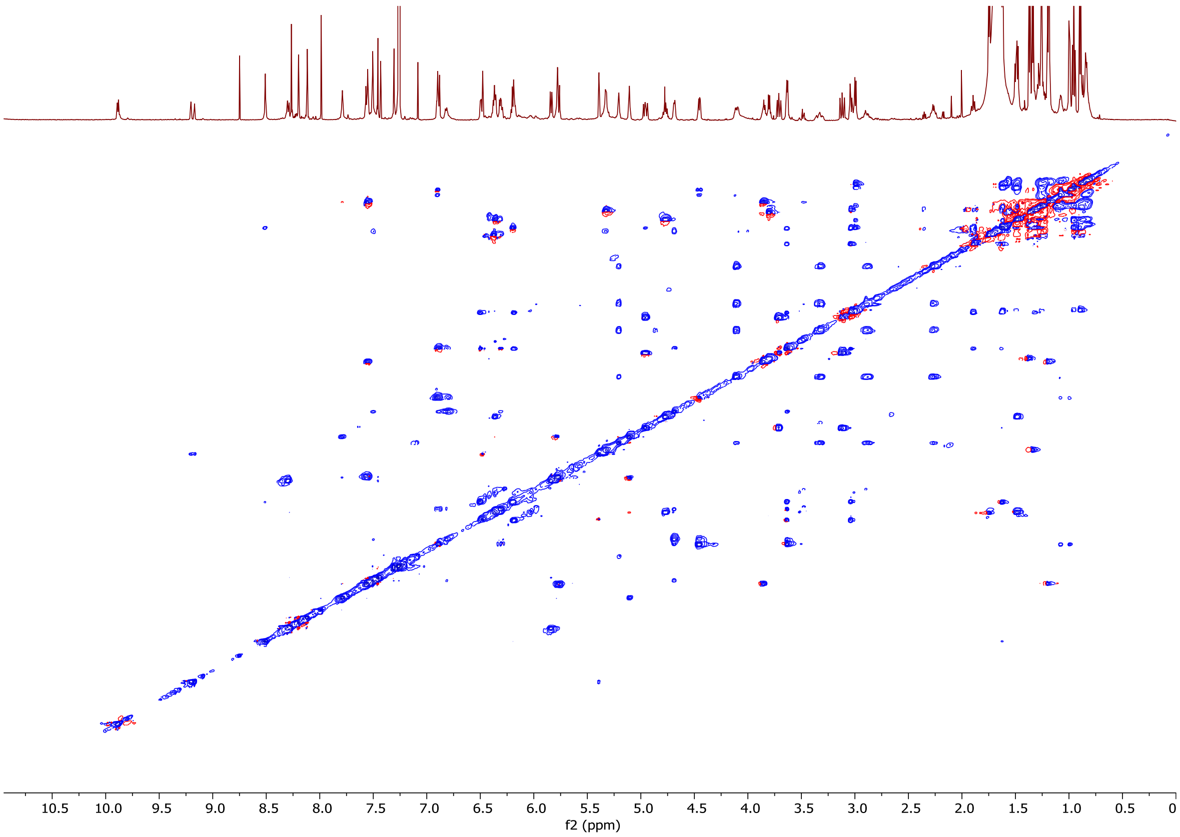

^{1}H-^{1}H TOCSY NMR spectrum of thiostrepton-Dha16-*exo* (2b).

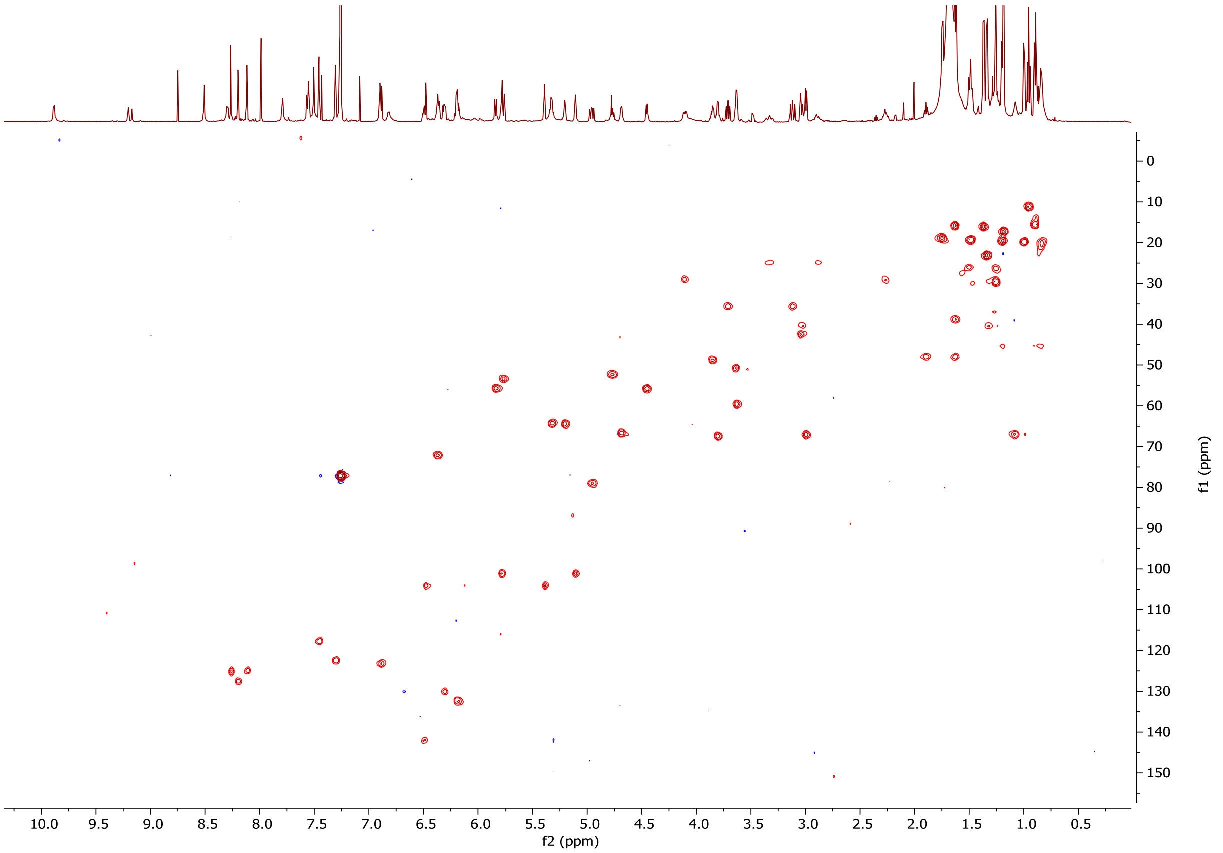

^1H-^{13}C HSQC NMR spectrum of thiostrepton-Dha16-*exo* (2b).

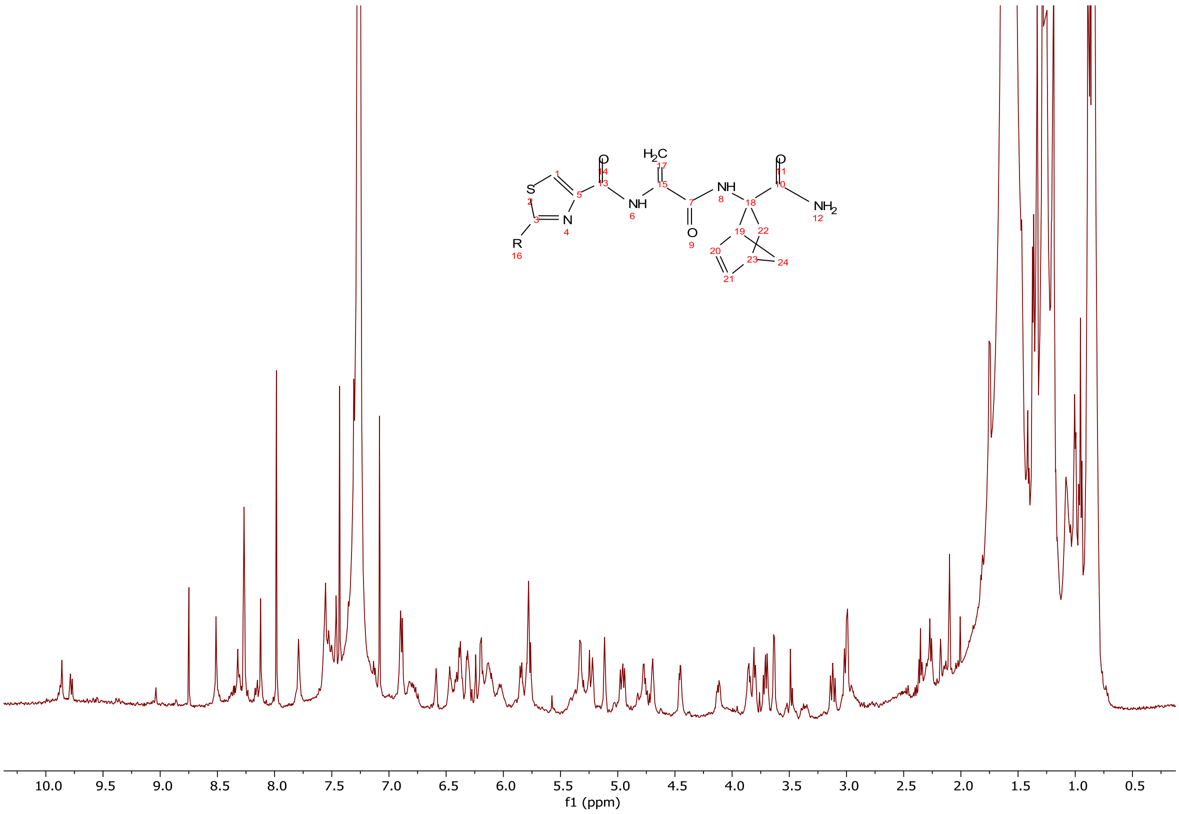

^1H NMR spectrum of thiostrepton-Dha17 (2c).

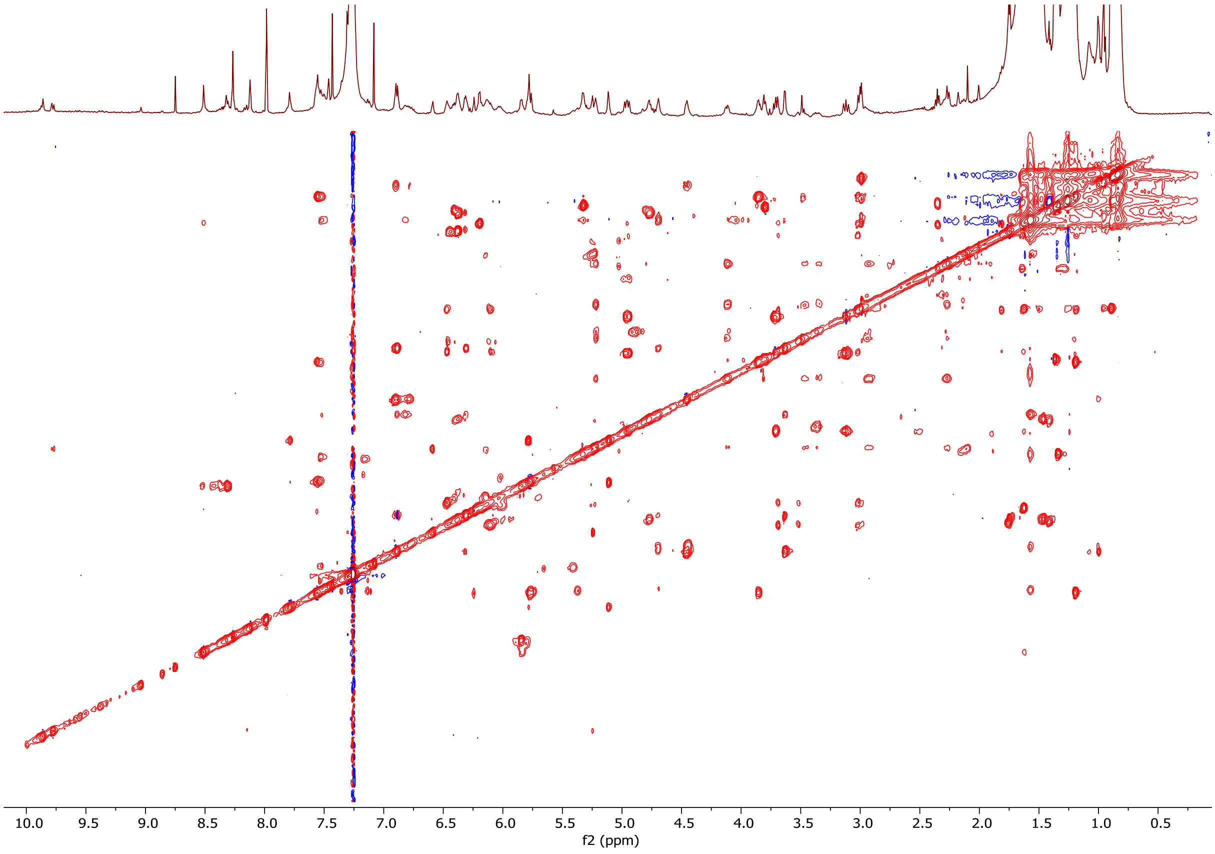

^{1}H-^{1}H TOCSY NMR spectrum of thiostrepton-Dha17 (2c).

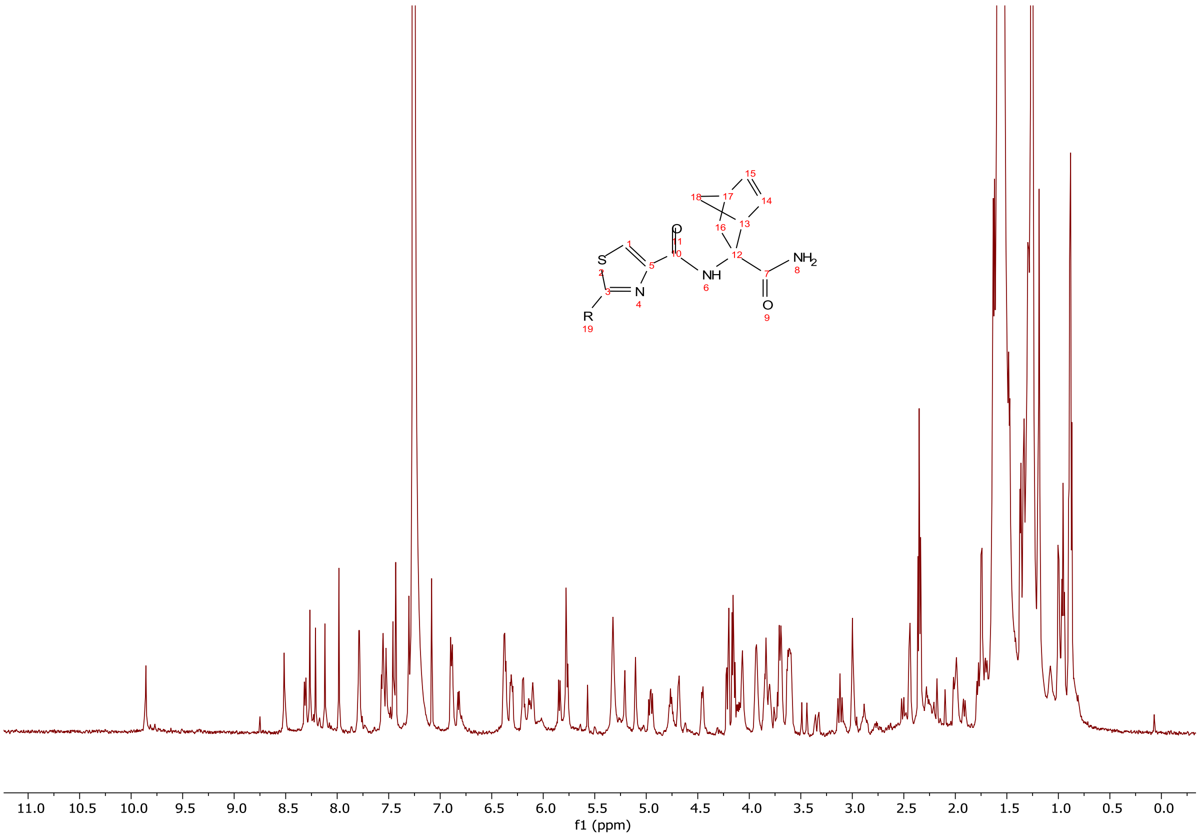

^{1}H NMR spectrum of truncated thiostrepton-Dha16-*endo* (4a).

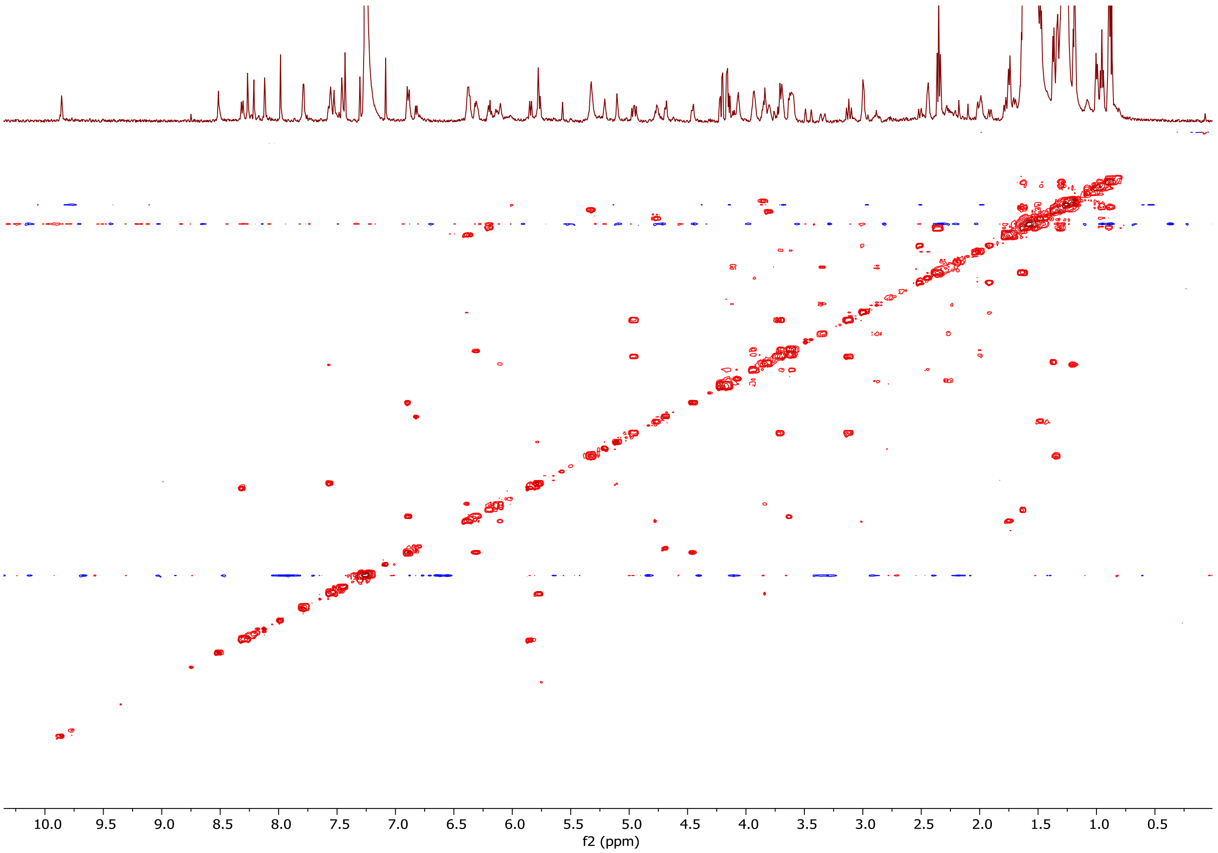

^{1}H-^{1}H COSY NMR spectrum of truncated thiostrepton-Dha16-*endo* (4a).

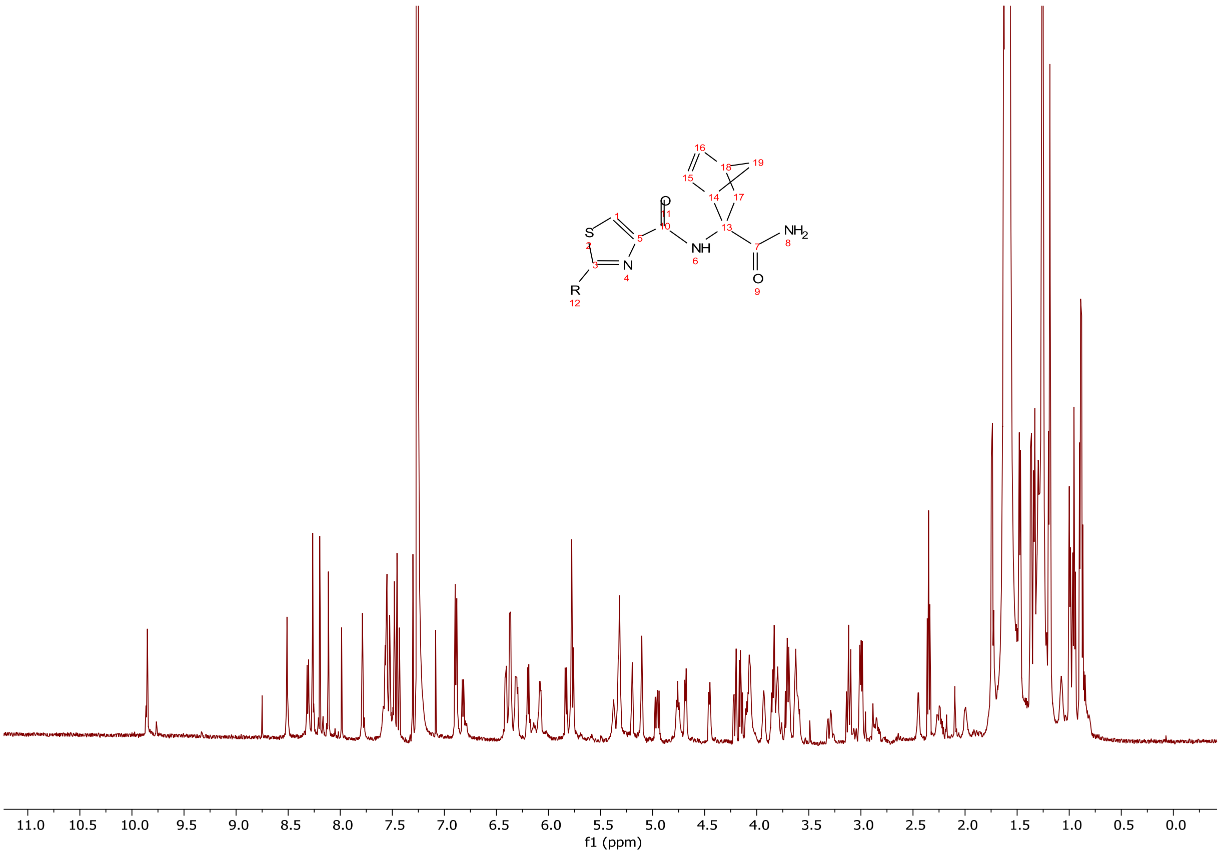

^{1}H NMR spectrum of truncated thiostrepton-Dha16-*exo* (4b).

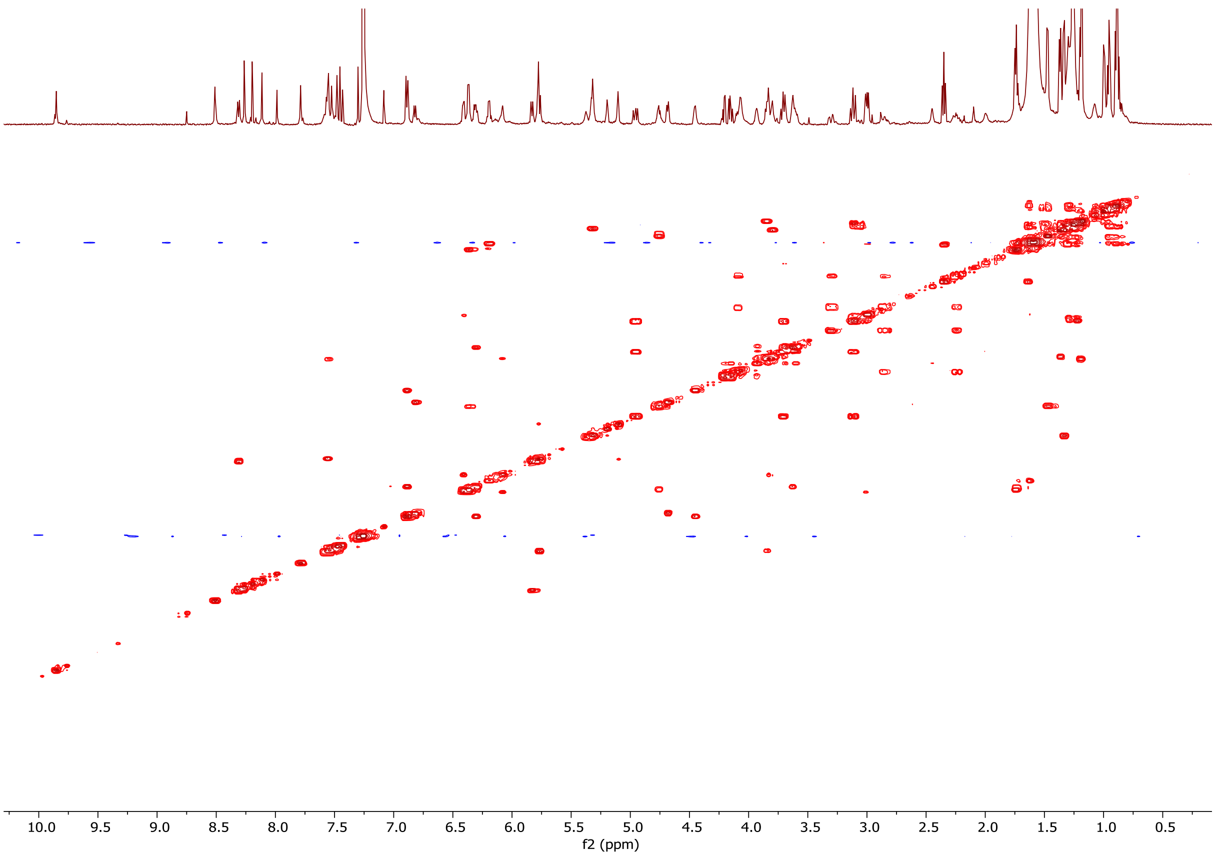

^{1}H-^{1}H COSY NMR spectrum of truncated thiostrepton-Dha16-*exo* (4b).

References

[1] F. Crestey, V. Collot, S. Stiebing, S. Rault, *Synthesis* **2006**, *20*, 3506-3514.
[2] M. Jaegle, T. Steinmetzer, J. Rademann, *Angew.Chem.* **2017**, *129*, 3772–3776; *Angew. Chem. Int. Ed.* **2017**, *56*, 3718-3722.
[3] J. Yang, M.R. Karver, W. Li, S. Sahu, N. K. Devaraj, *Angew. Chem.* **2012**, *124*, 5312–5315; *Angew. Chem. Int. Ed.* **2012**, *51*, 5222 –5225.
[4] R. Kottani, R. A. Valiulin, A. G. Kutateladze, *PNAS* **2006**, *103*, 13917-13921.
[5] J.C.T. Carlson, L.G. Meimetis, S.A. Hilderbrand, R. Weissleder, *Angew. Chem.* **2013**, *125*, 7055–7058; *Angew. Chem. Int. Ed.* **2013**, *52*, 6917 –6920.
[6] I. Wiegand, K. Hilpert, R.E.W. Hancock, R. E. W., *Nat. Protoc.* **2008**, *3*, 163–175.
[7] CLSI. Methods for Dilution Antimicrobial Susceptibility Tests for Bacteria That Grow Aerobically. Approved Standard-Tenth Edition. CLSI document M07-A10. (2015). doi:10.4103/0976-237X.91790.
